# Structure-Based Design and Synthesis of Stapled ^10^Panx1 Analogues for Use in Cardiovascular Inflammatory Diseases

**DOI:** 10.1021/acs.jmedchem.3c01116

**Published:** 2023-09-13

**Authors:** Arthur Lamouroux, Malaury Tournier, Debora Iaculli, Anne Caufriez, Olga M. Rusiecka, Charlotte Martin, Viviane Bes, Laureano E. Carpio, Yana Girardin, Remy Loris, Andrés Tabernilla, Filippo Molica, Rafael Gozalbes, María D. Mayán, Mathieu Vinken, Brenda R. Kwak, Steven Ballet

**Affiliations:** † Research Group of Organic Chemistry, Departments of Chemistry and Bioengineering Sciences, Vrije Universiteit Brussel, Pleinlaan 2, B-1050 Brussels, Belgium; ‡ Department of Pathology and Immunology and Geneva Center for Inflammation Research, Faculty of Medicine, University of Geneva, Rue Michel-Servet 1, CH-1211 Geneva, Switzerland; § Research Unit of In Vitro Toxicology and Dermato-Cosmetology, Department of Pharmaceutical Sciences, Vrije Universiteit Brussel, Laarbeeklaan 103, 1090 Brussels, Belgium; ∥ ProtoQSAR SL, Centro Europeo de Empresas Innovadoras, Parque Tecnológico de Valencia, Avda. Benjamin Franklin 12, 46980 Paterna, Spain; ⊥ Structural Biology Brussels, Department of Biotechnology, Vrije Universiteit Brussel, Pleinlaan 2, B-1050 Brussels, Belgium; ## Centre for Structural Biology, VIB, Pleinlaan 2, 1050 Brussels, Belgium; ∇ MolDrug AI Systems SL, c/Olimpia Arozena 45, 46018 Valencia, Spain; ○ CellCOM Research Group, Instituto de Investigación Biomédica de A Coruña, Servizo Galego de Saúde, Universidade da Coruña, 15071 A Coruña, Spain

## Abstract

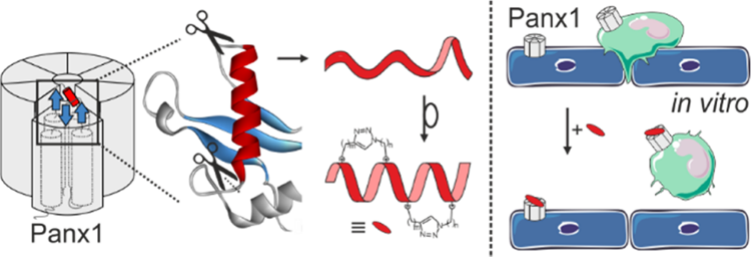

Following a rational design, a series of macrocyclic (“stapled”) peptidomimetics of ^10^Panx1, the most established peptide inhibitor of Pannexin1 (Panx1) channels, were developed and synthesized. Two macrocyclic analogues **SBL-PX1-42** and **SBL-PX1-44** outperformed the linear native peptide. During *in vitro* adenosine triphosphate (ATP) release and Yo-Pro-1 uptake assays in a Panx1-expressing tumor cell line, both compounds were revealed to be promising bidirectional inhibitors of Panx1 channel function, able to induce a two-fold inhibition, as compared to the native ^10^Panx1 sequence. The introduction of triazole-based cross-links within the peptide backbones increased helical content and enhanced *in vitro* proteolytic stability in human plasma (>30-fold longer half-lives, compared to ^10^Panx1). In adhesion assays, a “double-stapled” peptide, **SBL-PX1-206** inhibited ATP release from endothelial cells, thereby efficiently reducing THP-1 monocyte adhesion to a TNF-α-activated endothelial monolayer and making it a promising candidate for future *in vivo* investigations in animal models of cardiovascular inflammatory disease.

## Introduction

Inflammation is a complex process involved in various cardiovascular pathologies, including cardiac ischemia-reperfusion injury and atherosclerosis, which still represent a major therapeutic challenge. One of the key mediators of inflammation is extracellular adenosine triphosphate (ATP), typically derived from necrotic or apoptotic cells.^1^ An initial step in the inflammatory cascade includes the binding of specific ligands to Toll-like receptors at the plasma membrane of endothelial cells, lining the inner surface of blood vessels, and of leukocytes such as neutrophils and macrophages. This activates the nuclear factor kappa B (NF-κB) signaling pathway, which drives the expression of immature forms of interleukin-1β (IL-1β) and IL-18.^2^ Meanwhile, P2X7 receptors become activated by extracellular ATP, leading to ionic fluxes that induce an augmentation of intracellular calcium and a reduction of intracellular potassium and subsequent activation of the NLRP3 inflammasome complex. In response to lowered intracellular potassium concentrations, caspase 1 becomes activated, which in turn induces the cleavage of pro-IL-1β and pro-IL-18 to their mature and active forms as well as their secretion from the cell.^2^

Given the essential role of extracellular ATP in inflammation, pannexin1 (Panx1) channels have emerged as critical players in the regulation of inflammatory responses.^3,4^ Panx1, a channel-forming glycoprotein,^5^ is variably expressed in most mammalian cells, including cardiac- and smooth muscle, endothelium of blood and lymphatic vessels, fibroblasts, and various types of inflammatory cells.^6^ Panx1 channels connect the cytoplasm to the extracellular milieu by allowing the passage of small signaling molecules and metabolites such as ATP, uridine triphosphate (UTP), chloride ions, lactate, and glutamate.^7,8^ The released molecules can signal by targeting surface receptors in a paracrine or autocrine fashion, contributing to intercellular signaling and tissue homeostasis. A variety of receptor-dependent and receptor-independent factors are known to activate Panx1 channels, such as mechanical stress, high extracellular potassium, ischemia, and hypoxia.^9^ In this respect, Panx1 has been shown to interact with the P2X7 receptor, which plays a role in the activation of the inflammasome,^10^ whereas interaction with the alpha-1D adrenoceptor engages a signaling pathway that opens the Panx1 channel and induces vasoconstriction.^11,12^ Finally, irreversible channel opening can be induced by caspase-mediated cleavage of the Panx1 carboxy-terminal domain.^13^

**Figure 1:**
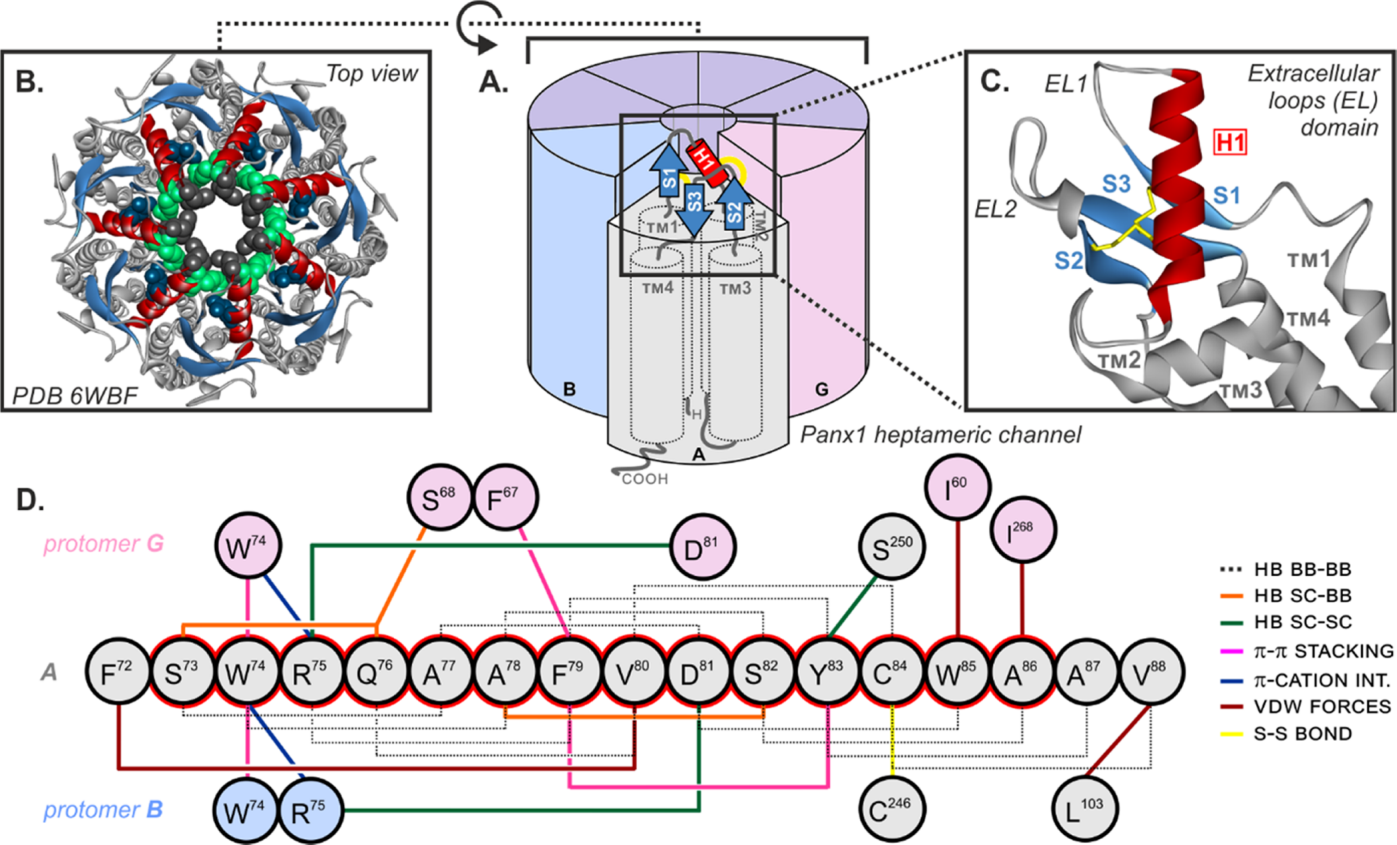
(A) Schematic representation of the heptameric assembly of Panx1. For clarity, only the extracellular domain of subunit A (in gray) is represented. Adjacent subunits B and G are colored blue and pink, respectively. The other subunits are colored in purple. In subunit A, the α-helix (**H1**) and the β-strands (**S1**–**S3**) are represented by a red tube and blue arrows, respectively; the helical transmembrane domains (**TM1**–**TM4**) are represented by gray tubes. Disulfide bonds are represented by yellow lines. (B) Top view of the cryo-EM structure of the extracellular loop (EL) domain of human Pannexin1 (PDB 6WBF).^15^ The α-helix (**H1**) and the β-strands (**S1**–**S3**) are also in red and blue, respectively. Residues Trp^74^, Arg^75^, and Asp^81^, belonging to the helical segment **H1**, are shown in a CPK representation in black, green, and dark blue, respectively. For clarity, only a portion of transmembrane domains (**TM1**–**TM4**) is represented. (C) Zoom-in of the cryo-EM structure of EL. Disulfide bonds established between **S1** and **S3** and between **H1** and **S2** are represented by yellow tubes. (D) Schematic representation of the first extracellular loop (EL1) of Panx1 showing intra- and intermolecular interactions. Residues and their location in the native sequence are represented by spheres. Residues surrounded in red belong to the helical structure **H1**, within which the ^10^Panx1 lead sequence (^74^WRQAAFVDSY^83^) is located.^22,23^ The backbone–backbone (BB–BB), side chain–backbone (SC–BB), and side chain–side chain (SC–SC) hydrogen bonding interactions are represented by black dashed lines and orange and green solid lines, respectively. The other noncovalent interactions, π–π stacking, π-cation, and van der Waals (vdW) forces, are represented by pink, blue, and brown lines, respectively. The disulfide bond (S–S) between residues Cys^84^ and Cys^246^ is represented by a yellow solid line (Supporting Information, Table S1).

More recently, high-resolution cryogenic electron microscopy (cryo-EM) has demonstrated that Panx1 channels are formed by a homotypic arrangement of seven subunits rather than the hitherto assumed six subunits (Figure 1A).^14−17^ At the top of this cylindrical architecture, one can find the extracellular domain forming a compact cap structure (Figure 1B) that can be nearly structurally superposable between different Panx1 cryo-EM–based structures. The channel pore widens when moving toward the intracellular side of the assembly, and finally, the proteins’ *N*- and *C*-termini, together with the cytoplasmic loops, face the intracellular channel entrance. Despite a near-atomic resolution of both extracellular and transmembrane domains, the overall lack of structural information on the intrinsically disordered cytoplasmic domains makes it uncertain whether the reported Panx1 cryo-EM assemblies disclosed open or closed conformational states.^18,19^ Nonetheless, Kuzuya *et al.* have identified an open channel configuration where the *N*-terminal domain of Panx1 acts as a key mediator of the channel gating.^17^ Furthermore, a closure mechanism of Panx1 channels involving the *N*- and *C*-termini has been described by Ruan *et al.* in which both termini deeply protruded in the channel pore under physiological conditions, thereby making it impermeable for ions and small molecules and representing the “closed” state.^15^ Upon Panx1 channel activation, the termini move transiently out of the channel pore, allowing for the release of ATP or other molecules (“open” state). Yet, the lack of structural information describing the long and highly flexible *C*-terminal domain makes it difficult to unambiguously define its role in channel function. Therefore, structural biology remains crucial for further understanding of the molecular functions of these oligomeric channels (*i.e.*, substrate selectivity, channel gating, and ionic permeability) and also offers the possibility of engaging structure-based ligand design. The latter can be realized by means of the detailed and numerous inter- and intramolecular interactions disclosed by such structures, giving insights into the folding of specific subdomains. Of particular relevance to the current study, the extracellular domains of Panx1 (Figure 1B,C) show several structured segments: while the first extracellular loop (EL1) is composed of one β-strand (**S1**) and one α-helix (**H1**), the second extracellular loop (EL2) is composed of two β-strands (**S2** and **S3**). Folding of the three β-strands leads to the formation of an antiparallel β-sheet structure that flanks one side of α-helix **H1**. Contributing to the stability of this compact three-dimensional organization, four cysteine residues cross-link extracellular loops *via* disulfide bridges: the Cys^66^–Cys^265^ and Cys^84^–Cys^246^ pairs connect **S1** to **S3** and **H1** to **S2**, respectively. Even though well-established in the case of connexins,^20^ the exact localization of the disulfide bridges in Panx1 was undetermined prior to the availability of the above-mentioned cryo-EM data.^21^

Remarkably, the extracellular helical sequence **H1** (Figure 1D) overlaps to a great extent with the reference peptide ^10^Panx1 (^74^WRQAAFVDSY^83^), the most established peptide inhibitor of Panx1 channels.^22,23^ Although the mode of action of ^10^Panx1 remains unknown to date (*i.e.*, modulation of gating process *vs* steric block),^24−27^ this Panx1 mimetic decapeptide has proven to be a potent Panx1 channel inhibitor in various cellular assays.^4,28^ As such, ^10^Panx1 has been shown to inhibit Panx1 channel-mediated currents in Panx1-transfected HEK cells and to decrease IL-1β release in human and mouse macrophages.^22^ Additionally, this decapeptide prevented inflammation-induced neuronal cell death in the enteric nervous system^29^ and, *in vivo*, ^10^Panx1 administration reduced liver injury in a mouse model of drug-induced hepatotoxicity and inflammation.^30^ Despite all promising data available in the literature, ^10^Panx1 was suggested to lack significant proteolytic stability due to rapid hydrolysis of scissile amide bonds.^31^ Hence, we hypothesized that its stabilization represents an important step forward in view of more suitable therapeutic lead compounds for inflammation-linked disease treatments.

Next to the stabilization of enzymatically sensitive amide bonds in the peptide backbone, an additional approach to improving the pharmacological properties of a peptide therapeutic relies on the introduction of conformational constraints. Such constraints can provide improved binding with the respective targets (by presenting more homogeneous conformational ensembles) and potentially overcome entropic penalties during binding events. Hence, in the last decades, peptide macrocyclization has proven to be a highly efficient strategy for the stabilization of prevalent secondary structures providing stabilized β-strands,^32,33^ β-turns,^34,35^ β-hairpins,^36,37^ and helical structures.^38,39^ Concomitantly, the resulting macrocyclic peptides present the pharmacological advantage of better resistance to both exo- and endopeptidases as compared to their acyclic counterparts.^40,41^

In the present study, macrocyclization has been carried out under the format of “helical stapling” (*i.e.*, covalent tethering of helical loops) to stabilize the extracellular helical conformation of **H1** in EL1 of Panx1, which largely corresponds to the established ^10^Panx1 inhibitor sequence, as stated above. Using the cryo-EM structures as snapshots of the **H1** domain, a series of triazole cross-linked peptidomimetics were prepared through Huisgen cycloaddition reactions. For this reason, side chain cross-links were inserted at different positions of the peptide backbone while conserving amino acid residues that were believed to be essential for ^10^Panx1 inhibitory activity or oligomeric channel assembly. Moreover, the position and (stereo)chemical nature of the side chain tethers have been varied to determine the optimal linker length and orientation. Next to a conformational analysis by circular dichroism spectroscopy, the conformationally constrained peptides were subjected to *in vitro* ATP release assays, and the proteolytic stability in human plasma was assessed in view of reaching improved half-lives, altogether culminating in highly active analogues with *t*_1/2_ values exceeding 24 h.

## Results and Discussion

### Design and Synthesis of the Peptide Mimetics: First Generation

The extracellular domain of Panx1 is an essential part of the protein channel due to its presumed involvement in ion permeation and ligand binding.^15,42^ Although the underlying molecular mechanisms remain partially unclear, analysis of the Panx1 cryo-EM structures has pinpointed a crucial role for some extracellular residues in Panx1 channel activity and especially those belonging to the helical structure **H1** found in the extracellular domain (Figure 1D). For clarity, the residues are named based on their relative positions in the native sequence of human Panx1 hereafter. As recently documented,^18^ the indole side chains of the Trp^74^ residues of each protomer form a hydrophobic ring lining the wall of the channel pore entrance in the heptameric structures (Figure 1B). This tight ring acts as a size-selective filter for the permeation of crossing entities. Additionally, the guanidine moieties of the Arg^75^ residues within each protomer establish two electrostatic interactions with the neighboring α-helix **H1**, first, *via* a cation–π interaction with the aforementioned Trp^74^ side chain and second, *via* a salt bridge with the carboxylate of Asp^81^ (Figure 1D and Supporting Information, Figure S1). This interprotomer network of interactions contributes to the stabilization of the channel entrance and is also responsible for the anion selectivity of Panx1 channels.^43^ Furthermore, previous electrophysiology and mutagenesis studies have identified Trp^74^, Arg^75^, and Asp^81^ residues, as well as Gln^76^ residue, as key elements for ligand binding.^44,45^

The Panx1 channel inhibitor ^10^Panx1 mimics a decameric peptide segment from helix **H1** (Figure 1D). Although there is no evidence of the binding mode of ^10^Panx1 to its target, the peptide is expected to perform its inhibitory activity by either sterically hindering the ion channel itself or interfering with the stability of the channel assembly. Recently, our group has published the first structure–activity relationship (SAR) study of the reference peptide inhibitor ^10^Panx1, identifying scissile amide bonds and key side chains within ^10^Panx1.^31^ Briefly, based on *in vitro* ATP release measurements, residues Gln^76^ and Asp^81^ were revealed to be the most crucial for ^10^Panx1 inhibitory capacity. Alanine substitution of these residues drastically reduced the ATP release from a cell line overexpressing Panx1. To a lesser extent, a nonapeptide and octapeptide, where Trp^74^ and Trp^74^-Arg^75^ residues of ^10^Panx1 were successively omitted, have induced a significant loss of activity compared to the native sequence. Validating thereby our initial assumptions on key residues responsible for ^10^Panx1 inhibition, this SAR study paved the way toward the development of new therapeutically applicable Panx1-peptidomimetics.

**Figure 2:**
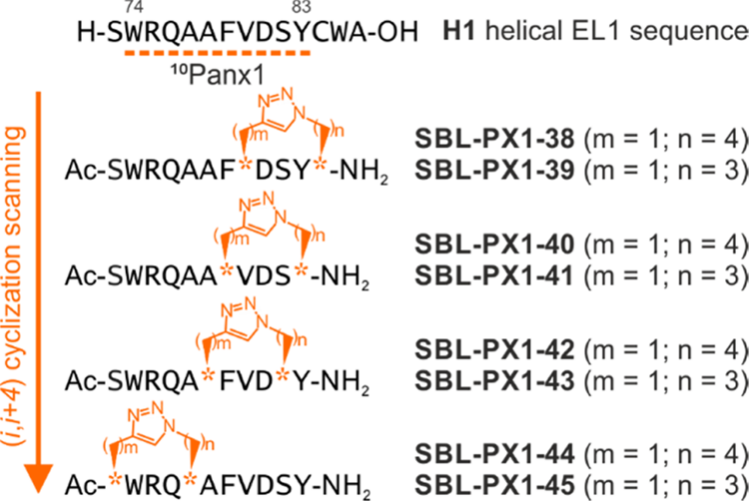
Sequences of the (*i*, *i* + 4) triazole cross-linked ^10^Panx1-based peptidomimetics obtained by 1,3-dipolar copper-catalyzed azide–alkyne cycloaddition (CuAAC). The azidated and alkynylated residues are represented by orange stars, and the corresponding number of their methylene units are written as (*m*, *n*) values. The peptide sequences of **H1** and ^10^Panx1 are written on top.

Side chain-to-side chain macrocyclization is a common approach to preorganizing and maintaining the conformation of natively helical peptide sequences.^38,39^ Common macrocyclization methods include ring-closing metathesis,^46,47^ lactamization,^48,49^ disulfide/thioether bridge formation,^50,51^ and Cu(I)-catalyzed azide–alkyne cycloaddition (CuAAC).^52,53^ The latter technique has received much interest in peptide and protein chemistry as it represents a robust bioorthogonal reaction. The resulting 1,2,3-triazole motif displays structural and electronic properties similar to those of an amide bond, and its introduction into the peptide backbone has already given potent ligands for a broad spectrum of therapeutically relevant targets.^54^ To develop triazole-stapled peptidomimetics, several (*i*, *i* + 4) positions within the ^10^Panx1 sequence have been screened with the prospect of conserving the supposedly important residues Trp^74^, Arg^75^, Gln^76^, and Asp^81^, whereas other positions have been systemically substituted by non-proteogenic amino acid residues harboring azidated and alkynylated side chains needed for macrocyclization (Figure 2). Notwithstanding, the length of the peptidomimetics has slightly been extended beyond the initial ^10^Panx1 sequence. At the *N*-terminal extremity, the native polar amino acid Ser^73^ has been included to maintain a specific hydrogen bonding established between the oxygen of the hydroxyl group of Ser^73^ and the amide hydrogen of Gln^76^ (Figure 1D). As can be seen in the different cryo-EM structures (Supporting Information, Figure S1), this specific noncovalent side chain–backbone interaction creates a natural capping effect contributing to the stabilization of the resulting α-helix **H1**.^55^ At the other extremity, amino acid Cys^84^ has not been included in our design to avoid undesired oxidation or dimerization. As mentioned, Cys^84^ is involved in a disulfide bond with Cys^246^ in the native human Panx1 protein; therefore, only its relative *i* + 4 position was used to reach the corresponding stapled sequence. Of note, the presented staple screening strategy positions the tethers on all faces of the helical ^10^Panx1 peptide backbone (Supporting Information, Figure S2) and also allows to encompass residues that are located at both peptide extremities within the triazolyl macrocycle.

The synthesis of peptide mimetics was performed by solid-phase peptide synthesis (SPPS) using the standard Fmoc/*t*-Bu strategy. To build our panel of stapled peptides, two amino acids located at the *i* and *i* + 4 positions of the extended ^10^Panx1 backbone were at first replaced by azidated derivatives (azido-lysine, with *n* = 4 or azido-ornithine, with *n* = 3, Figure 2) and an alkyne-bearing residue (propargylglycine, with *m* = 1). Subsequently, these non-proteogenic side chains were connected on a solid support using the Cu(I)-mediated Huisgen 1,3-dipolar cycloaddition reaction (“click” reaction)^56^ under mild conditions. As such, 8 macrocyclized analogues of ^10^Panx1 were prepared as *N*-terminally acetylated and *C*-terminal carboxamide derivatives to (i) ensure increased proteolytic stability against amino- and carboxypeptidases, respectively,^31^ and to (ii) maintain the amide context of this peptide segment when extracted from a protein structure. Almost all linear precursors were readily converted to the desired triazole cross-linked peptide mimetics. The only exception was compound **SBL-PX1-41**, for which a dimeric side product was generated alongside the desired product—both on solid support and in solution. Because this latter sequence did not provide a workable basis for future peptide optimizations, it was decided not to test it *in vitro*.

### *In Vitro* Assessment of Inhibitory Capacity of Stapled ^10^Panx1 Analogues

**Figure 3:**
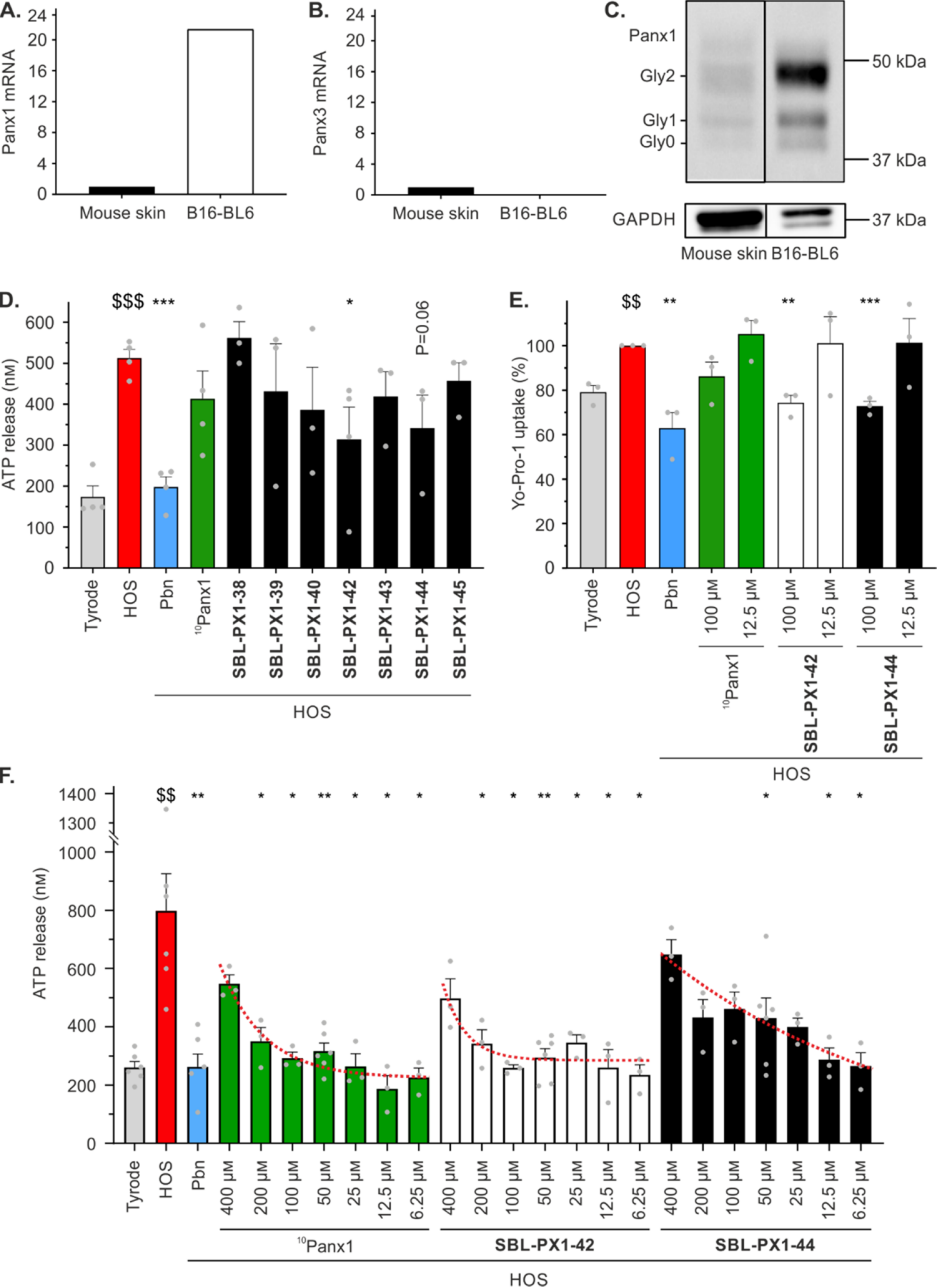
Inhibitory effects of (stapled) ^10^Panx1 analogues on Panx1 channel-dependent ATP release and Yo-Pro-1 uptake in B16-BL6 cells. (A–B) Relative Panx1 (A) and Panx3 (B) mRNA expression levels in B16-BL6 cells normalized to the expression levels in mouse skin, which was used as a positive control. (C) Panx1 protein expression in B16-BL6 cells. Mouse skin was used as a positive control. GAPDH was used as a loading control. Of note, the two lanes were obtained from adjacent lanes in the same Western blot. (D) ATP release by B16-BL6 cells with stapled ^10^Panx1 analogues after 30 min HOS stimulation. Cells were incubated with 100 μM of ^10^Panx1 or stapled analogues. (E) Yo-Pro-1 uptake by B16-BL6 cells with **SBL-PX1-42** and **SBL-PX1-44** after 30 min HOS stimulation. The reference peptide ^10^Panx1 as well as the **SBL-PX1-42** and **SBL-PX1-44** analogues were tested at 100 and 12.5 μM. (F) Concentration–response assay of ATP release by B16-BL6 cells with ^**1**0^Panx1, **SBL-PX1-42**, and **SBL-PX1-44** after 30 min of HOS stimulation. ^10^Panx1 or stapled analogues were used at concentrations ranging from 400 to 6.25 μM. Tyrode conditions represent basal ATP release. HOS induces receptor-independent Panx1 channel opening. The well-known Panx1 channel inhibitor Pbn (2.5 mM) was used as a reference compound. Gray dots represent individual experiments. Data are shown as mean ± SEM ^$^*P* value compared to Tyrode conditions, *P value compared to HOS conditions.

To assess the extent to which preorganization of the peptide’s helical conformation correlated with Panx1 channel inhibitory potency, *in vitro* ATP release and Yo-Pro-1 uptake measurements were performed on B16-BL6 cells, a tumor cell line expressing endogenously high levels of Panx1.^57^ The presence of Panx1 mRNA in B16-BL6 cells was confirmed by real-time reverse transcriptase quantitative polymerase chain reaction analysis (Figure 3A). Likewise, the absence of Panx3 mRNA in these cells was verified, assuring that ATP release and Yo-Pro-1 measurements would not be affected by this membrane channel (Figure 3B). Unlike the other pannexin family members, Panx2 channels are considered to not traffic to the plasma membrane but to remain confined within cytoplasmic compartments of mammalian cells,^58,59^ although a recent study challenges this notion.^60^ Nonetheless, Panx2 mRNA was also not detected in B16-BL6 cells (data not shown). By Western blot analysis, Panx1 typically appears as 3 bands between 41 and 48 kDa, representing the nonglycosylated form (Gly0), the partially (high-mannose) glycosylated form (Gly1), and the complex glycosylated plasma membrane-associated form (Gly2) of Panx1.^61^ The presence of all three glycosylated forms of Panx1 in B16-BL6 cells was confirmed by Western blotting (Figure 3C). To measure ATP release, confluent monolayers of B16-BL6 cells were preincubated for 10 min in a physiological Tyrode’s solution containing control inhibitors of Panx1 channels, in particular, probenecid (Pbn, 2.5 mM) or ^10^Panx1 (100 μM),^62^ or the panel of stapled peptides (100 μM each). Panx1 channel opening was induced in a receptor-independent manner by applying a hypo-osmotic shock (HOS).^9^ A 3-fold increase in ATP release was observed 30 min after the induction of HOS, indicating Panx1 channel opening under these conditions (Figure 3D). While the nonspecific Panx1 channel inhibitor Pbn reduced HOS-induced ATP release by 61% to nearly control conditions (*i.e.*, Tyrode), the reference peptide inhibitor ^10^Panx1 tended to decrease ATP release by 19%.

The stapled ^10^Panx1 analogues (Figure 2) had diverse effects on HOS-induced ATP release (Figure 3D), confirming that changes in the position and length of the triazole linker led to substantial dissimilarities in bioactivity. A total loss of Panx1 channel inhibition was observed with the same peptide sequence when the azido-ornithine precursor (**SBL-PX1-39**; *m* = 1; *n* = 3) was replaced by the azido-lysine precursor (**SBL-PX1-38**; *m* = 1; *n* = 4). On the contrary, two sequences possessing a triazole cross-link made of azido-lysine (*i.e.*, **SBL-PX1-42** and **SBL-PX1-44**; *m* = 1; *n* = 4) gave rise to the best-performing compounds of this series of macrocyclic compounds. In fact, while other ^10^Panx1 stapled peptides did not show significant inhibition compared to the HOS conditions, compounds **SBL-PX1-42** and **SBL-PX1-44** were able to reduce HOS-induced ATP release by 39 and 33%, respectively. Subsequently, ATP release through Panx1 channels was assessed in B16-BL6 cells in a concentration–response assay with the two most promising stapled ^10^Panx1-based inhibitors **SBL-PX1-42** and **SBL-PX1-44** at concentrations ranging from 400 to 6.25 μM (Figure 3F). It was found that compounds **SBL-PX1-42** and **SBL-PX1-44**, together with the reference peptide ^10^Panx1, inhibited ATP release in a concentration-dependent manner. Counterintuitively, a better inhibitory effect was observed when decreasing the concentrations of the peptide inhibitors. Furthermore, while the Panx1 channel inhibitory effects of ^10^Panx1 and **SBL-PX1-42** reached a plateau at concentrations between 100 and 6.25 μM and almost achieved basal levels of ATP release in B16-BL6 cells (*i.e.*, similar to the Tyrode condition), the Panx1 channel inhibitory capacity of **SBL-PX1-44** followed the same pattern, however, without reaching any plateau. Based on these results, the Panx1 channel inhibitors were further tested at lower concentrations to determine the concentration at which the maximal inhibition was no longer reached. No significant inhibition of HOS-induced ATP release in B16-BL6 cells was observed anymore for compound **SBL-PX1-42** at a low nanomolar range (around 10 nM; Supporting Information, Figure S3), proving that this macrocyclic ^10^Panx1 analogue exhibited a “U-shaped” concentration–response effect. U-shaped concentration–response curves have been previously described in the context of NLRP3 inflammasome responses^63^ or when homeostasis is disrupted,^64^ which may be the case in our experiments due to the use of HOS to induce the opening of Panx1 channels. To limit the number of conditions in the next sets of experiments, they were performed using the maximal and minimal concentrations at which ^10^Panx1 and **SBL-PX1-42** displayed the best inhibition of ATP release (*i.e.*, 100 and 12.5 μM).

In addition to their proinflammatory effects as ATP release channels, endothelial Panx1 channels have also been shown to critically regulate the intracellular calcium concentration following treatment with the proinflammatory cytokine TNF-α, thereby inducing a feed-forward effect on NF-κB to regulate IL-1β synthesis.^65^ The ability of the two previously identified lead compounds, **SBL-PX1–42** and **SBL-PX1-44**, to affect the cellular uptake of ions and small molecules through Panx1 channels was evaluated in Yo-Pro-1 uptake assays. Yo-Pro-1 is a small fluorescent dye of 629 Da able to diffuse into cells through open Panx1 channels and emit green fluorescence when binding to nucleic acids in the cells.^66,67^ A representative example showing Yo-Pro-1 uptake over time is given in the Supporting Information (Figure S4). As expected, HOS induced a rapid Yo-Pro-1 uptake, which continued to increase gradually over time. Pbn inhibited Yo-Pro-1 uptake already after 30 s and continued to inhibit Yo-Pro-1 uptake for 30 min to a final lower level than the basal Tyrode conditions (representative for closed Panx1 channels). This strong effect of Pbn after 30 min may be attributed to the nonspecificity of this inhibitor, which has been shown to also inhibit other membrane channels.^68^ While a small nonsignificant inhibition (14%) of Yo-Pro-1 uptake was observed when cells were exposed to 100 μM of ^10^Panx1, the same concentration of the **SBL-PX1-42** or **SBL-PX1-44** analogues decreased Yo-Pro-1 uptake by 26 and 27%, respectively (Figure 3E). However, the lower concentration of ^10^Panx1, **SBL-PX1-42**, or **SBL-PX1-44** (12.5 μM) did not affect the HOS-induced Yo-Pro-1 uptake by B16-BL6 cells. Among other possibilities, the reduced efficacy of the peptidomimetics in Yo-Pro-1 uptake assays, as compared to ATP release assays, may be attributed to the difference in size between ATP and Yo-Pro-1 or to the different repartition of charges in the two molecules. Taken together, our *in vitro* ATP release and Yo-Pro-1 uptake assays demonstrated that **SBL-PX1-42** and **SBL-PX1-44** are promising bidirectional inhibitors of Panx1 channel function able to induce a 2-fold improvement compared to the linear ^10^Panx1 peptide.

### *In Vitro* Assessment of Cytotoxicity and Specificity of Stapled ^10^Panx1 Analogues

The potential cytotoxic effects of the two most promising ^10^Panx1-based channel inhibitors **SBL-PX1-42** and **SBL-PX1-44** were assessed by measuring extracellular lactate dehydrogenase (LDH), a cytoplasmic enzyme that is liberated during cell membrane disruption.^69^ The amount of extracellular LDH was determined in HOS solution containing the nonselective Pbn inhibitor (2.5 mM), or ^10^Panx1 or the stapled analogues at various concentrations (from 100 to 6.25 μM), using the detergent Triton X-100 as a control. As shown in the Supporting Information (Figure S5A), no cytotoxicity was observed for any of the Panx1 channel inhibitors tested, and this is independent of the concentration used.

The cardiomyoblast cell line (H9c2) was chosen to assess the specificity of the stapled ^10^Panx1 analogues. Panx1 mRNA was not found in undifferentiated H9c2 cells or after differentiation into cardiomyocyte-like cells, while it was readily detected in our positive controls (kidney and liver samples; Supporting Information, Figure S5B). The absence of Panx1 protein expression was confirmed by Western blot analysis in both undifferentiated and differentiated H9c2 cells (Supporting Information, Figure S5C) as well as by immunostaining on H9c2 cells (Supporting Information, Figure S5D). ATP release assays on H9c2 cells demonstrated a small increase in extracellular ATP upon HOS stimulation as compared to the basal Tyrode conditions (Supporting Information, Figure S5E). Of note, the low concentration of ATP release in H9c2 cells compared to B16-BL6 cells (47 *vs* 513 nM, respectively, see dotted line in Supporting Information, Figure S5E) cannot be attributed to low cellular content of the metabolite because a high level of ATP was found in the supernatant (1170 nM) when cells were lysed with Triton X-100. No inhibition of ATP release in H9c2 cells was observed upon treatment with the Panx1 channel inhibitors Pbn and ^10^Panx1 and also with compounds **SBL-PX1-42** and **SBL-PX1-44**, suggesting that the inhibitory effects of the tested compounds in B16-BL6 cells were specific to Panx1 channels.

### Linker Optimization: Second-Generation Peptidomimetics

**Figure 4:**
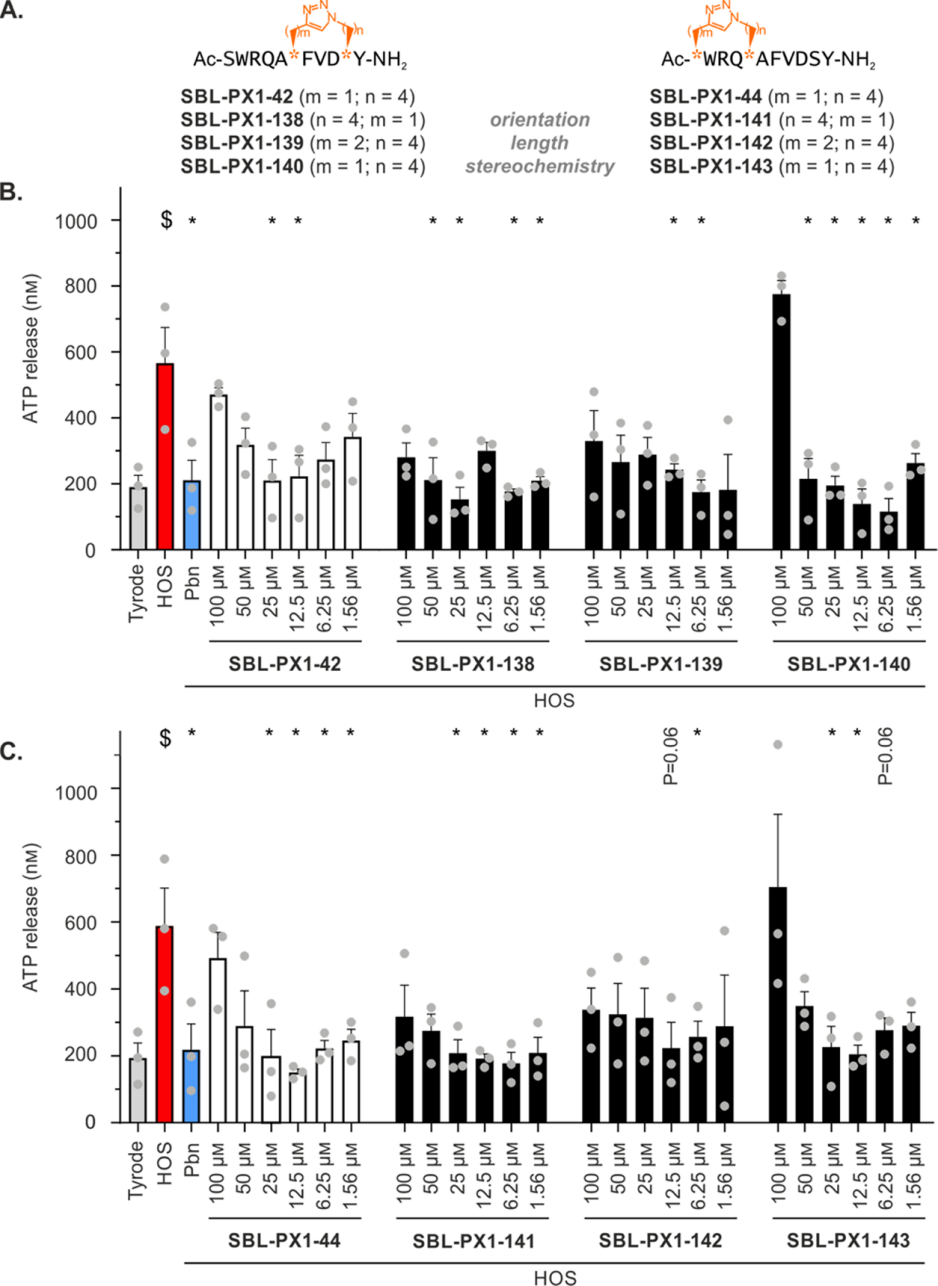
Inhibitory effects of compounds **SBL-PX1-138** to **SBL-PX1-143** on Panx1 channel-mediated ATP release in B16-BL6 cells. (A) Sequences of the analogues of the lead ^10^Panx1-based peptidomimetics **SBL-PX1-42** and **SBL-PX1-44** in which the orientation, the length, and the stereochemistry of the linker have been modulated. The azidated and alkynylated precursor residues are represented by orange stars, and the corresponding number of their methylene units are written as (*m*, *n*) values. Concentration–response assay of ATP release by B16-BL6 cells with analogues of **SBL-PX1-42** (B) and analogues of **SBL-PX1-44** (C) used at concentrations ranging from 100 to 1.56 μM after 30 min HOS stimulation. Tyrode conditions represent basal ATP release. HOS induces receptor-independent Panx1 channel opening. The Panx1 channel inhibitor Pbn (2.5 mM) was used as a reference compound. Gray dots represent individual experiments. Data are shown as mean ± SEM ^$^*P* value compared to Tyrode conditions. *P value compared to HOS condition.

As expected, the initial *in vitro* tests confirmed that the length of the covalent triazole linker and its position within the sequence were determinants for inhibitory activity.^52,70^ Hence, to improve the most promising peptide-based Panx1 channel inhibitors, the linkers were further varied for the two lead peptidomimetics **SBL-PX1-42** and **SBL-PX1-44**. As longer linkers enhanced the inhibitory capacity, compared to shorter ones (*i.e.*, *m* = 1, *n* = 4 *vsm* = 1, *n* = 3, respectively; Figure 3D), an extended version of the linker was introduced in these compounds by adding one additional methylene group (*i.e.*, *m* = 2, *n* = 4). Moreover, because the triazole staple is unsymmetrical, its orientation was reversed, and finally, a variation in the linker’s stereochemistry was introduced by incorporating a d-propargylglycine residue (*i.e.*, d-Pra, *m* = 1, *n* = 4). These changes gave way to a subsequent set of 6 additional analogues (Figure 4A), which were tested at concentrations ranging from 100 to 1.56 μM for their ability to reduce ATP release by B16-BL6 cells (Figure 4B,C).

The variation in the linker’s stereochemistry (**SBL-PX1-140** and **SBL-PX1-143**, respectively) seemed to reinforce the “U-shaped” concentration–response effects of **SBL-PX1-42** and **SBL-PX1-44** analogues, resulting in no inhibition or even promotion of opening of Panx1 channels at the highest concentration used (100 μM). Longer linkers (**SBL-PX1-139** and **SBL-PX1-142**, respectively) overall did not affect the Panx1 channel inhibitory capacity of **SBL-PX1-42** and **SBL-PX1-44**, and the reversal of the staple orientation (**SBL-PX1-138** and **SBL-PX1-141**, respectively) only slightly improved the inhibitory capacity of **SBL-PX1-42** and **SBL-PX1-44** at the highest concentration used. Interestingly, each type of modification performed in both stapled ^10^Panx1 analogues resulted in similar effects on the concentration–response curves. Unfortunately, these effects did not lead to improvements in the inhibitory capacity when compared to **SBL-PX1-42** and **SBL-PX1-44**, but no cytotoxicity was observed in the 6 additional analogues at any of the concentrations used (Supporting Information, Figure S6).

### Circular Dichroism and Molecular Dynamics

**Figure 5:**
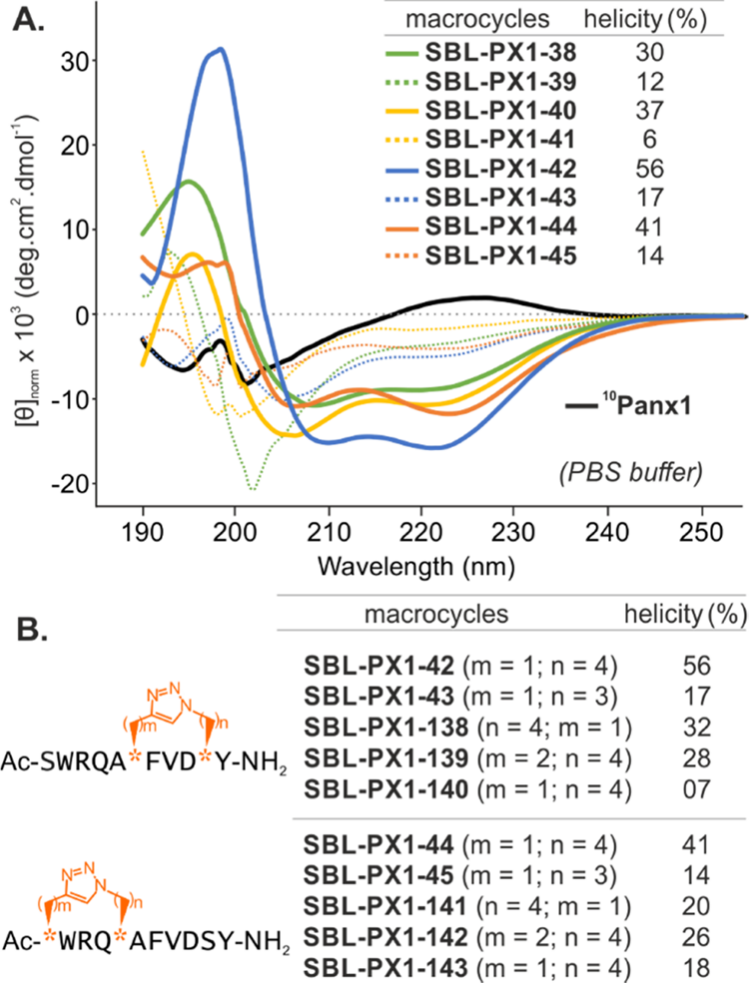
(A) CD spectra of stapled ^10^Panx1-based compounds (colored lines) compared to the linear ^10^Panx1 reference peptide (black line). Triazole cross-linkers made from azido-lysine (*n* = 4) and azido-ornithine (*n* = 3) are represented by full and dashed lines, respectively. (B) Sequences of **SBL-PX1-42** and **SBL-PX1-44** analogues where the length, the orientation, and the stereochemistry at the peptide backbone of the linker have been modulated. The azidated and alkynylated residues are represented by orange stars, and the corresponding number of their methylene units are written as (*m*, *n*) values. Helicity percentages were calculated from the mean residue ellipticity at 222 nm (θ_222_). Compounds were dissolved in PBS (pH 7.4 at room temperature) to reach 100 μM concentrations.

The extent to which helical preorganization was enhanced by stapling was evaluated by circular dichroism (CD) spectroscopy. Generally, an oligopeptide sequence exhibiting a well-defined structure in the folded native protein gives rise to a more disordered structure once extracted from its protein context.^71^ Predictably, the sequences corresponding to the extracellular **H1** domain and the linear peptide inhibitor ^10^Panx1 were observed as random coils in phosphate-buffered saline (PBS) (Figure 5 and Supporting Information, Figure S15).

In contrast, the triazole-stapled peptide library displayed CD spectra in PBS characteristic of typical α-helices with one maximum near 195 nm and two minima at 208 and 222 nm (Figure 5).^72^ Based on the mean residual ellipticity at 222 nm, the ^10^Panx1 stapled peptide analogues displayed a helical conformation ranging from 6 to 56% helicity (Figure 5 and Supporting Information, Figures S16 and S17). Strikingly, enhanced helical propensity was observed for cyclic analogues having linkers made of azido-lysine-bearing precursors (*n* = 4), as compared to linkers made of azido-ornithine containing precursors (*n* = 3). Indeed, while the helical behavior for compounds **SBL-PX1-42**, **SBL-PX1-44**, **SBL-PX1-40**, and **SBL-PX1-38** (*m* = 1, *n* = 4) was assessed by order of descending helicity as 56, 41, 37, and 31%, respectively, shortening the linker by one methylene group (*m* = 1, *n* = 3) dramatically decreased helicity. On the contrary, an extension of the triazole bridges of the two lead peptides **SBL-PX1-42** and **SBL-PX1-44** (*m* = 2, *n* = 4; **SBL-PX1-139** and **SBL-PX1-142**, with 28 and 29% of helicity, respectively) was not able to improve the reference helicity (Figure 5B and Supporting Information, Figures S19 and S20). Moreover, reversing the linkers’ orientation in both compounds **SBL-PX1-42** and **SBL-PX1-44** (presenting **SBL-PX1-138** and **SBL-PX1-141**, respectively) induced a reduction of their helicity by nearly half. Finally, the largest reduction was noted when unnatural d-Pra residue was introduced within the all-l peptidomimetic **SBL-PX1-42** (**SBL-PX1-140**). Overall, these results have confirmed the determinant influence of the linker nature on peptide backbone conformation.^48,52,56^

To further assess the conformational stability of the lead compound **SBL-PX1-42**, CD measurements at varying temperatures in PBS were carried out (Supporting Information, Figure S18).^73^ Over a large range of temperatures (Δup to 50 °C), the peptidomimetic’s α-helical profile was conserved, a result indicative of high thermal stability. Even after heating up to 90 °C, the peptide was able to restore its original equilibrium state at room temperature. As a complementary stability study, the three-dimensional structures of these peptidomimetics have been analyzed by molecular dynamics (MD). Our results indicated that both linear sequences **H1** and ^10^Panx1 did not preserve their helical behavior once extracted from the protein assembly. Indeed, the intramolecular hydrogen bonding network of these tetradeca- and decapeptide, respectively, progressively collapses over the molecular dynamics time simulation, inducing thereby random coil conformations (Supporting Information, Figures S9 and S10). On the contrary, the introduction of side chains-to-side chains tether has allowed to stabilize the α-helical behavior of the resulting stapled peptides **SBL-PX1-42** and **SBL-PX1-44** to a great extent.

Altogether, these results have confirmed that macrocyclization preorganized an unstructured linear segment into a well-defined and stable conformation. The optimal linker length for helix induction of this series of macrocyclic ^10^Panx1 mimetic peptides has been determined experimentally (*m* = 1, *n* = 4). Therefore, these structural data have confirmed our strategy to conserve the original Pra/Azk combination as optimal precursor residues of triazole linkers for Panx1-targeted mimetics.

### Plasma Stability

**Figure 6:**
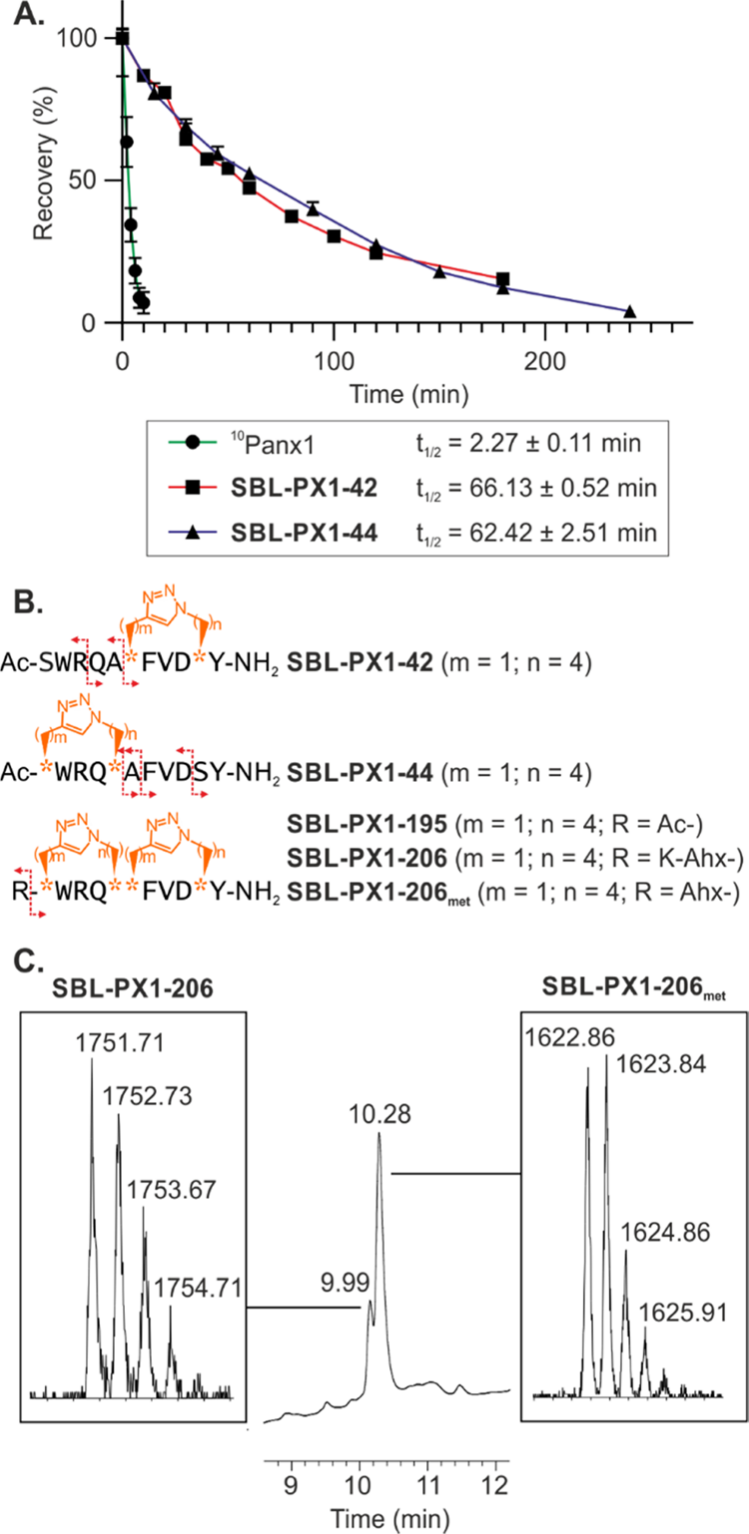
*In vitro* plasma stability of ^10^Panx1-based peptidomimetics. (A) Relative recovery (%) over time of the ^10^Panx1 analogues in human plasma at 37 °C and calculated half-lives. Experiments were performed in triplicate (*N* = 1, *n* = 3), and data are presented as mean ± SD. (B) Sequences of ^10^Panx1 and mono- and bicyclic ^10^Panx1 stapled compounds. The azidated and alkynylated precursor residues are represented by orange stars, and the corresponding number of their methylene units are written as (*m*, *n*) values. Main cleavage sites are indicated by dashed-lined red arrows. (C) Zoom of the LC-MS spectrum of the *in vitro* plasma stability experiment of **SBL-PX1-206** after 24 h in human plasma at 37 °C. The peaks at 9.99 and 10.28 min correspond to **SBL-PX1-206** (exact mass: 1750.92) and **SBL-PX1-206**_**met**_ (exact mass: 1622.82), respectively. Spectra at different time points can be found in the Supporting Information (Figures S11–S14).

The *in vitro* stability of the reference ^10^Panx1 peptide was previously assessed by our research group.^31^ Having a half-life of less than 3 min (*i.e.*, 2.27 ± 0.11 min) in human plasma, ^10^Panx1 is expected to possess a very low exposure after *in vivo* application. Therefore, the introduction of global conformational constraints within this peptide sequence represents a straightforward approach to enhance its resistance toward protease activity.^74,75^ Indeed, the two lead peptidomimetics **SBL-PX1-42** and **SBL-PX1-44** (Figure 6A) exhibited a 30-fold longer half-life (*i.e.*, 66.13 ± 0.52 and 62.42 ± 2.51 min, respectively) in human plasma. This enhanced proteolytic stability was correlated to the helical folding induced by the side chain-based cross-links. As previously reported,^76,77^ and herein confirmed by computational calculations (Supporting Information, Figure S9), the amide bonds of the peptide chain are sheltered within the helix core and are hence less prone to enzymatic hydrolysis. Following incubation of the compounds in human plasma, metabolite analysis of peptidomimetics **SBL-PX1-42** and **SBL-PX1-44** identified several cleavable amide bonds within the peptide chains (Figure 6B). Most remarkably, the amide bonds within the macrocycle remained intact for several hours. In this respect, scissile amide bonds were located between Trp^74^-Arg^75^ and Ala^78^-Phe^79^ residues in the linear ^10^Panx1 peptide sequence, and these were safeguarded once included within the macrocycle (Figure 6B). However, for both peptidomimetics tested, the amide bond located right next to the tethering bridge was cleaved, which led to subsequent structural improvements to further stabilize the peptidomimetics’ backbone.

### Synthesis, Plasma Stability, and *In Vitro* Assessment of Panx1 Channel Inhibitory Capacity of Double-Stapled ^10^Panx1 Analogues in Endothelial Cells

**Figure 7:**
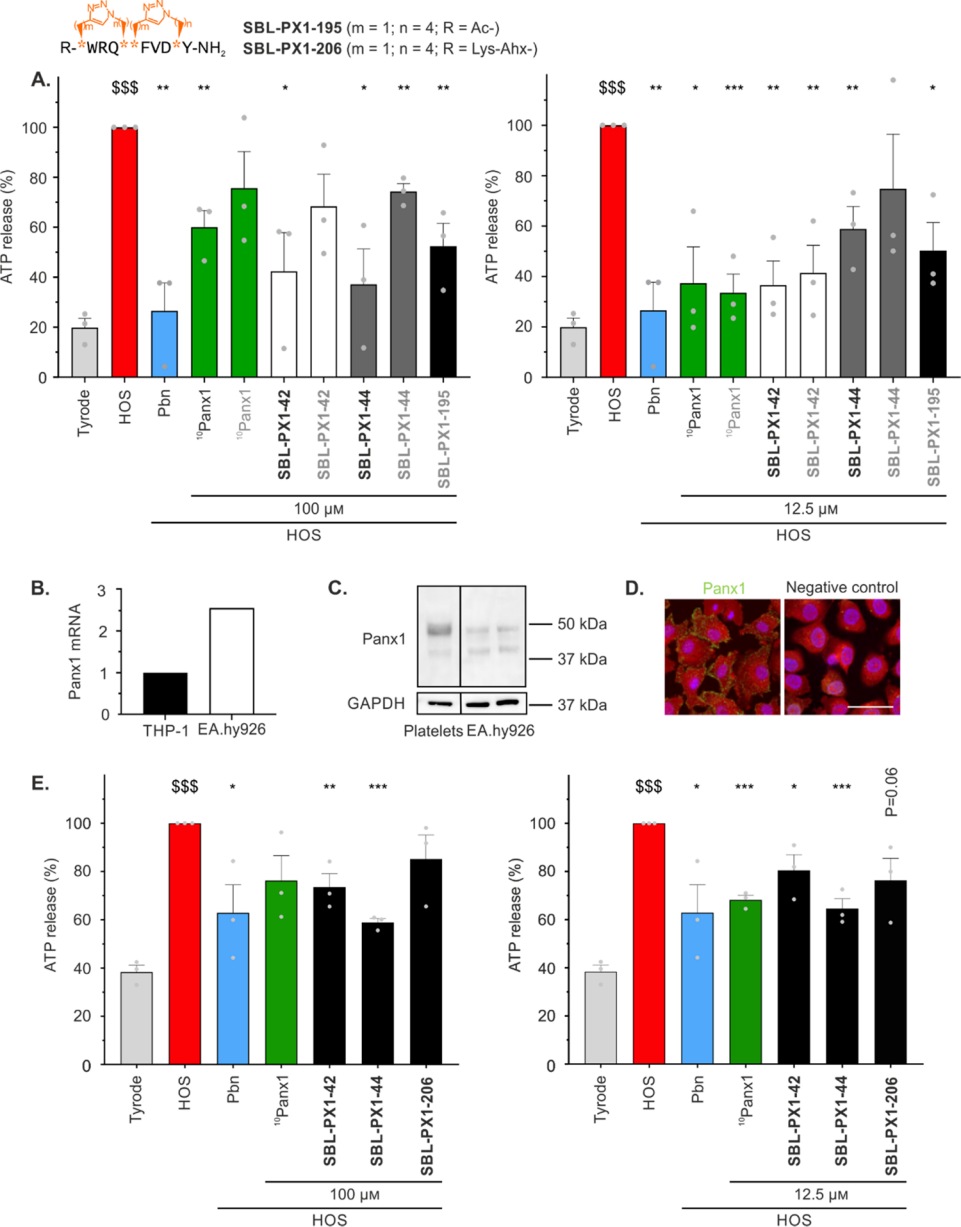
Inhibitory effects of bicyclic compounds **SBL-PX1-195** and **SBL-PX1-206** on Panx1 channel-mediated ATP release in B16-BL6 and EA.hy926 cells. The sequences are written on top where the azidated and alkynylated residues are represented by orange stars, and the corresponding number of their methylene units are written as (*m*, *n*) values. (A) ATP release by B16-BL6 cells with **SBL-PX1-195** after 30 min HOS stimulation. Cells were incubated with 100 μM (left) or 12.5 μM (right) of ^10^Panx1, stapled, and double-stapled analogues. For better comparison with **SBL-PX1-195**, peptides were dissolved in saline solution without (black characters) or with 1% DMSO (gray characters). Tyrode conditions represent basal ATP release. HOS induces receptor-independent Panx1 channel opening. The Panx1 channel inhibitor Pbn (2.5 mM) was used as a reference compound. (B) Relative Panx1 mRNA expression level in the human EA.hy926 endothelial cell line normalized to human THP-1 monocytic cells, which were used as a positive control. (C) Panx1 protein expression in EA.hy926 cells. Human platelets were used as a positive control. GAPDH was used as a loading control. Of note, the two lanes were obtained from the same Western blot but not from adjacent lanes. (D) Representative images of immunostaining for Panx1 (in green) in EA.hy926 cells (left). The absence of a primary antibody was used as a negative control (right). Cells and nuclei were counterstained with Evans Blue (in red) and DAPI (in blue), respectively. Scale bar = 50 μM. (E) ATP release by EA.hy926 cells with **SBL-PX1-206** after 30 min HOS stimulation. Cells were incubated with 100 μM (left) or 12.5 μM (right) of ^10^Panx1, stapled, and double-stapled analogues. Tyrode conditions represent basal ATP release. HOS induces receptor-independent Panx1 channel opening. Pbn (2.5 mM) was used as a reference compound. Gray dots represent individual experiments. Data are shown as mean ± SEM ^$^*P* value compared to Tyrode conditions. *P value compared to HOS condition.

In both lead peptidomimetics **SBL-PX1-42** and **SBL-PX1-44**, the (*i*, *i* + 4) triazole linker was introduced by substituting a combination of native serine and alanine residues (*i.e.*, Ser^73^-Ala^77^ and Ala^78^-Ser^83^, respectively). Because it was observed that the peptide bonds outside of the macrocycle were still prone to enzymatic degradation, a “double-stapled” system^52,78,79^ was considered wherein both staples were inserted to give access to compound **SBL-PX1-195** (Figure 6B). The resulting bicyclic ^10^Panx1-based structure displays two triazole motifs at both faces of the helix, each located at one extremity of the peptide sequence. However, the introduction of two macrocycles within the peptide backbone led to an increase of hydrophobicity and, therefore, **SBL-PX1-195** displayed limited water solubility. Hence, its Panx1 channel inhibitory capacity has been assessed by ATP release measurements in B16-BL6 cells in regular HOS (Figure 7A, in black characters) and also in the presence of DMSO (1%, in gray characters). The addition of DMSO negatively affected the channel inhibitory capacity of the water-soluble linear and monocyclic peptides. To allow comparison, the inhibitory effects of **SBL-PX1-195** were compared with the ones of ^10^Panx1 and the two stapled **SBL-PX1-42** and **SBL-PX1-44**, all being dissolved in a saline solution containing DMSO (1%). Interestingly, compound **SBL-PX1-195** inhibited HOS-induced ATP release by 48% at 100 μM, thereby improving the inhibitory capacity of ATP release of compounds **SBL-PX1-42** and **SBL-PX1-44** from which it was derived (Figure 7A). Importantly, the channel inhibitory capacity of **SBL-PX1-195** was similar when used at 12.5 μM. Finally, the double-stapled sequence **SBL-PX1-195** was not cytotoxic despite the use of a minimal amount of DMSO (Supporting Information, Figure S7).

Because of the limited solubility of **SBL-PX1-195** and the preference to avoid the use of DMSO in the future *in vivo* evaluation of the peptidomimetics, a “solubilizing tail” composed of one lysine,^53^ linked to the peptide *via* an alkyl spacer [herein a 6-aminohexanoic acid (Ahx) residue], was designed to overcome solubility issues (**SBL-PX1-206**, Figure 6B). Of note, the double triazole linker compound maintained a high helical content as an α-helicity equivalent to the monostapled peptides **SBL-PX1-42** and **SBL-PX1-44** was noted (*i.e.*, 44% based on the mean residue ellipticity at 222 nm, Supporting Information, Figure S21) and also supported by our MD simulations (Supporting Information, Figures S9 and S10). Additionally, the *in vitro* proteolytic stability of the resulting water-soluble double-stapled compound **SBL-PX1-206** was assessed. Interestingly, after 24 h, around 20% of bicyclic **SBL-PX1-206** was still intact in the blood plasma (Figure 6C). The only identified metabolite present in the solution (around 80%, Figure 6B,C) was the active bicyclic segment in which the lysine of the solubilizing tail was cleaved, confirming thereby that amide bonds within both cycles were protected against proteolytic degradation.

Activation of endothelial cells plays a crucial role in acute and chronic cardiovascular inflammation as these cells secrete proinflammatory cytokines (*e.g.*, IL-1β) and allow for adhesion and transmigration of (circulating) leukocytes over the endothelial barrier into the affected tissue.^80,81^ As Panx1 channel activity is known to regulate IL-1β secretion and leukocyte transmigration processes,^4^ the inhibitory capacity of our most promising ^10^Panx1-based analogues (*i.e.*, **SBL-PX1-42**, **SBL-PX1-44**, and **SBL-PX1-206**) was also investigated in endothelial cells. The endothelial cell line EA.hy926 derived from primary human umbilical cord endothelial cells (HUVECs) was found to express relatively stable levels of Panx1 (Figure 7B–D). Importantly, the fully glycosylated cell membrane-bound form of Panx1 was found in these cells by Western blotting and immunostaining (Figure 7C,D), thus allowing for functional studies of Panx1 channels.

Similar to B16-BL6 cells, a 2–3-fold enhancement of ATP release from EA.hy926 cells was observed upon HOS stimulation (Figure 7E). However, the absolute levels of ATP release were lower in these endothelial cells (around 300 *vs* 500–600 nM in B16-BL6 cells). As expected, Pbn reduced HOS-induced ATP release from EA.hy926 cells, albeit by a lower fraction than the one observed in B16-BL6 cells (Figure 7E *vs*7A). The reason for this different efficacy of Pbn is unknown but may be due to its nonspecific action on other ATP release channels or transporters,^68^ of which the expression naturally varies among different cell types. When used at 100 μM, ^10^Panx1 tended to decrease ATP release from EA.hy926 cells (Figure 7E, left), an effect that increased when used at 12.5 μM to a comparable level (around 35%) than the inhibition of ATP release seen with Pbn (Figure 7E, right). These results hint at a U-shaped concentration–response curve for ^10^Panx1’s efficacy to inhibit ATP release from endothelial cells and are comparable to our earlier observations in B16-BL6 cells (*cf.*Figure 3F). Both macrocyclic compounds **SBL-PX1-42** and **SBL-PX1-44** were able to inhibit HOS-induced ATP release from EA.hy926 cells at both concentrations used (100 and 12.5 μM). For unknown reasons, **SBL-PX1-44** was slightly more potent in this cell line than **SBL-PX1-42**, but overall, these data are in line with our earlier observations in B16-BL6 cells. The water-soluble double-stapled ^10^Panx1 peptidomimetic **SBL-PX1-206** reduced HOS-induced ATP release from EA.hy926 cells by about 25% at 12.5 μM. Interestingly, while the Panx1 channel inhibitory efficacies of **SBL-PX1-42** and **SBL-PX1-44** reached its maximum at 100 μM in EA.hy926 cells, the efficacy of **SBL-PX1-206** slightly improved when further lowering the concentration used to 12.5 μM. Noteworthy, the anchored cationic tail responsible for enhanced solubility does not interfere with *in vitro* ATP release experiments. In fact, when this moiety has been added to the linear ^10^Panx1 peptide sequence (*i.e.*, **SBL-PX1-214**), a similar bioactivity as the control reference peptide has been observed (data not shown). Stapled and double-stapled ^10^Panx1 analogues were also not cytotoxic when tested at 100 and 12.5 μM in EA.hy926 cells (Supporting Information, Figure S8). Altogether, these results on endothelial cells confirmed that the introduction of two macrocycles within the peptide backbone might be a good strategy for Panx1 channel inhibition at low concentrations.

### Assessment of Anti-Inflammatory Properties of the Double-Stapled ^10^Panx1 Analogue

**Figure 8:**
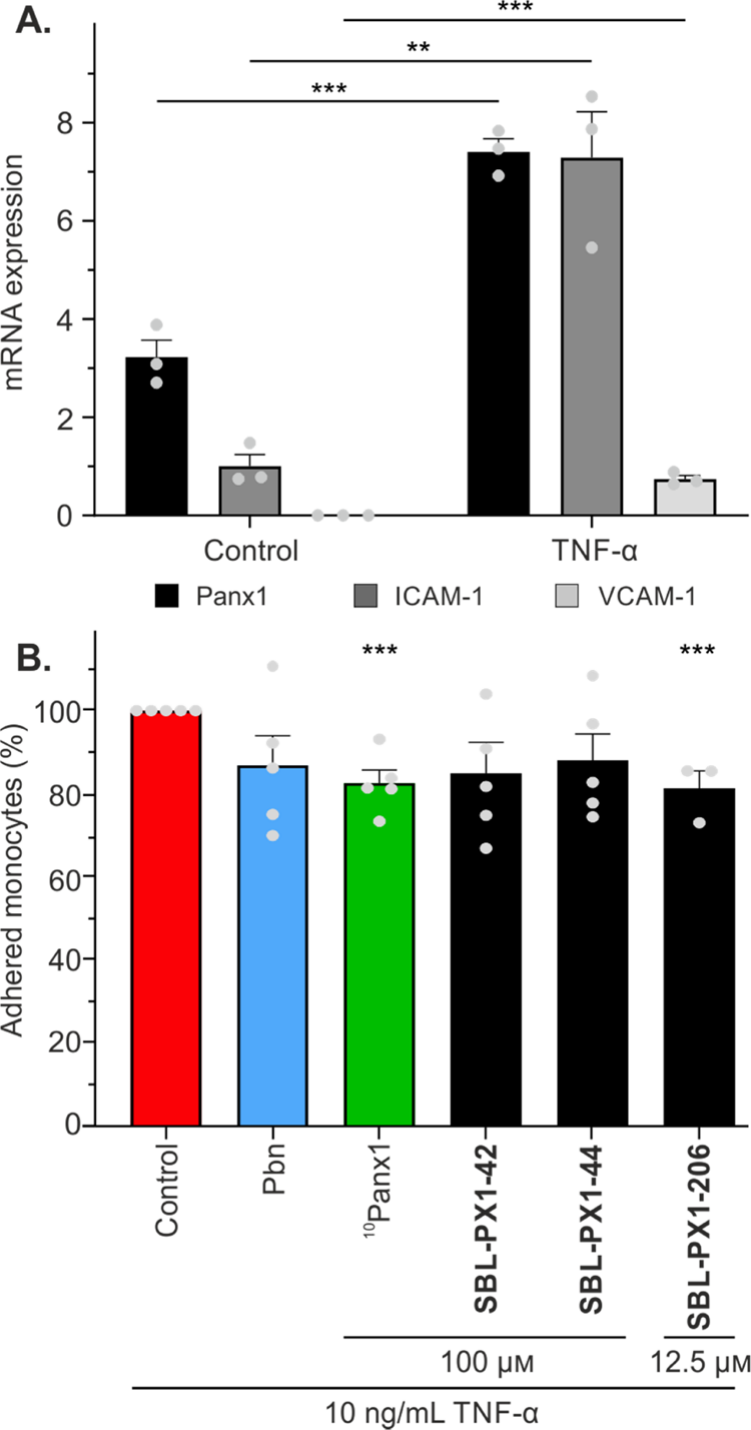
Anti-inflammatory effects of **SBL-PX1-42**, **SBL-PX1-44**, and **SBL-PX1-206**. (A) Panx1, ICAM-1, and VCAM-1 mRNA expression in EA.hy926 cells in control conditions or after 24 h stimulation with 10 ng/mL TNF-α. (B) Percentage of THP-1 monocyte adhesion to an EA.hy926 cell monolayer after 24 h TNF-α stimulation and 2 h coincubation. ^10^Panx1 and stapled ^10^Panx1 analogues were used at 100 μM while **SBL-PX1-206** was used at 12.5 μM. Control conditions correspond to TNF-α-activated endothelial cells. Pbn (2.5 mM) was used as a reference compound. Gray dots represent individual experiments. Data are shown as the mean ± SEM *P value compared to the control conditions.

The anti-inflammatory potential of the water-soluble double-stapled ^10^Panx1 analogue **SBL-PX1-206** was evaluated in assays testing the adhesion of THP-1 monocytes, known to express Panx1 (Figure 7B),^22^ to endothelial cells. Briefly, monolayers of EA.hy926 cells were stimulated with 10 ng/mL of tumor necrosis factor α (TNF-α) for 24 h to induce their activation, making them prone to monocyte adhesion. As expected, endothelial activation with TNF-α enhanced the expression of the adhesion molecules VCAM-1 and ICAM-1 as well as of Panx1 (Figure 8A). EA.hy926 cells and THP-1 monocytes were then incubated for 15 min with **SBL-PX1-206**, **SBL-PX1-42**, or **SBL-PX1-44** at the concentration inducing the maximal inhibitory effect on ATP release from EA.hy926 cells (*cf*. Figure 7E). Subsequently, monocytes were allowed to adhere to the endothelial cells for 2 h. As expected, ^10^Panx1 inhibited monocyte adhesion by 17% (Figure 8B). Although **SBL-PX1-42** and **SBL-PX1-44** also tended to inhibit monocyte adhesion when used at 100 μM, this level of Panx1 channel inhibition was already reached when using only 12.5 μM of **SBL-PX1-206**. By being equipotent to Pbn, a compound that has already been shown to display beneficial action in the prevention of cardiovascular inflammatory disease in mice,^82^ by inhibiting monocyte adhesion to endothelial cells, these experiments identify **SBL-PX1-206** as a promising peptidomimetic for further *in vivo* studies.

## Conclusions

The introduction of rigidity by means of macrocyclization within the ^10^Panx1 peptide backbone further improved the bioactivity of the most established reference peptide inhibitor of Panx1 channels. Indeed, the inhibitory capacity of macrocyclic ^10^Panx1-based compounds **SBL-PX1-42** and **SBL-PX1-44** was higher compared with the linear ^10^Panx1 peptide without being cytotoxic. Although the increase in inhibitory capacity was modest (two-fold at 30 min in saline solutions), the Panx1 channel inhibitory effect was observed in two different complementary *in vitro* assays (*i.e.*, ATP release and Yo-Pro-1 uptake assays), demonstrating bidirectional inhibitory effects on Panx1 channel function. Most importantly, the stability of **SBL-PX1-42** and **SBL-PX1-44** in human plasma was increased by 30-fold so that a longer-lasting effect of Panx1 channel inhibition may be expected in pathophysiological applications. Strikingly, the two above-mentioned lead stapled peptidomimetics corresponded to the sequences with the highest helical content according to CD studies (*i.e.*, 56 and 44%, respectively). The robustness of stapling toward proteolytic degradation of amide bonds located between the stapling side chains of the above-mentioned lead peptidomimetics has led to the synthesis of a unique “double-stapled” structure, and two triazole linkers have been introduced in the sequence by combining the same tether positions present in compounds **SBL-PX1-42** and **SBL-PX1–44**, leading to compound **SBL-PX1-206**. The single and double-stapled peptidomimetics not only reproducibly inhibited Panx1 channels in a tumor cell line with a high level of Panx1 expression but also efficiently inhibited these channels in a natural target during inflammation, *i.e.*, the endothelium, with a lower and more variable Panx1 expression level. Targeting Panx1 channels with our lead double-stapled peptidomimetic **SBL-PX1-206** was shown to inhibit monocyte adhesion to the endothelial monolayer by about 20%. Importantly, this significant reduction of the mandatory first step in the inflammatory cascade was obtained by using a low concentration (12.5 μM) of the double-stapled peptidomimetic. Nonetheless, it is expected that further structure-based efforts will still allow optimization of the compounds’ activity. Future *in vivo* targeting of Panx1 channels is particularly attractive for cardiovascular inflammatory maladies as their beneficial effects may extend beyond endothelial adhesion of inflammatory cells and might also involve effects on the P2X7-Panx1-NLRP3 signaling axis, chemotaxis, apoptotic clearance of cells, T cell responses, and fibroblast activation, this way affecting both the initiation and resolution of disease.^83^

## Experimental Section

### Materials and Methods for Chemistry

All natural amino acids (Fmoc-protected), resins (Rink Amide AM resins and preloaded Fmoc-Tyr^(*t*Bu)^ and Fmoc-Ala-Wang resins), and coupling reagent *O*-(benzotriazol-1-yl)-*N*,*N*,*N*′,*N*′-tetramethyluronium hexafluorophosphate (HBTU) were purchased from Chem-Impex. The unnatural propargylglycine (l- and d-Pra), homo-propargylglycine (hPra), azido-lysine (Azk), azido-ornithine (Azo), and amino acids (Fmoc-protected) were purchased from Chem-Impex. Copper(I) bromide and sodium ascorbate were obtained from Fluorochem and Sigma-Aldrich, respectively. Reagents 4-methylpiperidine, diisopropylethylamine (DIPEA), dimethyl sulfoxide (DMSO), and pyridine hydrochloride were bought from Sigma-Aldrich. Trifluoroacetic acid (TFA), triisopropylsilane (TIS), ethyl cyano(hydroxyimino)acetate (Oxyma), *N*,*N*′-diisopropylcarbodiimide (DIC), and 2,2,2-trifluoroethanol (TFE) were purchased from Fluorochem. Phosphate-buffered saline (PBS) tablets were purchased from Sigma-Aldrich. Solvents like dichloromethane (DCM, analytical grade), acetonitrile (ACN, high-performance liquid chromatography (HPLC) gradient grade), and diethyl ether (Et_2_O, analytical grade) were purchased from Sigma-Aldrich while *N*,*N*-dimethylformamide (DMF, 99.5%) and methanol (MeOH, HPLC gradient grade) were purchased from ACROS Organics. The Milli-Q water was obtained after purification through a Millipore Simplicity UV system. Analytical reversed-phase-HPLC (RP-HPLC) was performed on a VWR-Hitachi Chromaster HPLC with a Chromolith High-Resolution RP-18C column from Merck (150 mm × 4.6 mm, 1.1 μm, 150 Å). The flow rate was 3 mL/min, and ultraviolet (UV) detection occurred at 214 nm. The solvent system used consisted of 0.1% TFA in ultrapure water (A) and 0.1% TFA in acetonitrile (B) with a gradient from 3 to 100% B over a 6 min runtime. For liquid chromatography–mass spectrometry (LC/MS) analysis, the HPLC unit used was a Waters 600 system combined with a Waters 2487 UV detector at 215 nm, and as a stationary phase, an EC 150/2 NUCLEODUR 300–5 C18 column (150 mm × 2.1 mm, 3 μm, 300 Å). The solvent system used was 0.1% formic acid in water (A) and 0.1% formic acid in acetonitrile (B), with a gradient going from 3 to 100% B over 20 min with a flow rate of 0.3 mL/min. The MS unit, coupled with the HPLC system, was a Micromass QTOF-microsystem. For high-resolution mass spectroscopy, the same MS system was used with a reserpine (2.10–3 mg/mL) solution in H_2_O/ACN (1:1) as a reference. Semipreparative RP-HPLC-purifications were done using a Gilson HPLC system with a Gilson 322 pump equipped with an INTERCHIM Vydac 150HC C18 column (250 mm × 22 mm, 10 μm) and a Waters UV/vis-156 detector at 215 nm. The same solvent system was used as that applied for the analytical RP-HPLC with a flow rate of 20 mL/min.

#### Peptide Synthesis

Linear **H1** peptide and ^10^Panx1 reference peptide were synthesized from a preloaded Tyr^(*t*Bu)^-Wang resin (loading 0.45 mmol g^–1^) and an Ala-Wang resin (loading 0.68 mmol g^–1^), respectively, using the automatic synthesizer (CEM Liberty Blue). Couplings were performed with Fmoc-protected amino acid (5 equiv), stock solutions of DIC (0.5 M), and Oxyma (1 M) in DMF. At the end of the synthesis, the resin was removed from the synthesizer and washed several times with DCM. Cyclic ^10^Panx1 analogues were synthesized on a 0.1 mmol scale manually using the Fmoc strategy on Rink amide resin (loading 0.13–0.6 mmol g^–1^). During the manual synthesis, the resin was first swollen in DMF for 20 min, followed by a double Fmoc deprotection using a solution of 4-methylpiperidine in DMF (20%, vol/vol) for 5 and 15 min, respectively, before being subsequently washed with DMF and DCM. Then, a solution of Fmoc-protected amino acid (3 equiv and 1.5 equiv for natural and unnatural amino acids, respectively) activated by HBTU (3 equiv) and DIPEA (5 equiv) in DMF was added to the resin. The reaction was wiggled for approx. 1 h. After each coupling, the mixture was filtered off, and the resin was washed several times with DMF and DCM. Before every coupling, the resin was treated with the Fmoc-deprotection solution. The full linear sequences were built by repetition of the above steps. The *N*-terminal acetylation was performed using a solution of acetic anhydride (10 equiv) and DIPEA (5 equiv) in DCM for 1 h.

#### Peptide Cyclization

Cu(I)-catalyzed azide–alkyne cycloaddition (CuAAC) was carried out on resin-bound linear precursor peptides. First, the resin was swollen in DMF, filtered, and washed with degassed DMF. Then, copper(I)bromide (3 equiv) in DMF, sodium ascorbate (3 equiv) dissolved in water (3 mL mmol^–1^), 2,6-lutidine (10 equiv), and DIPEA (10 equiv) were added. After bubbling argon through the mixture for several minutes, the syringe was closed and the reaction mixture was stirred for 24 h at room temperature. The mixture was filtered off, and the resin was washed several times with a solution of pyridine hydrochloride dissolved in a mixture of DCM/MeOH (1 M, 95:5, vol/vol), followed by washing steps with DMF and DCM. Successful cyclization was verified by performing a small-scale cleavage followed by HPLC and LC-MS analyses, which indicated a shift in retention times compared to the linear starting materials.

#### Peptide Cleavage and Purification

After completion of the sequences, peptides were deprotected and cleaved from the resin by treatment with a cocktail solution constituted of TFA/TIS/H_2_O (95:2.5:2.5) for 2 h at room temperature. The resulting cleavage mixtures were evaporated, and the crude peptide was precipitated with cold Et_2_O. After centrifugation, the precipitated peptide was dissolved in a mixture of H_2_O/ACN (1:1) and lyophilized. Finally, the crude peptide was purified by preparative reversed-phase HPLC (RP-HPLC) and the pure fractions were lyophilized. Peptides were obtained as a powder (as TFA salts) and were characterized by high-resolution mass spectroscopy (HRMS). The purity of all peptides was found to be ≥96%, according to HPLC analysis.

### Methods for CD Spectroscopy

Spectra were measured with a JASCO J-715 CD spectropolarimeter equipped with a temperature controller using a 1 mm path length quartz cuvette and a scan rate of 5 nm min^–1^. The spectra were recorded in the wavelength range of 190–260 nm with a resolution and a bandwidth of 0.5 and 1.0 nm, respectively. The sensitivity and scan rate of the spectrometer were set to 100 mdeg and 50 nm min^–1^, respectively. Samples were prepared in phosphate-buffered saline (PBS, 10 mM phosphate, 137 mM sodium chloride, 2.7 mM potassium chloride, pH 7.4, at room temperature) to the final concentration of 100 μM with or without 2,2,2-trifluoroethanol (TFE). CD spectra were averaged over 3–5 scans with the baseline subtracted from analogous conditions as those for the samples and converted to the mean residue ellipticity. Helicity was calculated as previously reported (see the Supporting Information).^52^

### Methods for *In Vitro* Experiments

#### Cell Lines

Mouse melanoma B16-BL6 cells, kindly provided by Dr. Lubor Borsig (Institute of Physiology, University of Zürich, Switzerland),^84^ rat H9c2 cardiomyoblasts (ATCC no CRL-1446), and human vascular endothelial EA.hy926 cells (ATCC no CRL-2922) were grown in Dulbecco’s modified Eagle’s medium (DMEM; Gibco), d-glucose (25 mM), sodium pyruvate (1 mM), l-glutamine (4 mM) supplemented with fetal bovine serum (FBS, 10%, vol/vol), and penicillin/streptomycin (P/S, 1%, vol/vol) at 37 °C in 5% CO_2_. B16-BL6 and H9c2 cells were passaged every 2 days by incubation with Trypsin-EDTA (2 min at 37 °C) and centrifugation for 5 min (1200 rpm at room temperature). Pellets were resuspended in fresh DMEM. H9c2 cells were differentiated toward a cardiac-like phenotype as previously described.^85^ EA.hy926 cells were passaged every 2 days by incubation with PBS-EDTA for 5 min at 37 °C, followed by incubation with Trypsin-EDTA (2 min at 37 °C) and centrifugation for 5 min (1200 rpm at room temperature). Pellets were resuspended in fresh DMEM and plated in culture dishes precoated with 1.5% gelatin.

Human monocytic THP-1 cells (ATCC no. TIB-202) were grown in Roswell Park Memorial Institute (RMPI) medium with GlutaMAX (Gibco) supplemented with FBS (10%, vol/vol) and P/S (1%, vol/vol) at 37 °C in 5% CO_2_. Cells were passaged by centrifugation for 5 min (1200 rpm at room temperature) every 2 days, and pellets were resuspended in fresh RPMI medium with GlutaMAX.

#### Real-Time Quantitative Polymerase Chain Reaction (qPCR)

Total RNA was extracted from murine, human, rat tissues, and cell lines using the NucleoSpin RNA kit (Macherey-Nagel). The reverse transcription was realized using the Quantitect Reverse Transcription kit (Qiagen). Real-time quantitative PCR was performed using TaqMan Fast Universal master mix (Applied Biosystem) and the ABI Prism StepOnePlus Sequence Detection System (Applied Biosystems). Mouse, human, and rat Panx1, Panx2, and Panx3, human vascular cell adhesion molecule-1 (VCAM-1), intercellular adhesion molecule-1 (ICAM-1), as well as human and mouse ribosomal 18S and rat glyceraldehyde-3-phosphate dehydrogenase (GAPDH) probes and primers, were obtained from Applied Biosystems. Gene expression was normalized to corresponding GAPDH or 18S expression. Triplicates were performed for each independent experiment.

#### Western Blotting

Proteins from cell lines and tissues were extracted in RIPA lysis buffer [NP40, 1%; NaCl, 30 mM; Tris, 50 mM (pH 8.0); NaF, 10 mM; Na_3_VO_4_, 2 mM; PMSF, 1 mM; EDTA, 1 mM (pH 7.4); SDS, 0.05%; sodium-deoxycholate, 0.2%; supplemented with a protease inhibitor cocktail]. Protein concentration was measured by means of a bicinchoninic acid assay (BCA; Thermo Fisher Scientific) according to the manufacturer’s instructions. Panx1 was detected on a PVDC membrane using a rabbit anti-Panx1 primary antibody diluted at 1/1000 (Cell Signaling) and a secondary antirabbit antibody diluted at 1/5000 (Jackson Laboratories). A chemiluminescence signal was detected by the Immobilon ECL Ultra Western HRP Substrate (Millipore) using a LAS 4000 Fujifilm. GAPDH was used as a loading control. A sample of human platelets has been used as a positive control for Panx1 expression in EA.hy926 cells. Platelets were extracted as previously described.^86^

#### Immunofluorescence Staining

EA.hy926 cells were stained with anti-Panx1 HRB462 mini-body (Geneva Antibody Facility) diluted at 1/250 as previously described.^87^ H9c2 cells were stained with custom-made anti-mPanx1_414–425_ diluted at 1/500 using previously established protocols.^62^ Briefly, cells were fixed with PFA (4%) for 15 min and permeabilized in Triton X-100 (0.3%; vol/vol) for 15 min. Cells were then incubated in NH_4_Cl (0.5 M) for 15 min and blocked with BSA solution (2%) for 45 min. Staining was performed overnight at 4 °C, and cells were thereafter stained with a secondary goat anti-chicken antibody diluted at 1/500 (Jackson Laboratories). Cells were counterstained with Evans blue (0.003%). Nuclei were stained with DAPI at 1/20,000 for 10 min, and slides were mounted in Vectashield antifade mounting medium (VECTOR Laboratories). Images were captured using a Zeiss Axio Imager Z1 with an EC Plan-Neofluar 40×/1.3 Oil (42,462.9900) objective.

#### ATP Release Assay

ATP release assays were performed using previously described methods.^62^ Briefly, B16-BL6, EA.hy926, and H9c2 cells were seeded on 96-well plates and grown to confluence. Cells were washed with Tyrode buffer [NaCl, 124 mM; KCl, 2.44 mM; NaHCO_3_, 10.82 mM; NaH_2_PO_4_, 0.38 mM; MgCl_2_, 0.91 mM; CaCl_2_, 1.82 mM, (pH 7.35; 295 mOsm/kg)] for 5 min at room temperature. After washing, cells were preincubated for 10 min in Tyrode buffer containing probenecid (Pbn; 2.5 mM), ^10^Panx1 peptides (Tocris or custom-made), or ^10^Panx1 analogues at various concentrations ranging from 400 μM to 10 nM. Cells were then incubated with the aforementioned compounds in a hypo-osmotic shock (HOS) solution [NaCl, 30.24 mM; KCl, 10 mM; NaHCO_3_, 10.82 mM; NaH_2_PO_4_, 0.38 mM; MgCl_2_, 0.91 mM; CaCl_2_, 1.82 mM (pH 7.35; 136 mOsm/kg)] for 30 min. Supernatants were collected, and ATP bioluminescence measurements were performed with an ATP bioluminescence kit (Sigma-Aldrich) according to the manufacturer’s instructions. ATP release in each condition was expressed as the molar concentration or as a percentage of HOS-induced ATP release. ATP release of cells incubated with Tyrode was used as a control for basal ATP release. Triplicates were performed for each independent experiment.

#### Yo-Pro-1 Dye Uptake Assay

B16-BL6 cells were plated on 96-well plates and grown until confluency. Cells were washed with Tyrode buffer and preincubated for 10 min with Pbn (2.5 mM) or with ^10^Panx1 peptides (Tocris or custom-made) or ^10^Panx1 analogues (100 and 12.5 μM). Cells were then incubated in the HOS solution containing the aforementioned compounds. Yo-Pro-1 (5 μM, Life Technologies) was added to the cells, and fluorescence was continuously recorded for 30 min with FDSS/μCELL Functional Drug Screening System (Hamamatsu Photonics). Yo-Pro-1 uptake in each condition was expressed as a percentage of HOS-induced Yo-Pro-1 uptake. Yo-Pro-1 uptake of cells incubated with Tyrode was used as a control for basal Yo-Pro-1 uptake. Triplicates were performed for each independent experiment.

#### Lactate Dehydrogenase (LDH) Assay

Potential cytotoxic effects of the ^10^Panx1 analogues at concentrations ranging from 100 to 1.56 μM were assessed in B16-BL6 and EA.hy926 cells (after 30 min incubation) by measuring LDH in cell supernatants using an LDH activity assay (Roche) according to the manufacturer’s instructions. Triplicates were performed for each independent experiment.

#### Monocyte Adhesion Assay

EA.hy926 cells (150,000/well) were seeded in 24-well plates and grown until reaching a confluent monolayer. Cells were stimulated for 24 h with 10 ng/mL of human tumor necrosis factor α (hTNF- α; R&D Biosystems) at 37 °C in 5% CO_2_. The proinflammatory effects of hTNF-α were verified by induction of adhesion molecule expression (VCAM-1 and ICAM-1). THP-1 monocytes were labeled with 5–6-carboxyfluorescein diacetate succinimidyl ester (CFDA-SE, 5 μM; Thermo Fisher Scientific) using previously established protocols.^88^ EA.hy926 and fluorescently labeled THP-1 cells were incubated for 15 min with 2.5 mM Pbn or with ^10^Panx1 peptides (Tocris or custom-made) or ^10^Panx1 analogues (100 and 12.5 μM) in RPMI medium with GlutaMAX without FBS. Fluorescently labeled THP-1 cells (50,000/well) were then added to EA.hy926 cell monolayers and allowed to adhere for 2 h at 37 °C in an incubator (5% CO_2_). Thereafter, photographs were captured using a ZOE Fluorescent Cell Imager (Biorad). Monocyte adhesion was quantified by measuring the average green fluorescence signal of 4 microscopy fields in each well and expressed as a percentage of the control condition (TNF-α activated EA.hy926 cells). Duplicates were performed for each independent experiment.

#### Statistical Analysis

Statistical analyses were performed by using GraphPad Prism software. Student’s *t*-tests were performed for statistical comparisons. Results are shown as mean ± SEM. Statistical significance is indicated by *P ≤ 0.05, **P ≤ 0.01, and ***P ≤ 0.001.

### Methods for *In Vitro* Plasma Stability

The proteolytic stability of the peptides was determined through *in vitro* stability assays in human plasma, as previously reported.^31,89^ Briefly, a stock solution of the peptide was prepared at a concentration of 1–2 mm in Milli-Q water and was used for further dilutions. Prior to the actual stability testing, the selectivity of the assay and stability of the working solutions were evaluated in parallel with an assessment of the linearity, accuracy, and precision of the method using three separately prepared calibration curves. The samples were analyzed by RP-HPLC on an Agilent 1200 series gradient HPLC system in combination with an EC HPLC column EC 150/2 NUCLEODUR (C18 HTec, 3 μm length: 150 mm, ID: 2 mm. Macherey-Nagel). The half-lives of the peptides were calculated by interpolating the data based on the obtained calibration curve. Afterward, the log concentrations as a function of time were transferred to a semi-log chart. Metabolites were identified by reanalyzing the samples using LC-MS on the same system previously described.^31^

### Methods for Molecular Modeling

A preliminary peptidomimetic *de novo* three-dimensional (3D) structure prediction was performed with the Pepfold3 web server^90^ by using **H1** and ^10^Panx1 sequences as input of the algorithm. After this step, four different peptidomimetics modifications were introduced using YASARA^91^ editing features, and 50 ns molecular dynamics simulations were performed using the AMBER14 force field^92^ within YASARA. The parameters used for the simulations were the following: pH, 7.4; ion concentration, 0.9% of NaCl; temperature, 298 K; and periodic borders. At every step of the simulations, the peptidomimetic percentage of different 3D structures was determined with a specific YASARA macro designed for this purpose. The results of these calculations are obtained as “.sim” files, and for the following analysis, PDB structures were saved every 0.1 ns (therefore, obtaining 500 PDB structures of each peptidomimetic simulation). The hydrogen bond prediction analysis of the first and last steps of the simulations was performed using YASARA software. Additionally, YASARA functionalities were used for visualization of the peptides throughout the MD simulations, allowing for a detailed examination of their 3D structures and interactions.

## Supporting Information Available

Heptameric assembly of Panx1; helix-wheel diagram of the α-helix found in EL1; biological data including *in vitro* inhibition; channel specificity and cytotoxic effect of the tested analogues; MD data of reported analogues; CD spectroscopy of reported analogues; and peptide characterization including HPLC and HRMS data for described analogues (PDF)
Molecular formula strings (CSV)

## Abbreviations

ACN: acetonitrile
ATP: adenosine-5′-triphosphate
Azk: azido-lysine
Ahx: 6-aminohexanoic acid
Azo: azido-ornithine
BSA: bovine serum albumin
CuAAC: copper(I)-catalyzed azide–alkyne cycloaddition
DCM: dichloromethane
DIC: *N*,*N*′-diisopropylcarbodiimide
DIPEA: diisopropylethylamine
DMEM: Dulbecco’s modified Eagle’s medium
DMF: *N*,*N*-dimethylformamide
DMSO: dimethyl sulfoxide
EC: endothelial cells
EDTA: ethylenediaminetetraacetic acid
EL: extracellular loop
Et_2_O: diethyl ether
FBS: fetal bovine serum
Fmoc: 9-fluorenylmethyloxycarbonyl
HBTU: *N*,*N*,*N*′,*N*′-tetramethyl-*O*-(1*H*-benzotriazol-1-yl)uronium hexafluorophosphate
HOBt: 1-hydroxybenzo-triazole
HOS: hypo-osmotic shock
HPLC: high-performance liquid chromatography
hPra: homo-propargylglycine
HRMS: high-resolution mass spectroscopy
ICAM-1: intercellular adhesion molecule-1
LC–MS: liquid chromatography–mass spectrometry
LDH: lactate dehydrogenase
MeOH: methanol
MD: molecular dynamic
Panx: pannexin
PBS: phosphate-buffered saline
Pbn: probenecid
PMSF: phenylmethanesulfonyl fluoride
Pra: propargylglycine
qPCR: real-time quantitative polymerase chain reaction
oxyma: ethyl cyano(hydroxyimino)acetate
RP-HPLC: reverse-phase high-performance liquid chromatography
SAR: structure–activity relationship
SD: standard deviation
SDS: sodium dodecyl sulfate
SEM: standard error of the mean
SPPS: solid-phase peptide synthesis
TFA: trifluoroacetic acid
TFE: 2,2,2-trifluoroethanol
TIS: triisopropylsilane
TM: transmembrane domain
UV: ultraviolet
VCAM-1: human vascular cell adhesion molecule-1
