# Supplementary material for: Structure-Based Design and Synthesis of Stapled 10Panx1 Analogues for Use in Cardiovascular Inflammatory Diseases
Source: J Med Chem. 2023 Sep 13;66(18):13086–102. doi: 10.1021/acs.jmedchem.3c01116 (PMC10544015; doi:10.1021/acs.jmedchem.3c01116)
Supplement: Supplementary file 1 — jm3c01116_si_001.pdf [file jm3c01116_si_001.pdf]

SUPPORTING INFORMATION

Structure-based design and synthesis of stapled ^{10}Panx1 analogs for use in cardiovascular inflammatory diseases

Arthur Lamouroux,[1,§] Malaury Tournier,[2,§] Debora Iaculli,[1] Anne Caufriez,[1,3] Olga M. Rusiecka,[2] Charlotte Martin,[1] Viviane Bes,[2] Laureano E. Carpio,[4,5] Yana Girardin,[6,7] Remy Loris,[6,7] Andrés Tabernilla,[3] Filippo Molica,[2] Rafael Gozalbes,[4,5] María D. Mayán,[8] Mathieu Vinken,[3] Brenda R. Kwak[2,*] and Steven Ballet[1,*]

[1] Research Group of Organic Chemistry, Departments of chemistry and Bioengineering sciences, Vrije Universiteit Brussel, Pleinlaan 2, B-1050 Brussels, Belgium; [2] Department of Pathology and Immunology and Geneva Center for Inflammation Research, Faculty of Medicine, University of Geneva, Rue Michel-Servet 1, CH-1211 Geneva, Switzerland; [3] Research unit of *In Vitro* Toxicology and Dermato-cosmetology, Department of Pharmaceutical sciences, Vrije Universiteit Brussel, Laarbeeklaan 103, 1090 Brussels, Belgium; [4] ProtoQSAR SL, Centro Europeo de Empresas Innovadoras, Parque Tecnológico de Valencia, Avda. Benjamin Franklin 12, 46980 Paterna, Spain; [5] MolDrug AI Systems SL, c/Olimpia Arozena 45, 46018 Valencia, Spain; [6] Structural Biology Brussels, Department of Biotechnology, Vrije Universiteit Brussel, Pleinlaan 2, B-1050 Brussels, Belgium; [7] Centre for Structural Biology, VIB, Pleinlaan 2, 1050 Brussels, Belgium; [8] CellCOM Research Group, Instituto de Investigación Biomédica de A Coruña, Servizo Galego de Saúde, Universidade da Coruña, 15071 A Coruña, Spain.

[§] shared first

* shared last and corresponding authors: Brenda R. Kwak, email: brenda.kwakchanson@unige.ch and Steven Ballet – email: steven.ballet@vub.be

This PDF file includes:

I. Supporting figures and tables 2

II. Peptide characterization 13

Characterization 14

RP-HPLC chromatograms of the synthesized peptidomimetics 16

III. Circular Dichroism (CD) spectroscopy 22

Helix content calculation 22

CD spectra of reported peptidomimetics 22

IV. References 27

I. SUPPORTING FIGURES AND TABLES

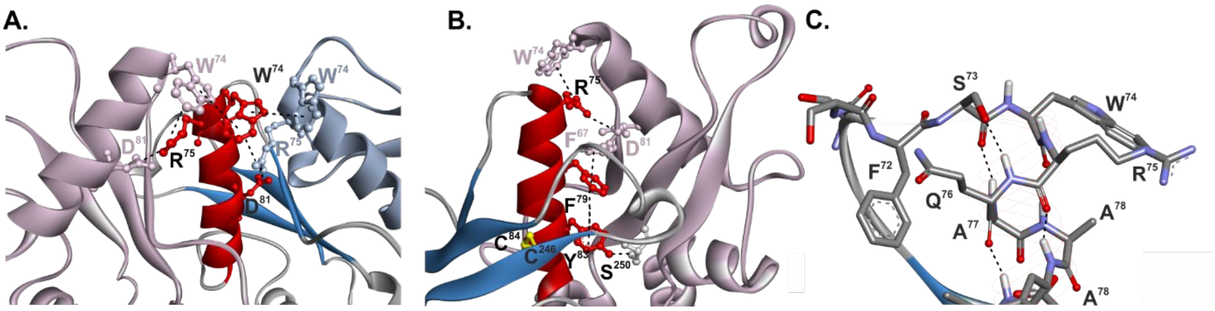

Figure S1. Zooms on the cryo-EM structures of the extracellular loops (EL) of the heptameric assembly of Pannexin1 (PDB 6WBF).[i] A-B) Intra- and inter-subunit interactions between the α-helix **H1** of subunit A – in which α-helices **H1** and β-strands (**S1**–**S3**) are colored in red and blue, respectively – and the adjacent subunits B (in blue) and G (in pink). Residues of interest are represented in ball and stick representation; non-covalent interactions are represented by black dashed lines; disulfide bonds are shown in yellow. C) Intramolecular interactions found in a portion of the extracellular loop of subunit A. For clarity, only residues from F^{72} to A^{78} are in a tube representation. Hydrogen bonding interactions are represented by dashed lines.

Table S1. Intra- and intermolecular interactions of residues belonging to the first extracellular loop with its neighboring subunits (see **Figure 1 and S1**). The subunit of interest A is in black while the adjacent subunits B and G are colored in blue and in pink, respectively. The interactions and distances are based on the Panx1 cryo-EM structure obtained by Ruan *et al.* (PDB 6WBF).[i] Similar results were observed with the cryo-EM structure of Deng *et al.* (PDB 6WBF).

Residue of A[a]	Interacting residue	Interaction[b]	d (Å)	Location of the interactions[b]
$^{A.72}$F	$^{A.80}$V	VDW	3.7	SC-SC
$^{A.73}$S	$^{A.75}$R	$HB_{OH\text{-}NH}$	2.9	SC-BC
	$^{A.76}$Q	$HB_{OH\text{-}NH}$	2.2	SC-BC
	$^{A.77}$A	$HB_{CO\text{-}NH}$	2.0	BB-BB
$^{A.74}$W	$^{A.78}$A	$HB_{CO\text{-}NH}$	1.9	BB-BB
	$^{G.74}$W	π-π	3.1	SC-SC
	$^{B.75}$R	π-+	3.8	SC-SC
	$^{B.74}$W	π-π	3.1	SC-SC
$^{A.75}$R	$^{A.73}$S	$HB_{NH\text{-}OH}$	2.9	BB-SC
	$^{A.79}$F	$HB_{CO\text{-}NH}$	1.8	BB-BB
	$^{G.74}$W	π-+	3.9	SC-SC
	$^{G.81}$D	$HB_{NH\text{-}CO}$	2.0	SC-SC
$^{A.76}$Q	$^{A.73}$S	$HB_{NH\text{-}OH}$	2.2	BB-SC
	$^{A.80}$V	$HB_{CO\text{-}NH}$	1.9	BB-BB
	$^{G.68}$S	$HB_{NH\text{-}CO}$	2.9	SC-BB
$^{A.77}$A	$^{A.73}$S	$HB_{NH\text{-}CO}$	2.0	BB-BB
	$^{A.81}$D	$HB_{CO\text{-}NH}$	1.9	BB-BB
$^{A.78}$A	$^{A.74}$W	$HB_{NH\text{-}CO}$	1.9	BB-BB

	A.82S	HB_{CO-NH}	1.9	BB-BB
A.79F	A.75R	HB_{NH-CO}	1.9	BB-BB
	A.82S	HB_{OH-CO}	2.3	SC-BB
	A.83Y	HB_{CO-HN}	1.8	BB-BB
		VDW	3.4	SC-SC
	G.67F	VDW	3.9	SC-SC
A.80V	A.76Q	HB_{NH-CO}	1.9	BB-BB
	A.84C	HB_{CO-NH}	1.9	BB-BB
	A.72F	VDW	3.6	SC-SC
	A.263F	VDW	3.7	SC-SC
A.81D	A.77A	HB_{NH-CO}	1.9	BB-BB
	A.66C	HB_{CO-NH}	2.7	SC-BB
	A.85W	HB_{CO-NH}	1.9	BB-BB
	B.75R	HB_{CO-NH}	2.0	SC-SC
A.82S	A.78A	HB_{NH-CO}	1.9	BB-BB
		HB_{CO-OH}	2.3	SC-BB
	A.86A	HB_{CO-NH}	1.9	BB-BB
A.83Y	A.79F	HB_{NH-CO}	1.9	BB-BB
		VDW	3.4	SC-SC
	A.87A	HB_{CO-NH}	2.2	BB-BB
	A.250S	HB_{HO-HO}	1.9	SC-SC
A.84C	A.80V	HB_{NH-CO}	1.9	BB-BB
	A.88V	HB_{CO-NH}	2.3	BB-BB
	A.246C	S-S	-	SC-SC
A.85W	A.60I	VDW	3.8	SC-SC
	A.81D	HB_{NH-CO}	1.9	BB-BB
	G.60I	VDW	3.5	SC-SC
A.86A	A.82S	HB_{NH-CO}	1.9	BB-BB
	G.268I	VDW	3.9	SC-SC
A.87A	A.83Y	HB_{NH-CO}	2.2	BB-BB
A.88V	A.84C	HB_{NH-CO}	3.1	BB-BB

[a] Residues highlighted in red belong to the helical sequence **H1**; [b] VDW = van der Waals forces; HB = hydrogen bond; π-+ and π-π stand for π-cation interaction and π-π stacking, respectively; BB = backbone, SC = side chain.

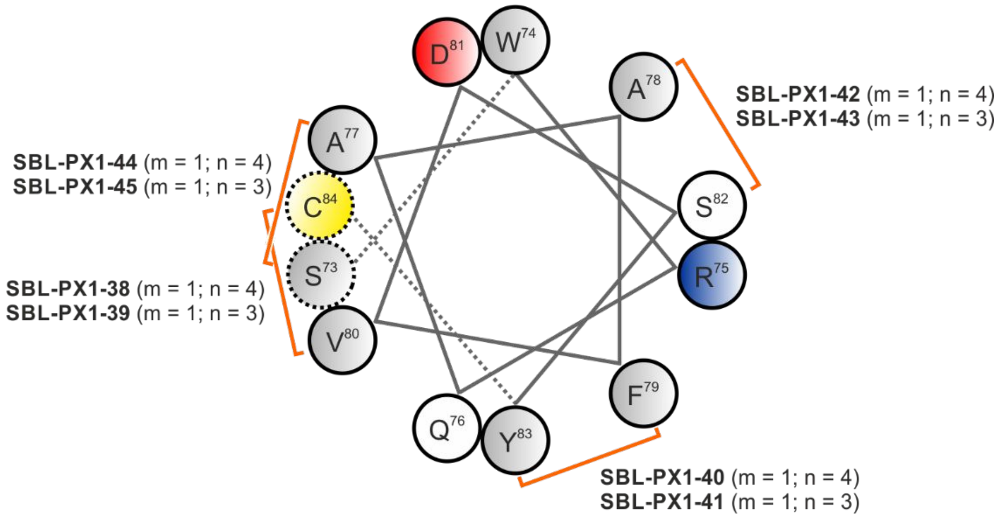

Figure S2. Helical wheel representation of a portion of the α-helix **H1** found in EL1. Polar, hydrophobic, positively and negatively charged amino acids are represented by white, grey, blue and red spheres, respectively. Residues belonging to ^{10}Panx1 sequence (^{74}WRQAAFVDSY^{83}) are encircled with full lines. Orange squares indicate the *(i,i+4)* stapled amino acid positions that have been used for the cyclization screening (see **Table S2** for peptidomimetic sequences).

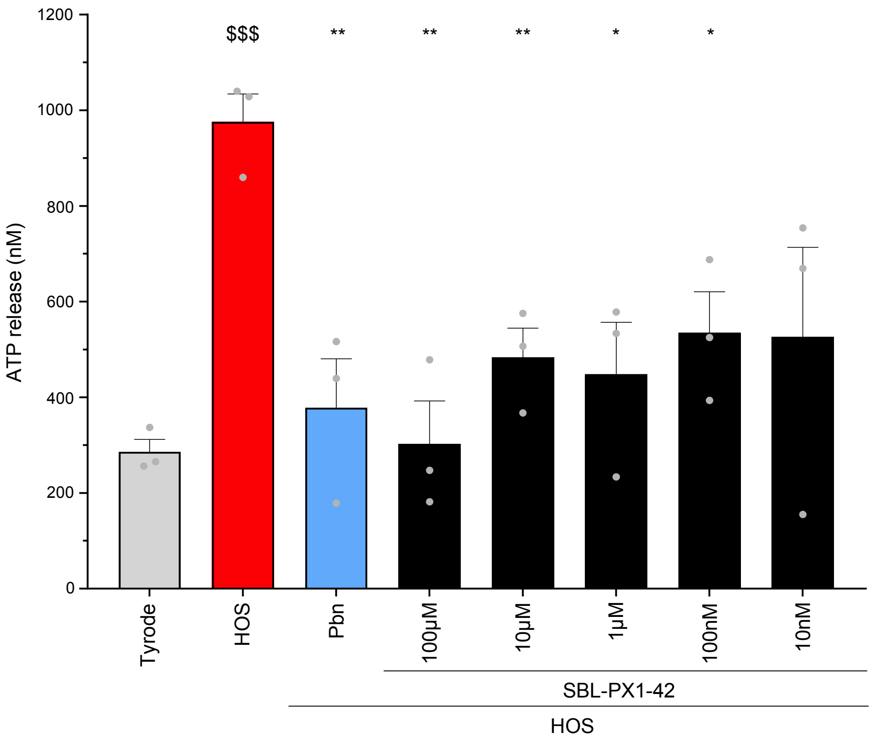

Figure S3. Inhibitory effects of low-dose **SBL-PX1-42** on Panx1 channel-dependent ATP release in B16-BL6 cells. Concentration-response assay of ATP release by B16-BL6 cells with **SBL-PX1-42** at concentrations ranging from 100 µM to 10 nM after 30 minutes HOS stimulation. Tyrode condition represents basal ATP release. HOS induces receptor-independent Panx1 channel opening. The well-known Panx1 channel inhibitor Pbn (2.5 mM) was used as a reference compound. Grey dots represent individual experiments. Data are shown as mean ± SEM. $^{\$}$P value compared to Tyrode condition, *P value compared to HOS condition.

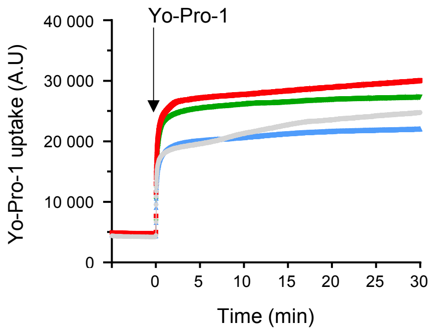

Figure S4. Yo-Pro-1 uptake over time by B16-BL6 cells. Representative curves of Yo-Pro-1 uptake by B16-BL6 cells. Yo-Pro-1 uptake is measured over time during 30 minutes after addition of 5 µM of Yo-Pro-1. Tyrode: basal level (grey), HOS (red), Pbn at 2.5 mM (blue) and ^{10}Panx1 at 100 µM (green).

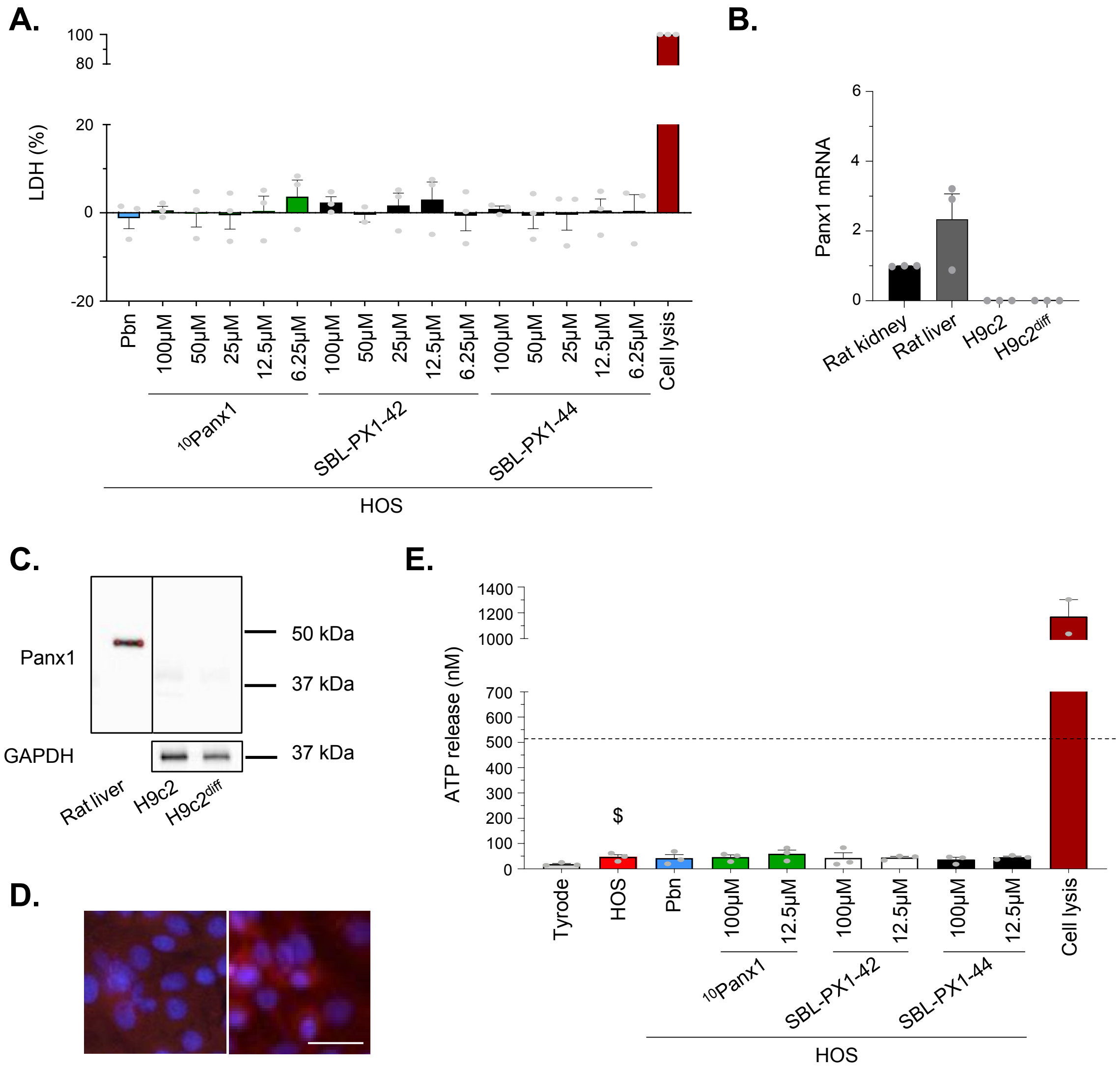

Figure S5. Cytotoxicity and specificity of **SBL-PX1-42** and **SBL-PX1-44**. A) LDH leakage by B16-BL6 cells was used to determine cytotoxicity. ^{10}Panx1 or stapled analogs were used at concentrations ranging from 100 µM to 6.25 µM. LDH leakage was expressed as % of the maximal value induced by cell lysis with 2% Triton X-100. B) Panx1 mRNA levels in undifferentiated and differentiated H9c2 cells. Rat kidney and liver were used as positive controls. C) Panx1 protein expression in

undifferentiated and differentiated H9c2 cells. Rat liver was used as positive control. GAPDH was used as loading control. D) Representative image of immunostaining for Panx1 in H9c2 cells (*left*). Negative control was performed by omission of primary antibody (*right*). Cells and nuclei were counterstained with Evans Blue (in red) and DAPI (in blue), respectively. Scale bar = 50 µM. E) ATP release by H9c2 cells with stapled ^{10}Panx1 analogs after 30 minutes HOS stimulation. Cells were incubated with 100 µM or 12.5 µM of ^{10}Panx1 and stapled analogs. Cells were lysed with 2% Triton X-100 to determine the maximal ATP content. Tyrode condition represents basal ATP release. HOS induces receptor-independent Panx1 channel opening. The well-known Panx1 channel inhibitor Pbn (2.5 mM) was used as a reference compound. Dotted line represents the amount of HOS-induced ATP release from B16-BL6 cells (*cf.* **Error! Reference source not found.D**). Grey dots represent individual experiments. Data are shown as mean ± SEM. $^{\$}$P value compared to Tyrode condition.

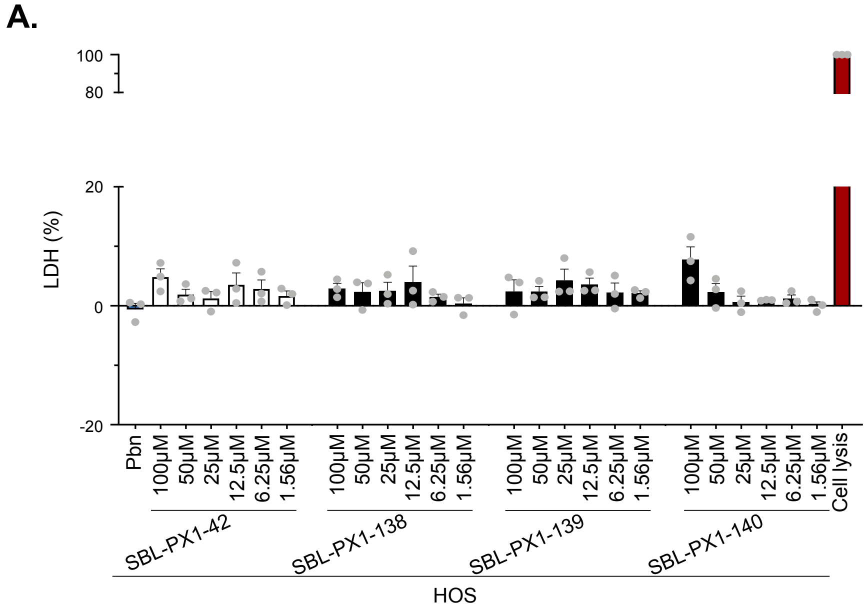

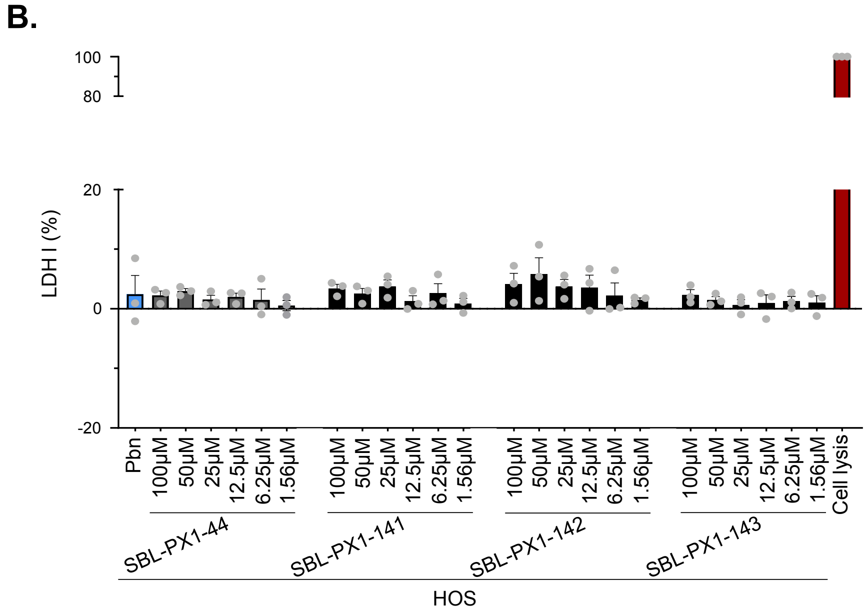

Figure S6. Cytotoxicity of compounds **SBL-PX1-42** (A) and **SBL-PX1-44** (B) with their respective analogs in which the triazole-linker has been modulated (see **Table S2** for peptidomimetic sequences). B16-BL6 cells were incubated with abovementioned compounds at concentrations ranging from 100 µM to 1.56 µM. Pbn was used at 2.5 mM. LDH leakage was expressed as % of

maximal leakage induced by cell lysis with 2% Triton X-100. Grey dots represent individual experiments.

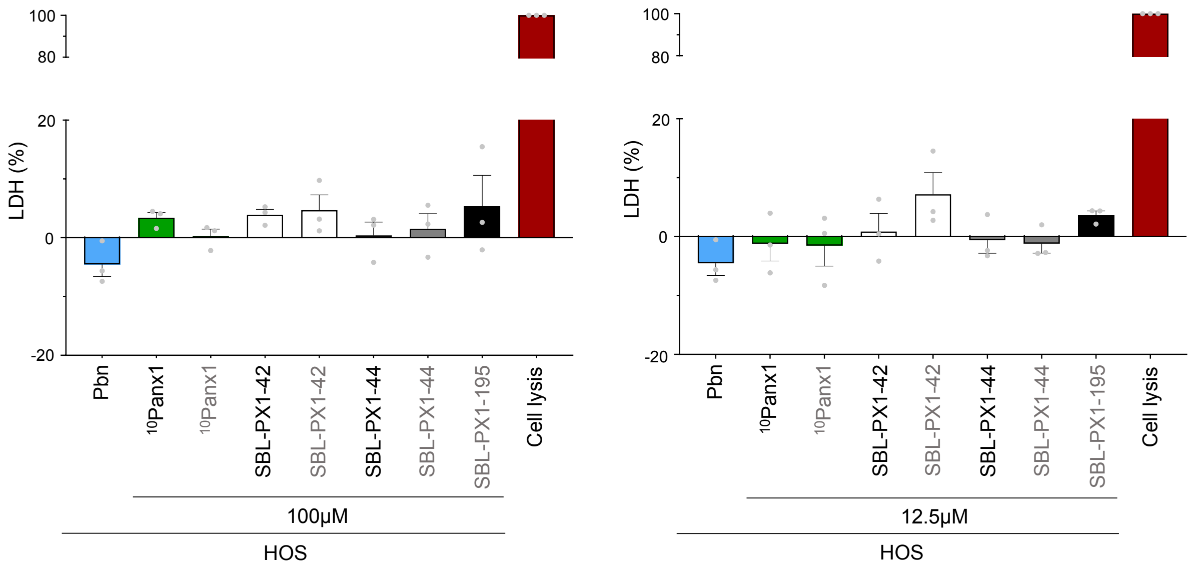

Figure S7. Cytotoxicity of double stapled peptidomimetic **SBL-PX1-195**. LDH leakage by B16-BL6 cells incubated with **SBL-PX1-195** used at 100 μM (*left*) and 12.5 μM (*right*). Compounds ^{10}Panx1, **SBL-PX1-42** and **SBL-PX1-44** were used at 100 μM and 12.5 μM while Pbn was used at 2.5 mM. Compounds in grey – reference peptide and analogs – were tested with 1% DMSO. LDH leakage was expressed as % of maximal leakage induced by cell lysis with 2% Triton X-100. Grey dots represent individual experiments.

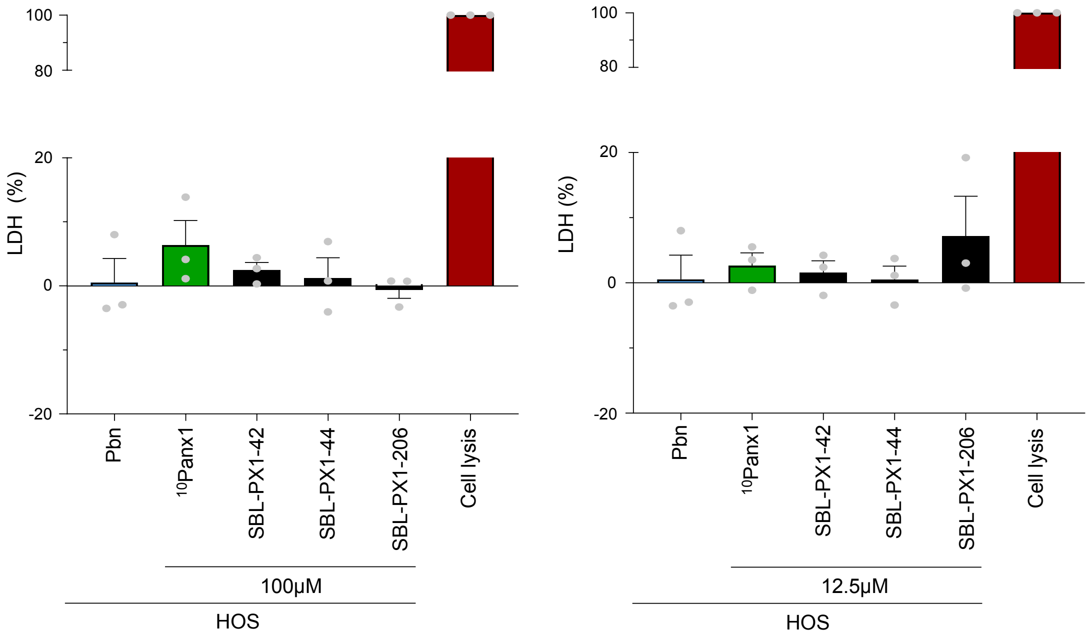

Figure S8. Cytotoxicity of **SBL-PX1-42**, **SBL-PX1-44** and **SBL-PX1-206** in endothelial cells. LDH leakage by EA.hy296 cells incubated with stapled **SBL-PX1-42**, **SBL-PX1-44** and double stapled **SBL-PX1-206** analogs. EA.hy296 cells were incubated with 100 μM (*left*) and 12.5 μM (*right*) of ^{10}Panx1,

single and double stapled ^{10}Panx1 analogs. Pbn was used at 2.5 mM. LDH leakage was expressed as % of maximal leakage induced by cell lysis with 2% Saponin.

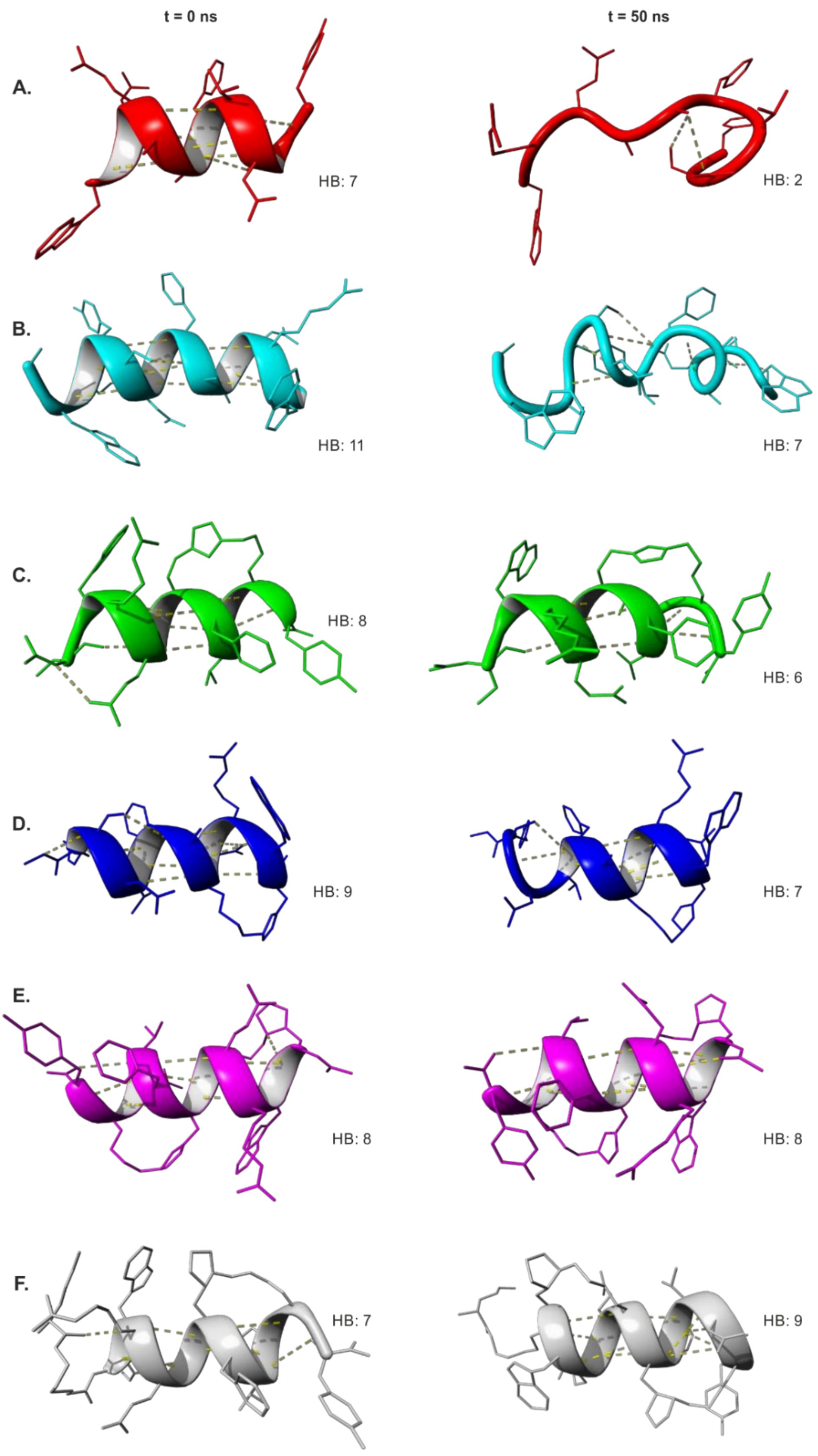

Figure S9. Representation of the molecular dynamics at t = 0 ns (*left*) and t = 50 ns (*right*) of A) [10]Panx1; B) **H1**; C) **SBL-PX1-42**; D) **SBL-PX1-44**; E) **SBL-PX1-195** and F) **SBL-PX1-206**. Hydrogen bond interactions (HB) are represented as yellow dashed lines and their numbers are written next to the structures.

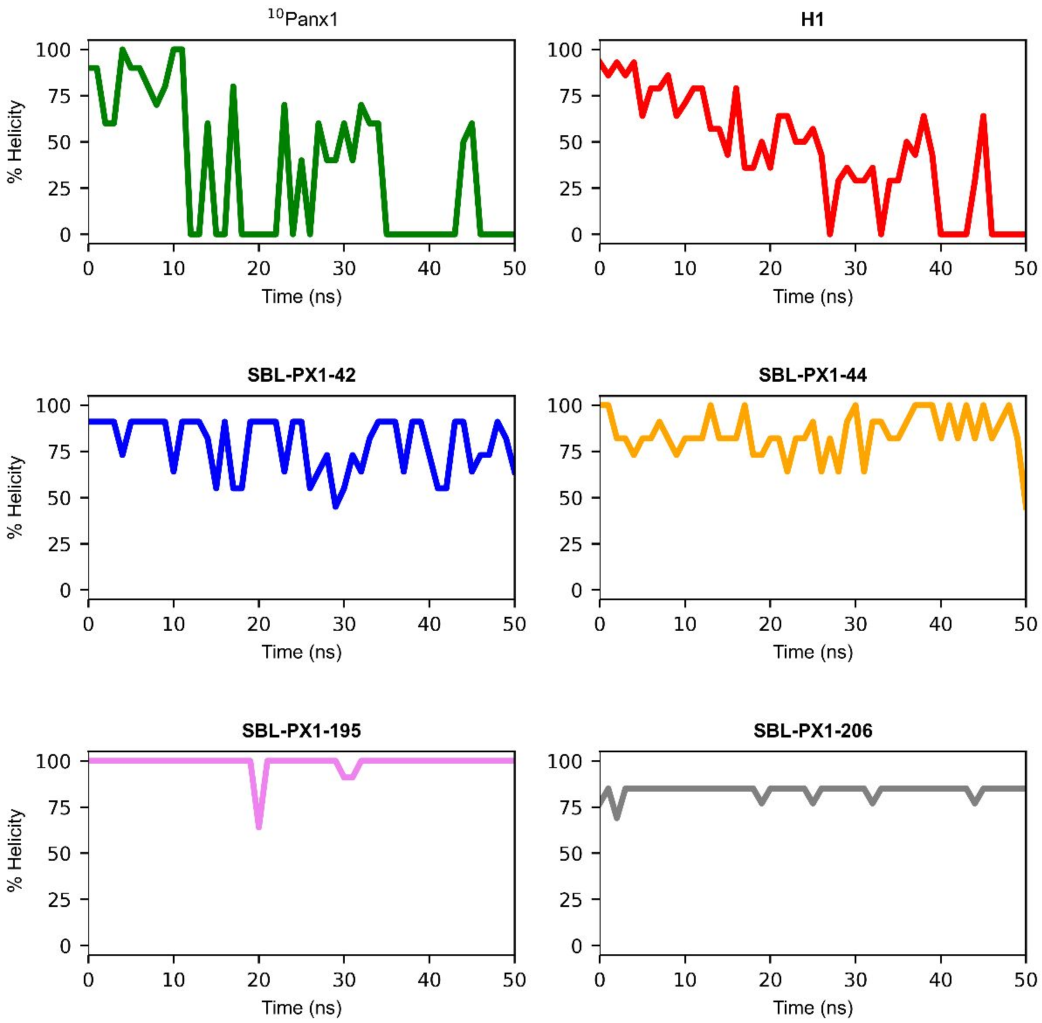

Figure S10. Helicity in function of time obtained for linear and cyclic sequences during molecular dynamics calculations over a period of 50 ns.

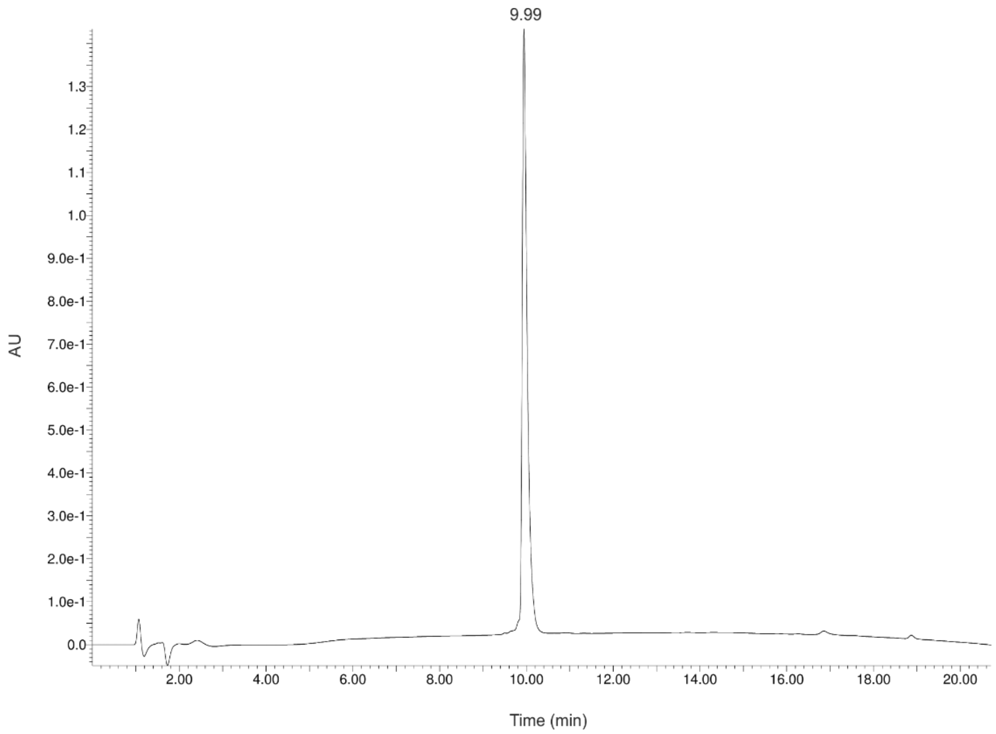

Figure S11. LC spectrum of compound **SBL-PX1-206** (peak at 9.99 min) as reference for *in vitro* plasma stability experiment.

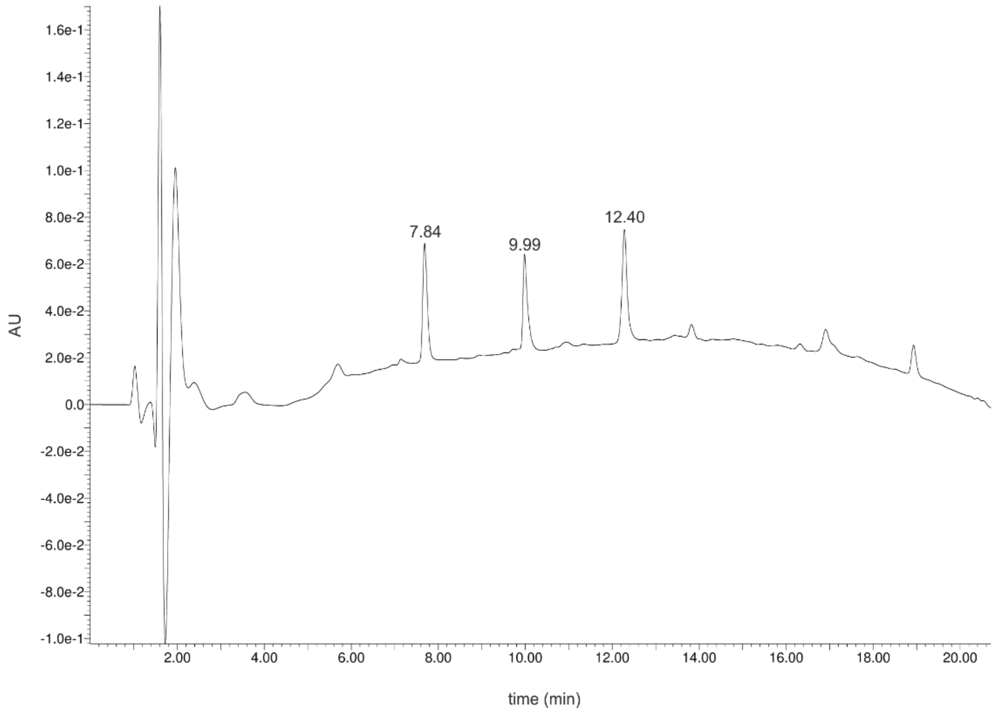

Figure S12. LC spectrum of compound **SBL-PX1-206** (peak at 9.99 minutes) in human plasma at 37 °C during *in vitro* plasma stability experiment at t = 0 minute. Peaks at 7.84 and 12.40 minutes are unidentified proteins from human plasma.

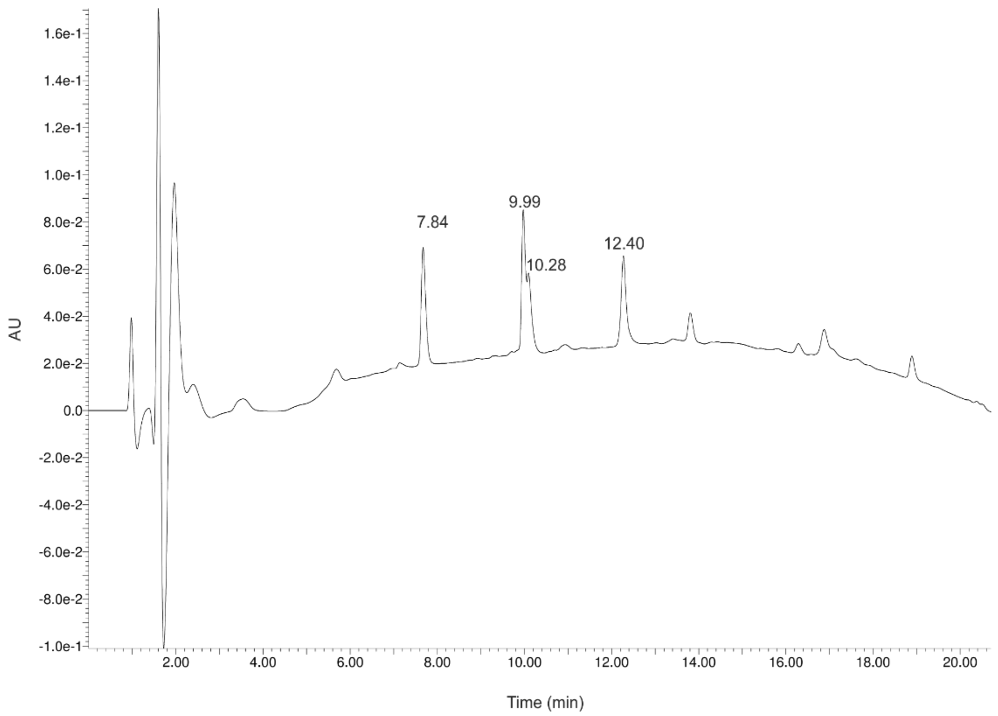

Figure S13. LC spectrum of compound **SBL-PX1-206** (peak at 9.99 minutes) in human plasma at 37 °C during *in vitro* plasma stability experiment at t = 8 hours. Peak at 10.28 minutes has been identified as **SBL-PX1-206**'s metabolite where Lysine extremity has been cleaved from enzymatic degradation. Peaks at 7.84 and 12.40 minutes are unidentified proteins from human plasma.

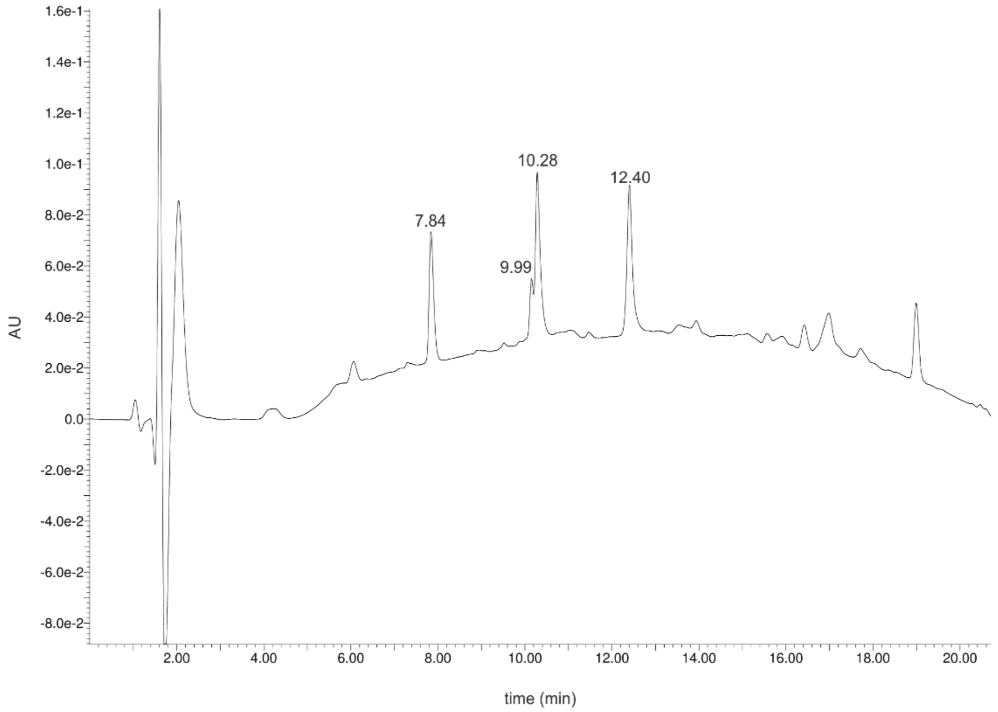

Figure S14. LC spectrum of compound **SBL-PX1-206** (peak at 9.99 minutes) in human plasma at 37 °C during *in vitro* plasma stability experiment at t = 24 hours. The peak at 10.28 minutes has been identified as **SBL-PX1-206**'s metabolite where Lysine extremity has been cleaved from enzymatic degradation. Peaks at 7.84 and 12.40 minutes are unidentified proteins from human plasma.

II. PEPTIDE CHARACTERIZATION

Characterization

Table S2. Analytical data of synthesized peptides

	Sequence[(a)]	Formula	MW[(c)] (g.mol^{-1})	R_t[(b)] (min)	Yield (%)	Purity (%)	HRMS calculated	HRMS found
**H1**	H-Ser-Trp-Arg-Gln-Ala-Ala-Phe-Val-Asp-Ser-Tyr-Cys-Trp-Ala-OH	$C_{78}H_{104}N_{20}O_{21}S$	1917.92	1.87	6	96	1711.7304	1711.7303
[10]Panx1	H-Trp-Arg-Gln-Ala-Ala-Phe-Val-Asp-Ser-Tyr-OH	$C_{58}H_{79}N_{15}O_{16}$	1470.41	1.68	43	>99	1242.5907	1242.5928
**SBL-PX1-38**	Ac-Ser-Trp-Arg-Gln-Ala-Ala-Phe-c[Pra-Asp-Ser-Tyr-Azk]-NH_2	$C_{69}H_{93}N_{21}O_{19}$	1634.65	1.84	7	>99	1520.7035	1520.6967
**SBL-PX1-39**	Ac-Ser-Trp-Arg-Gln-Ala-Ala-Phe-c[Pra-Asp-Ser-Tyr-Azo]-NH_2	$C_{68}H_{91}N_{21}O_{19}$	1620.63	1.78	7	98	1506.6879	1506.6810
**SBL-PX1-40**	Ac-Ser-Trp-Arg-Gln-Ala-Ala-c[Pra-Val-Asp-Ser-Azk]-NH_2	$C_{56}H_{84}N_{20}O_{17}$	1423.43	1.69	6	>99	1309.6401	1309.6400
**SBL-PX1-41**	Ac-Ser-Trp-Arg-Gln-Ala-Ala-c[Pra-Val-Asp-Ser-Azo]-NH_2	$C_{55}H_{82}N_{20}O_{17}$	1409.41	1.48	9	98	1317.6064[(d)]	1317.6108
**SBL-PX1-42**	Ac-Ser-Trp-Arg-Gln-Ala-c[Pra-Phe-Val-Asp-Azk]-Tyr-NH_2	$C_{68}H_{92}N_{20}O_{17}$	1575.63	1.93	19	>99	1461.7028	1461.7056
**SBL-PX1-43**	Ac-Ser-Trp-Arg-Gln-Ala-c[Pra-Phe-Val-Asp-Azo]-Tyr-NH_2	$C_{67}H_{90}N_{20}O_{17}$	1561.60	1.86	11	>99	1447.6871	1447.6887
**SBL-PX1-44**	Ac-c[Pra-Trp-Arg-Gln-Azk]-Ala-Phe-Val-Asp-Ser-Tyr-NH_2	$C_{68}H_{92}N_{20}O_{17}$	1575.63	1.89	11	>99	1483.6847[(d)]	1483.6826
**SBL-PX1-45**	Ac-c[Pra-Trp-Arg-Gln-Azo]-Ala-Phe-Val-Asp-Ser-Tyr-NH_2	$C_{67}H_{90}N_{20}O_{17}$	1561.61	1.78	8	98	1447.6871	1447.6923
**SBL-PX1-138**	Ac-Ser-Trp-Arg-Gln-Ala-c[Azk-Phe-Val-Asp-Pra]-Tyr-NH_2	$C_{68}H_{92}N_{20}O_{17}$	1575.63	1.92	4	99	742.3463[(e)]	742.3400
**SBL-PX1-139**	Ac-Ser-Trp-Arg-Gln-Ala-c[*h*Pra-Phe-Val-Asp-Azk]-Tyr-NH_2	$C_{69}H_{94}N_{20}O_{17}$	1589.66	1.92	26	99	1497.7003	1497.7034
**SBL-PX1-140**	Ac-Ser-Trp-Arg-Gln-Ala-c[D-Pra-Phe-Val-Asp-Azk]-Tyr-NH_2	$C_{68}H_{92}N_{20}O_{17}$	1575.63	1.91	15	99	1461.7028	1461.7056
**SBL-PX1-141**	Ac-c[Azk-Trp-Arg-Gln-Pra]-Ala-Phe-Val-Asp-Ser-Tyr-NH_2	$C_{68}H_{92}N_{20}O_{17}$	1575.63	1.87	3	97	1461.7028	1461.7114
**SBL-PX1-142**	Ac-c[*h*Pra-Trp-Arg-Gln-Azk]-Ala-Phe-Val-Asp-Ser-Tyr-NH_2	$C_{69}H_{94}N_{20}O_{17}$	1589.66	1.91	18	99	1475.7184	1475.7266
**SBL-PX1-143**	Ac-c[D-Pra-Trp-Arg-Gln-Azk]-Ala-Phe-Val-Asp-Ser-Tyr-NH_2	$C_{68}H_{92}N_{20}O_{17}$	1575.63	1.94	8	96	1461.7028	1461.6948
**SBL-PX1-195**	Ac-c[Pra-Trp-Arg-Gln-Azk]-c[Pra-Phe-Val-Asp-Azk]-Tyr-NH_2	$C_{73}H_{97}N_{23}O_{16}$	1666.75	1.85	23	99	1552.7562	1552.7632

**SBL-PX1-206**	H-Lys-Ahx-c[Pra-Trp-Arg-Gln-Azk]-c[Pra-Phe-Val-Asp-Azk]-Tyr-NH_2	$C_{83}H_{118}N_{26}O_{17}$	2094.09	1.77	12	99	1751.9247	1751.9261
**SBL-PX1-214**	H-Lys-Ahx-Trp-Arg-Gln-Ala-Ala-Phe-Val-Asp-Ser-Tyr-OH	$C_{70}H_{102}N_{18}O_{18}$	1825.76	1.88	7	98	1505.7517	1505.7600

(a) Residues between squared brackets are part of the peptide macrocycle; Azk, Azo, Pra and *h*-Pra stand for azido-lysine, azido-ornithine, propargylglycine and *homo*-propargylglycine precursor respectively; (b) as TFA salts; (c) purity and retention time (R_t) are based on reversed-phase HPLC analysis; (d) $[M+Na]^+$; (e) $[M+Na+H]^{2+}$.

RP-HPLC chromatograms of the synthesized peptidomimetics

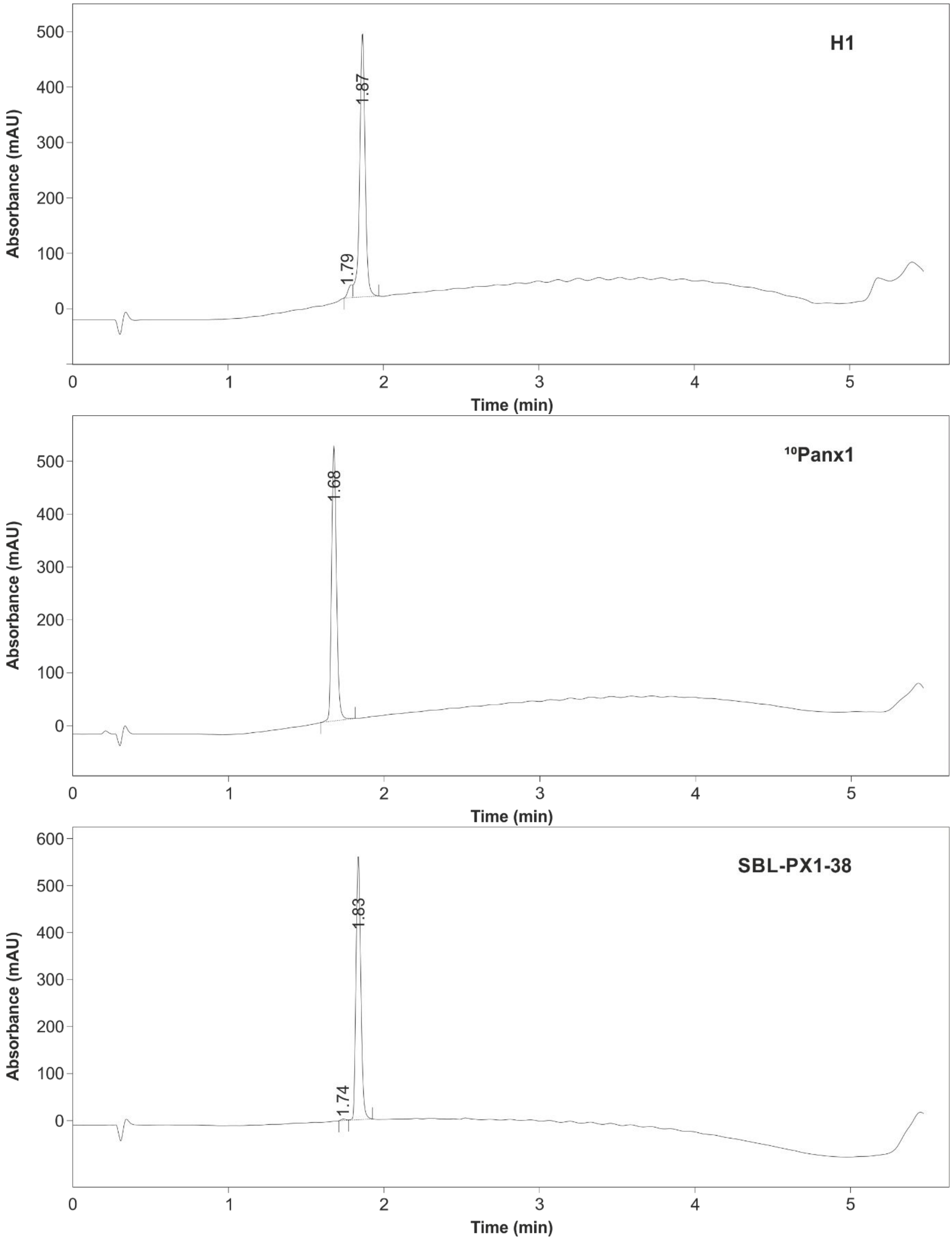

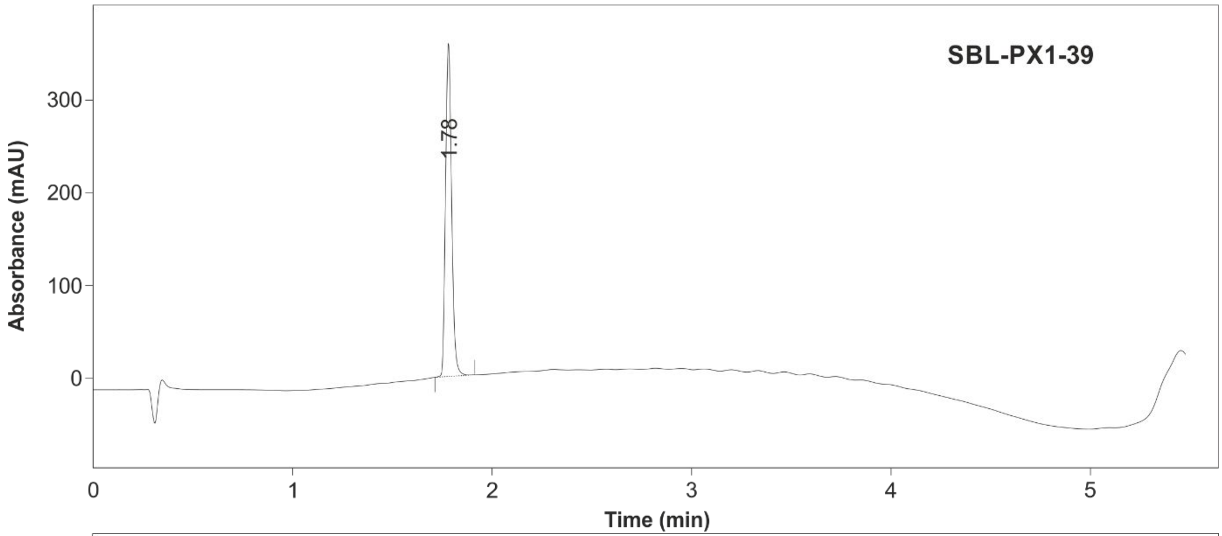
SBL-PX1-39
Absorbance (mAU)
Time (min)
1.78

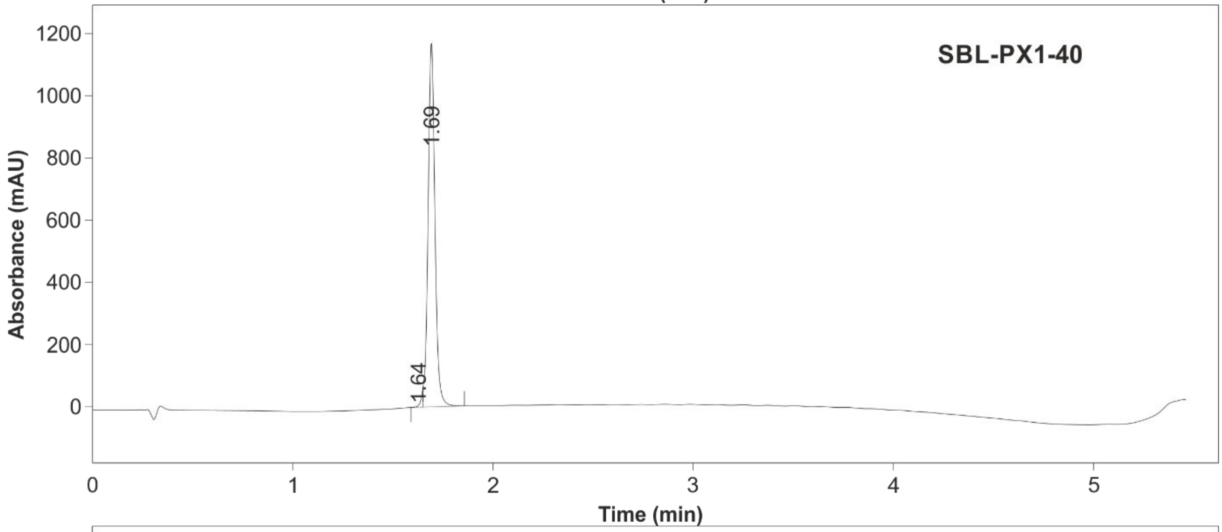
SBL-PX1-40
Absorbance (mAU)
Time (min)
1.69
1.64

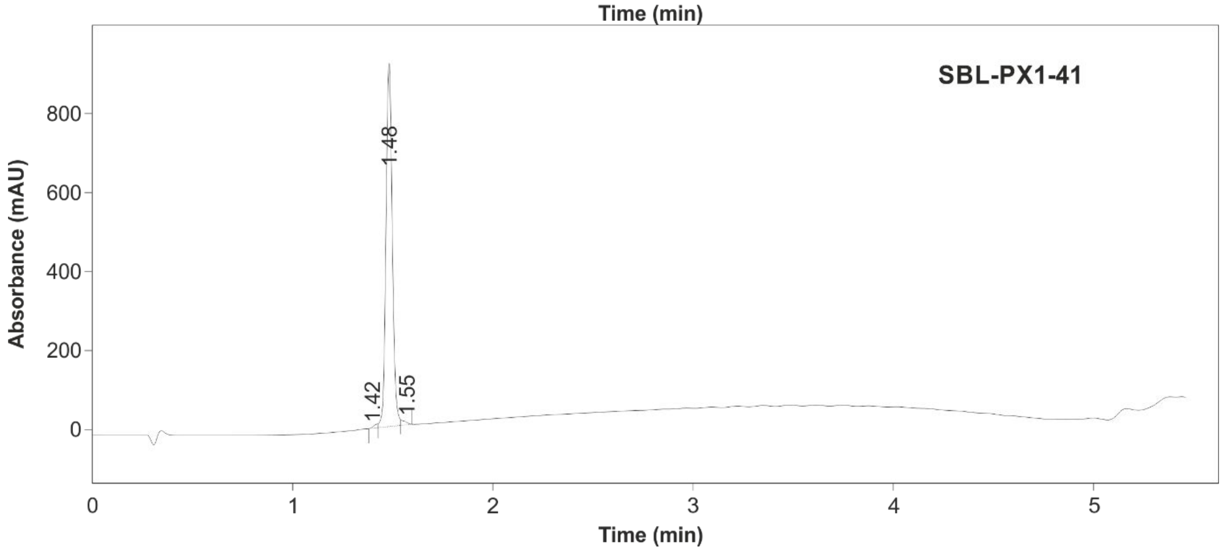
SBL-PX1-41
Absorbance (mAU)
Time (min)
1.48
1.42
1.55

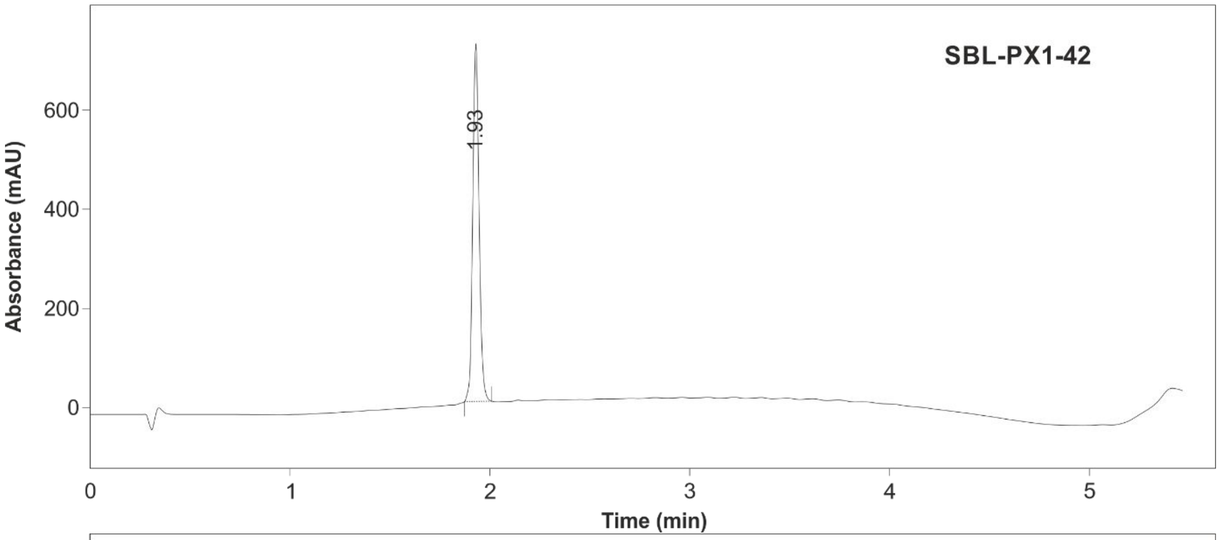
SBL-PX1-42
1.93
Absorbance (mAU)
Time (min)

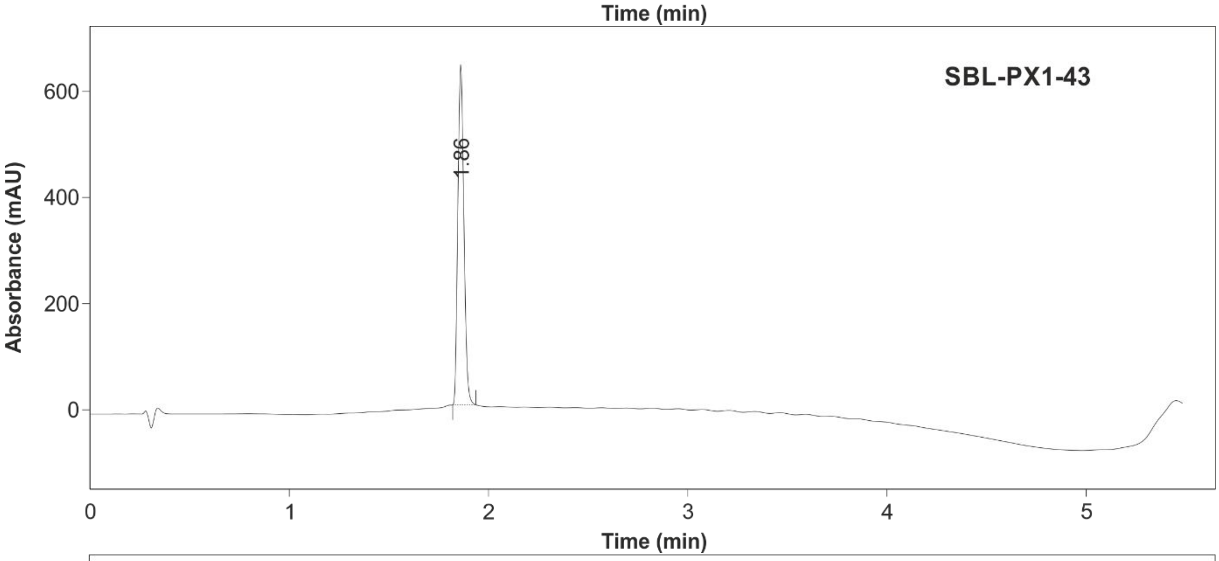
SBL-PX1-43
1.86
Absorbance (mAU)
Time (min)

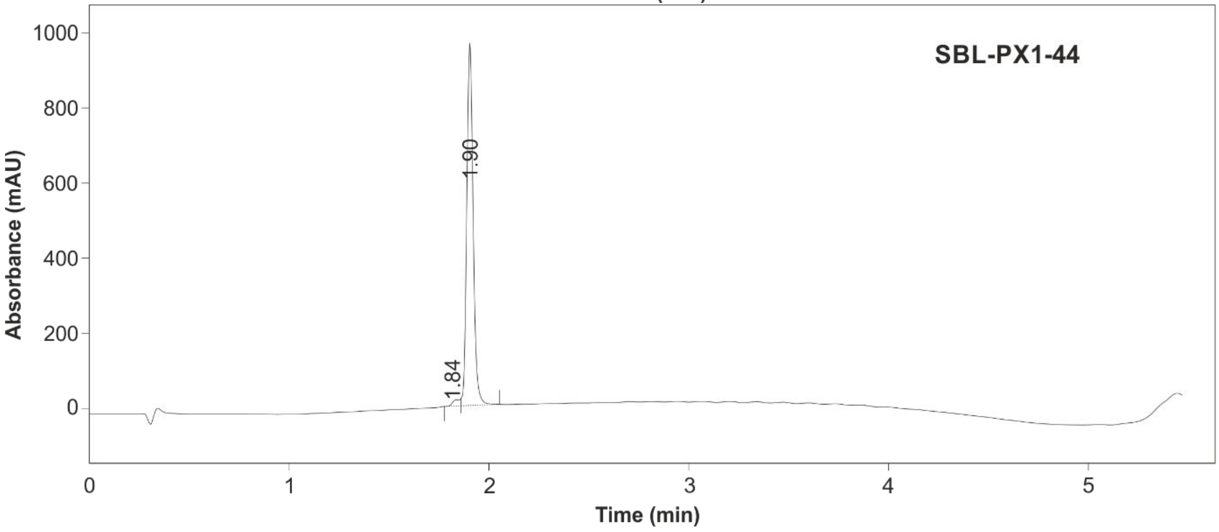
SBL-PX1-44
1.90
1.84
Absorbance (mAU)
Time (min)

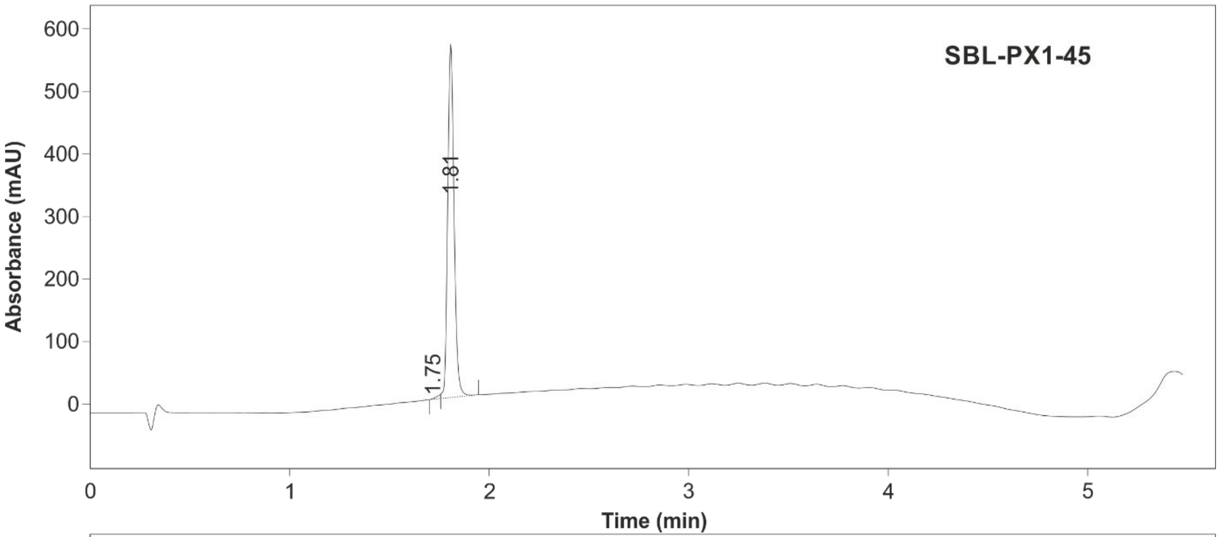
SBL-PX1-45
Absorbance (mAU)
Time (min)
1.81
1.75
600
500
400
300
200
100
0
0
1
2
3
4
5

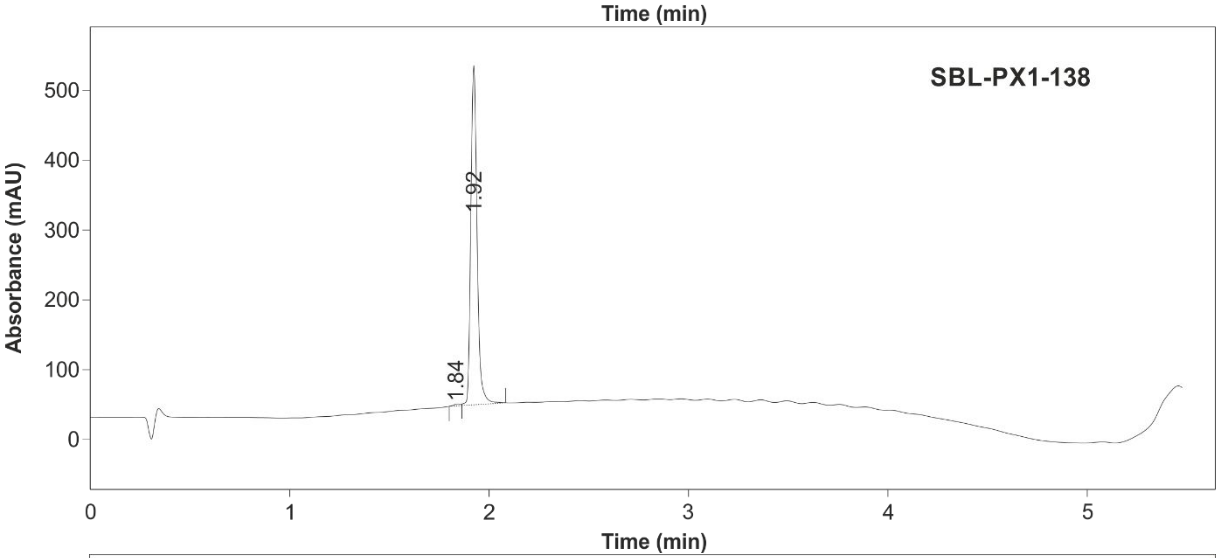
SBL-PX1-138
Absorbance (mAU)
Time (min)
1.92
1.84
500
400
300
200
100
0
0
1
2
3
4
5

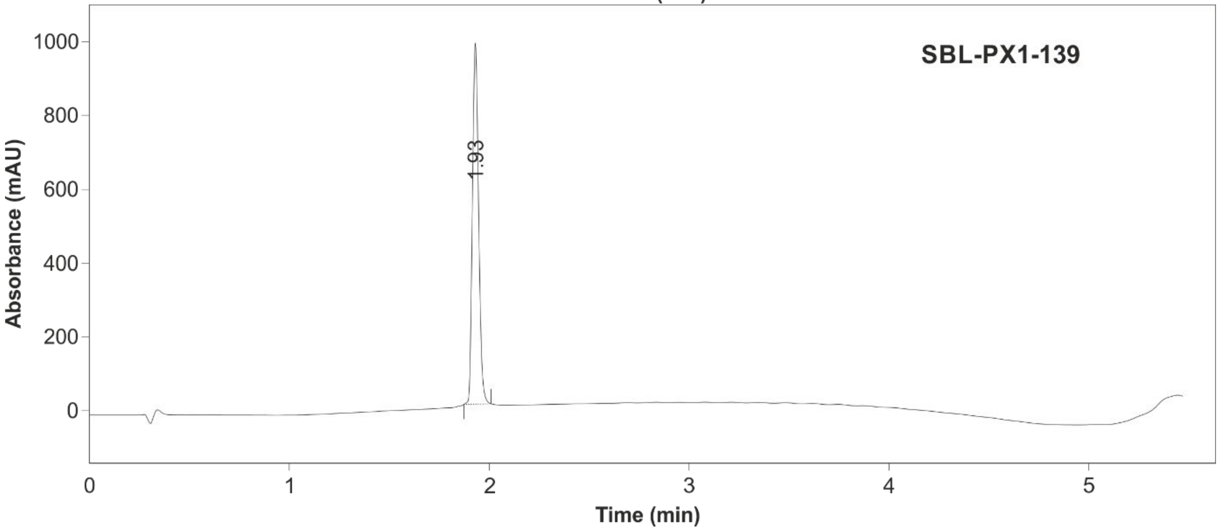
SBL-PX1-139
Absorbance (mAU)
Time (min)
1.93
1000
800
600
400
200
0
0
1
2
3
4
5

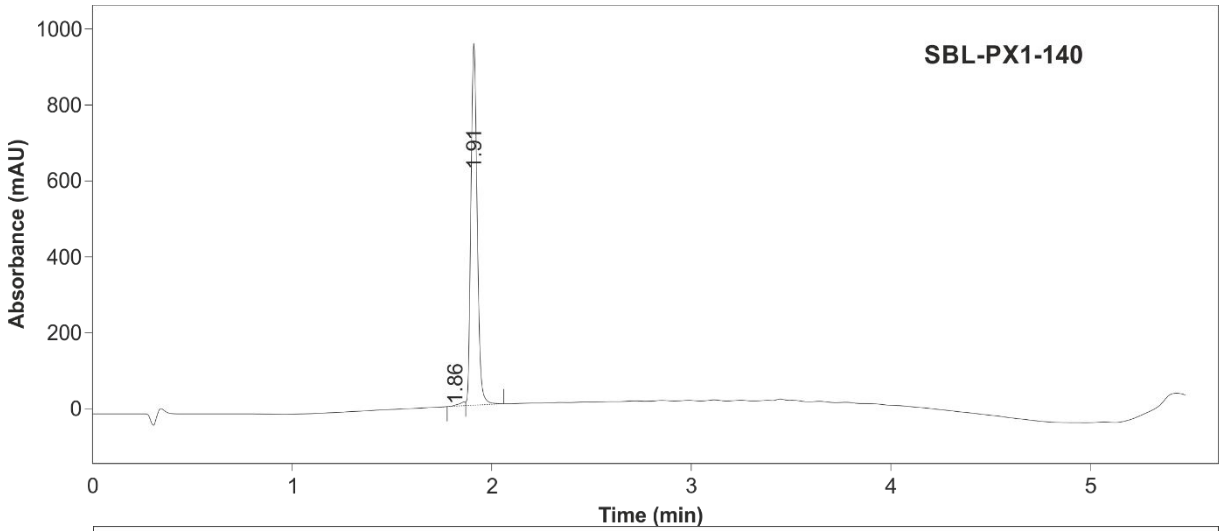
SBL-PX1-140
Absorbance (mAU)
Time (min)
1.91
1.86

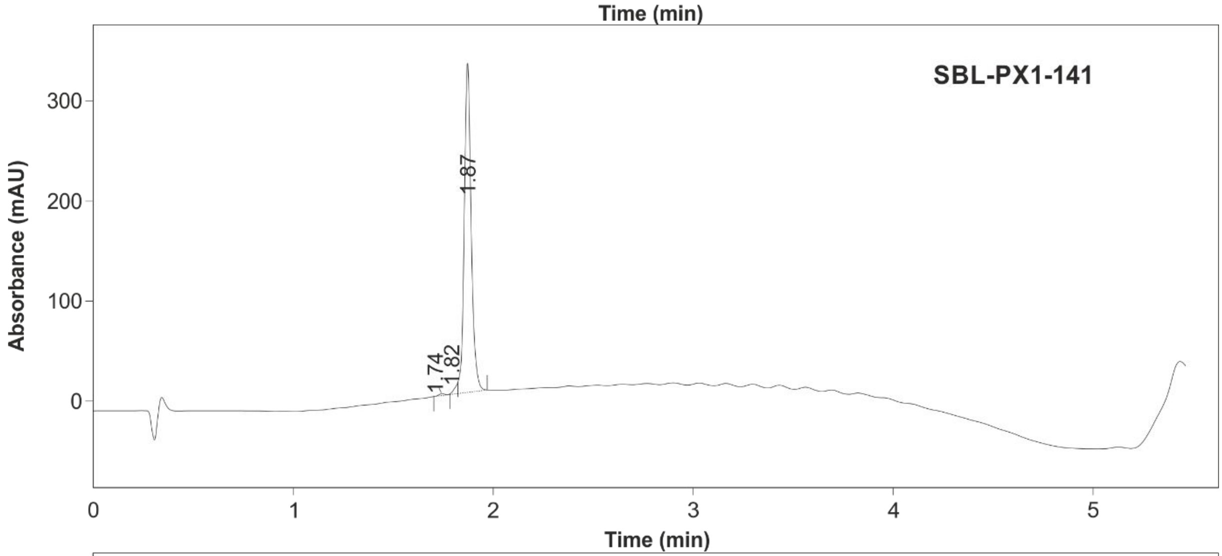
SBL-PX1-141
Absorbance (mAU)
Time (min)
1.87
1.74
1.82

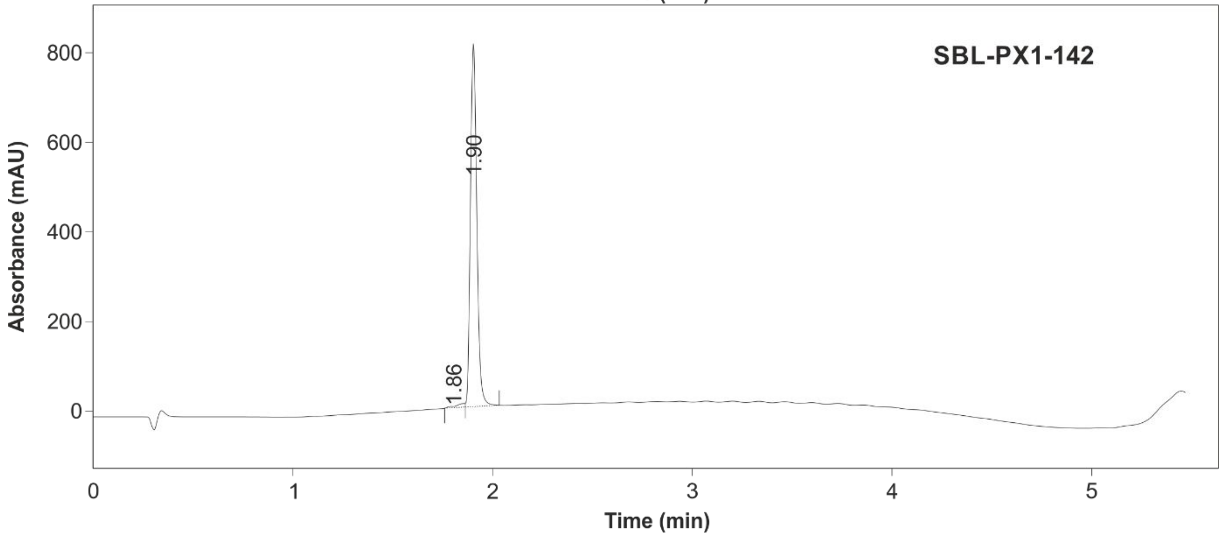
SBL-PX1-142
Absorbance (mAU)
Time (min)
1.90
1.86

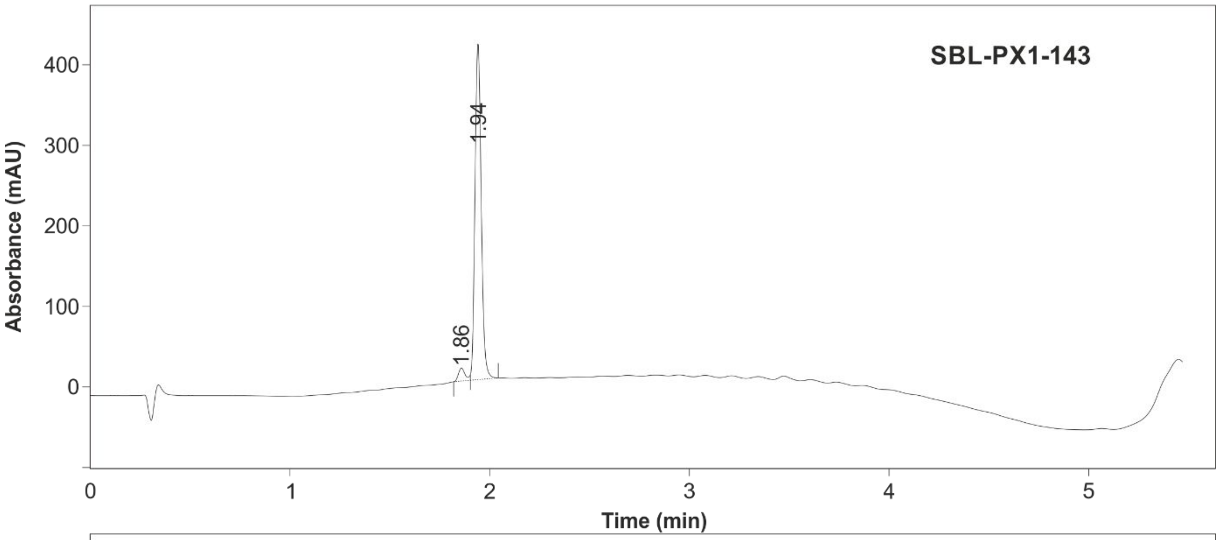
SBL-PX1-143
1.94
1.86
Absorbance (mAU)
Time (min)

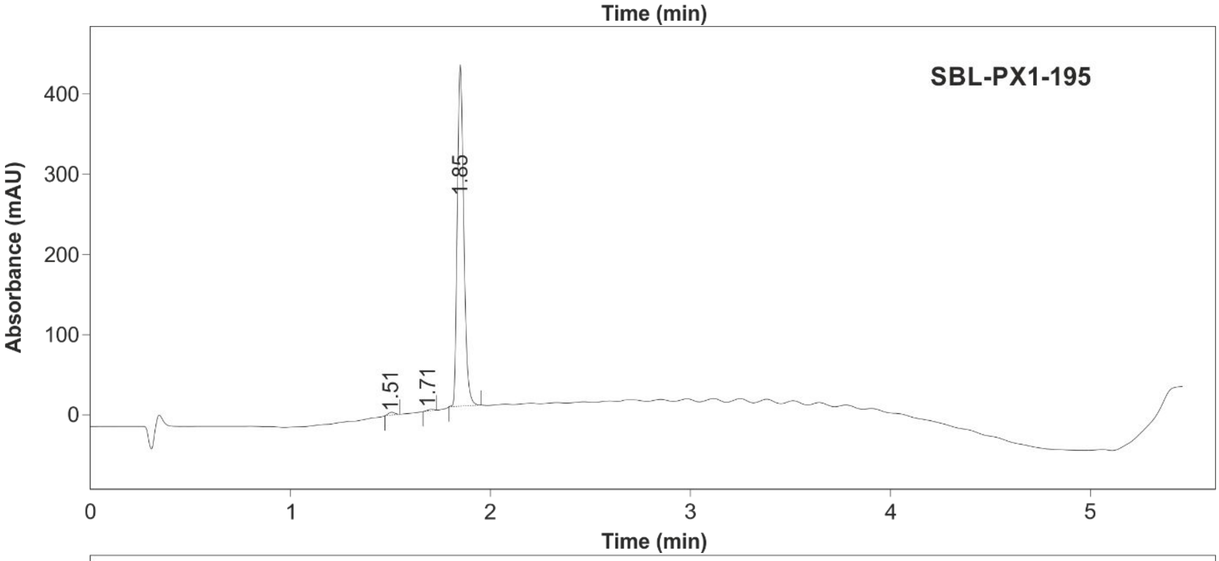
SBL-PX1-195
1.85
1.51
1.71
Absorbance (mAU)
Time (min)

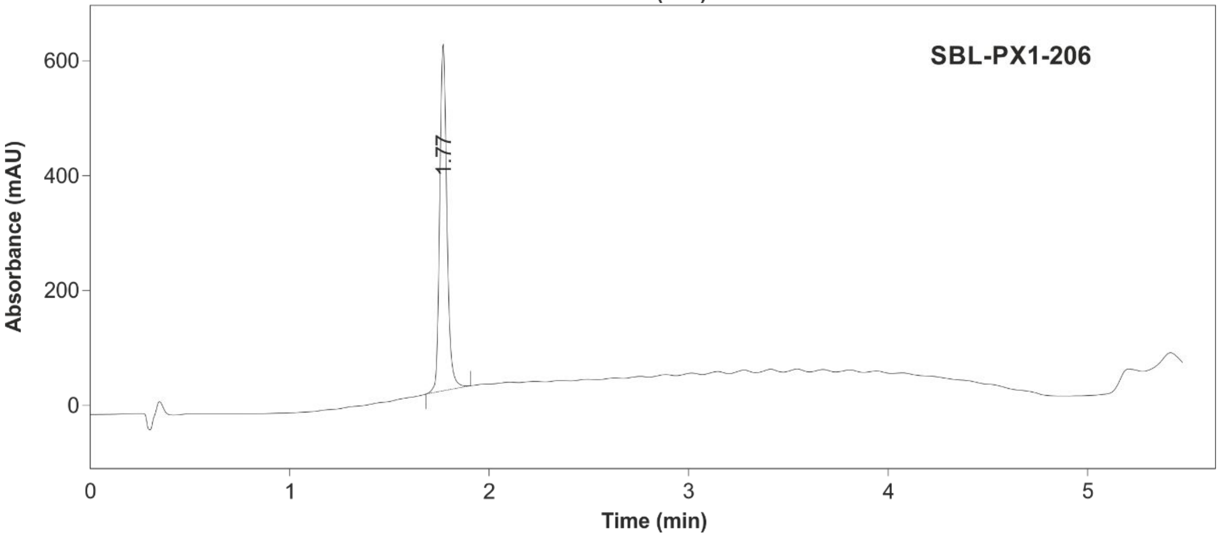
SBL-PX1-206
1.77
Absorbance (mAU)
Time (min)

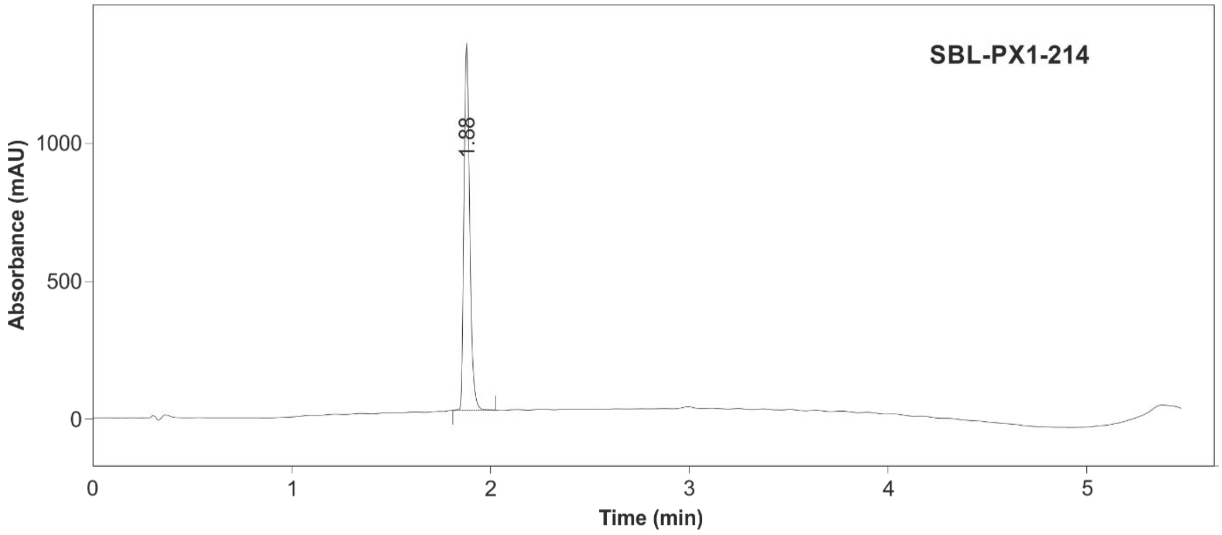

III. CIRCULAR DICHROISM (CD) SPECTROSCOPY

Helix content calculation

The helix content of each peptide was determined as previously reported[ii] from the mean residue helicity $[\theta]_{222}$ (in deg $cm^2.dmol^{-1}$) at 222 nm using formula **(1)**:

$$[\theta]_{222} = (\theta_{222} * MW) / (10 * n * C * l) \qquad \textbf{(1)}$$

with θ_{222} the measured ellipticity at 222 nm, MW the molecular weight of the peptide ($g.mol^{-1}$), n the number of amino acids in the peptide, C the peptide concentration ($mg.mL^{-1}$) and l the path length.

The maximum mean ellipticity was then calculated using undermentioned formula **(2)**:

$$[\theta]_{max} = (-44\,000 + 250\,T) * (1 - k / n) \qquad \textbf{(2)}$$

where T is the temperature (°C), k is standardly equals to 3 for *N*-acetylated peptides and n number of amino acid residues in the peptide.

Percent α-helicity was then determined using the following equation **(3)**:

$$\%\ helicity = ([\theta]_{222} / [\theta]_{max}) * 100 \qquad \textbf{(3)}$$

CD spectra of reported peptidomimetics

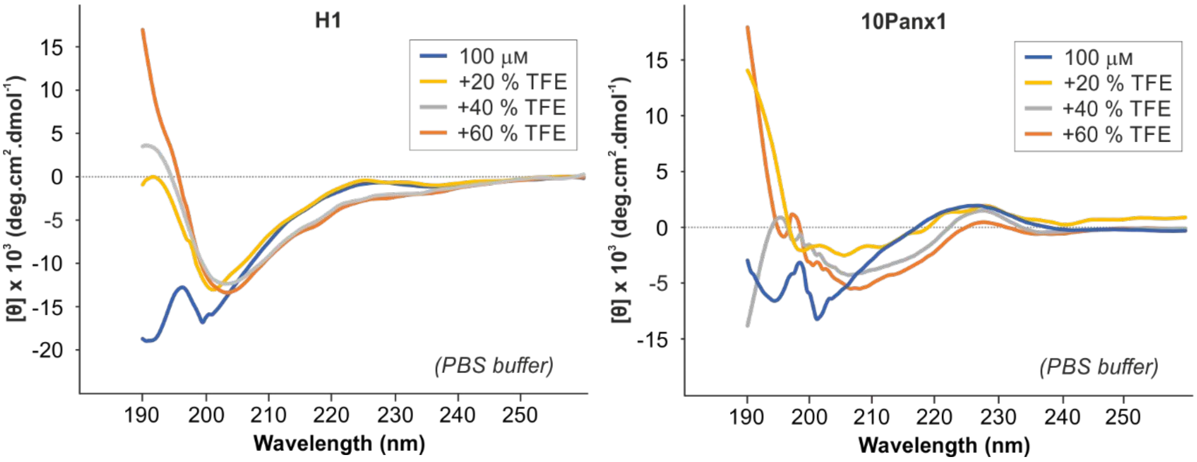

Figure S15. CD spectra of the peptide **H1** found in the extracellular domain of Panx1 (*left*) and [10]Panx1 reference peptide (*right*). Peptide sequences can be found in **Table S2**. All CD spectra were measured in PBS (pH 7.4) at room temperature at 100 μM with an increased concentration of TFE.

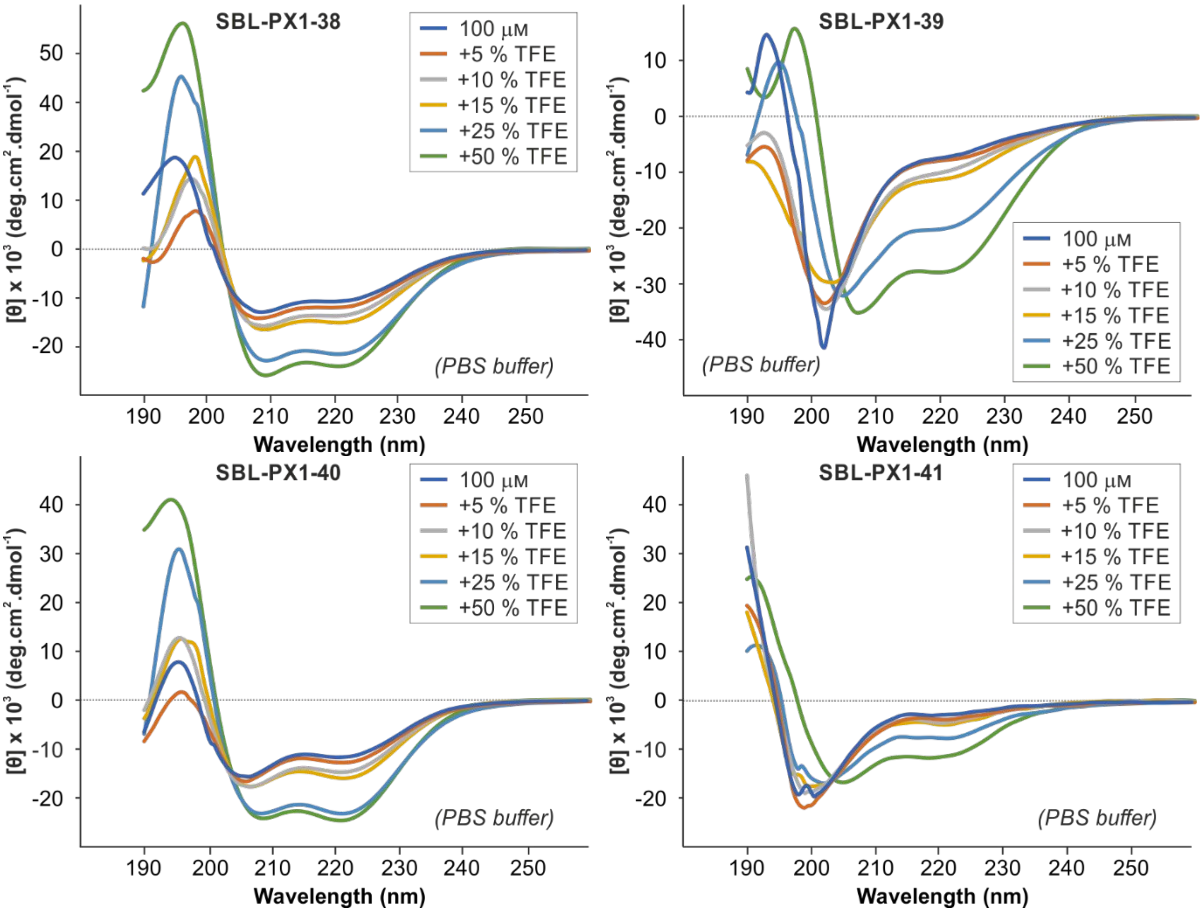

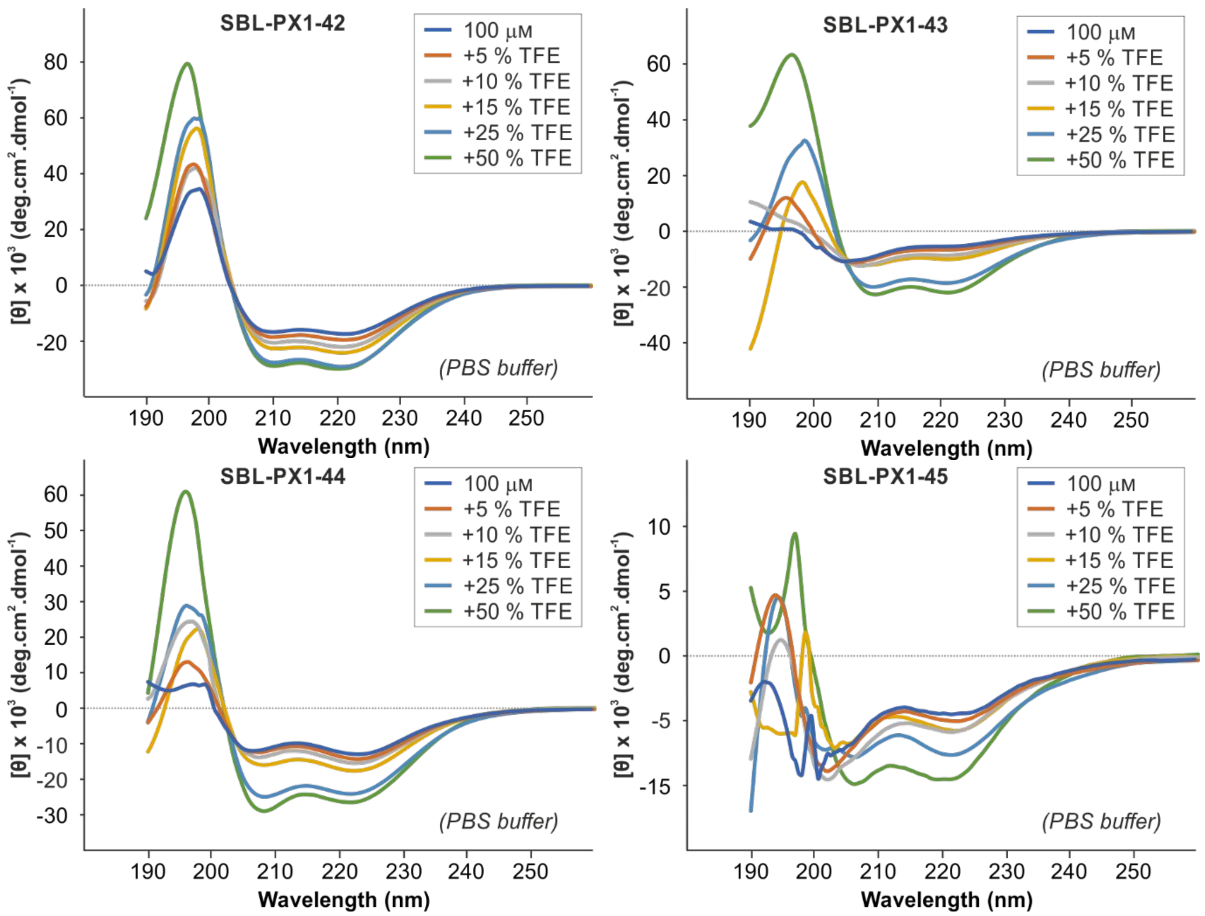

Figure S16. CD spectra of [10]Panx1-based stapled compounds from the (*i*,*i+4*) cyclization scanning. Peptidomimetic sequences can be found in **Table S2**. All spectra were measured in PBS (pH 7.4) at room temperature at 100 µM with an increased concentration of TFE.

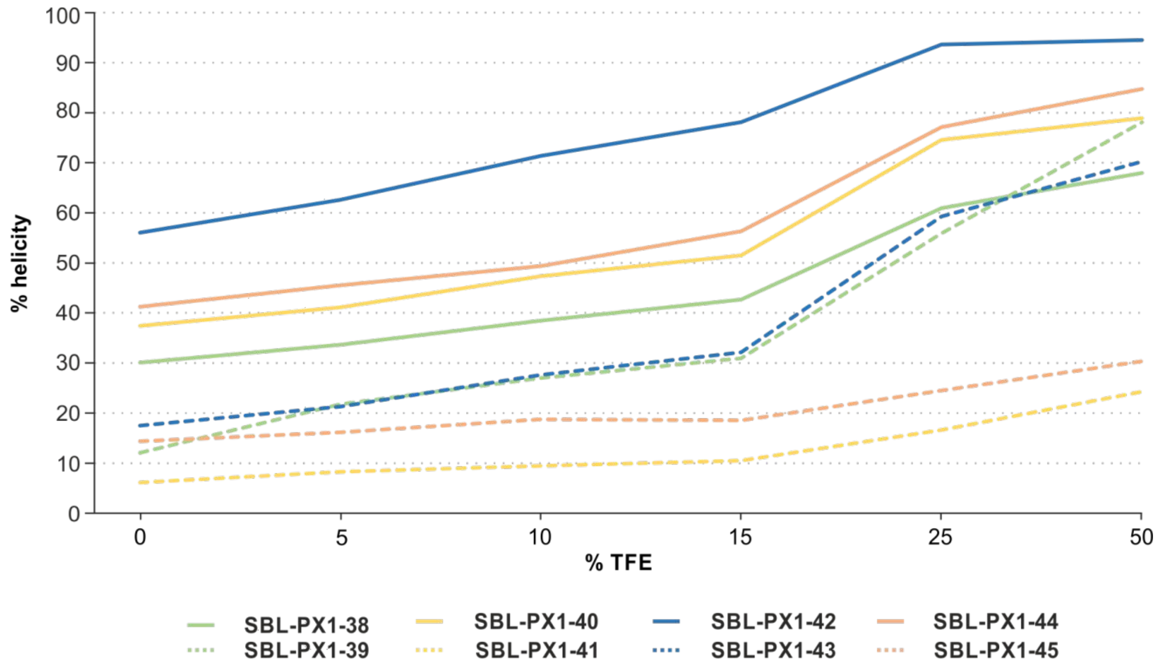

Figure S17. Graph showing the percent helicity in function of the concentration of TFE present in recorded CD samples presented in **Figure S16**. Helicity was calculated from the mean residue ellipticity at 222 nm (θ_{222}) as abovementioned.

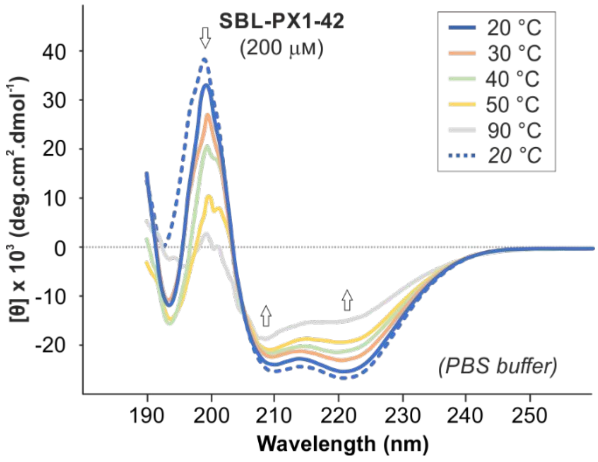

Figure S18. CD spectra of compound **SBL-PX1-42** where the temperature have been gradually modulated from 20 °C to 90 °C (full lines) before being cool down to 20 °C (dash line). Spectra was measured in PBS (pH 7.4) at 200 µM. Peptidomimetic sequence can be found in **Table S2**.

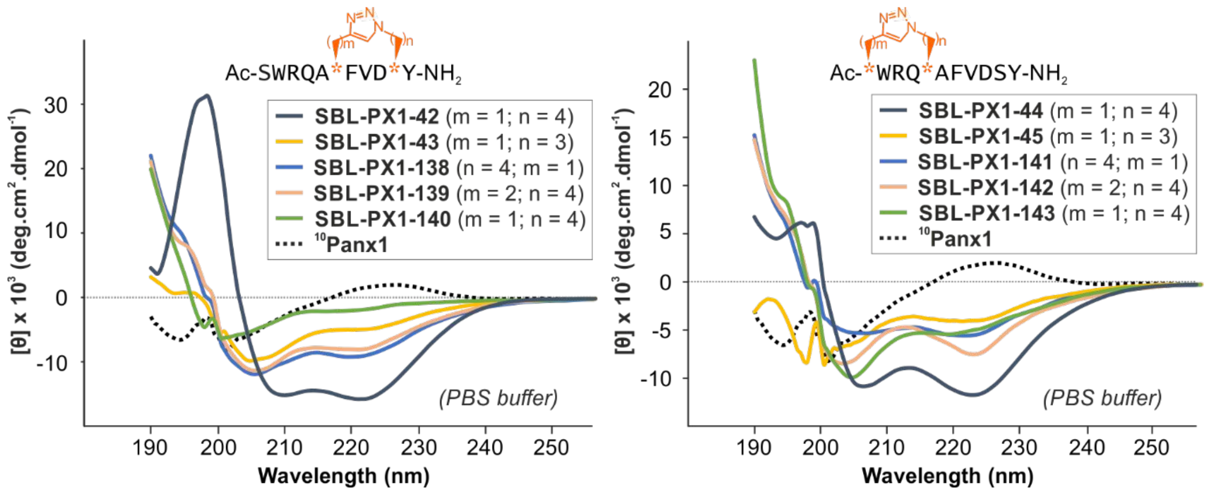

Figure S19. Overlay of the CD spectra of **SBL-PX1-42**-based (*left*) and **SBL-PX1-44** (*right*)-based compounds where the orientation, the length and the stereochemistry of the linker have been modulated. All spectra were measured in PBS (pH 7.4) at room temperature at 100 µM concentration. Peptidomimetic sequences of the corresponding analogs are written on the top. The azidated and alkynylated precursor residues are represented by orange stars and the corresponding number of their methylene units are written as (m, n) values.

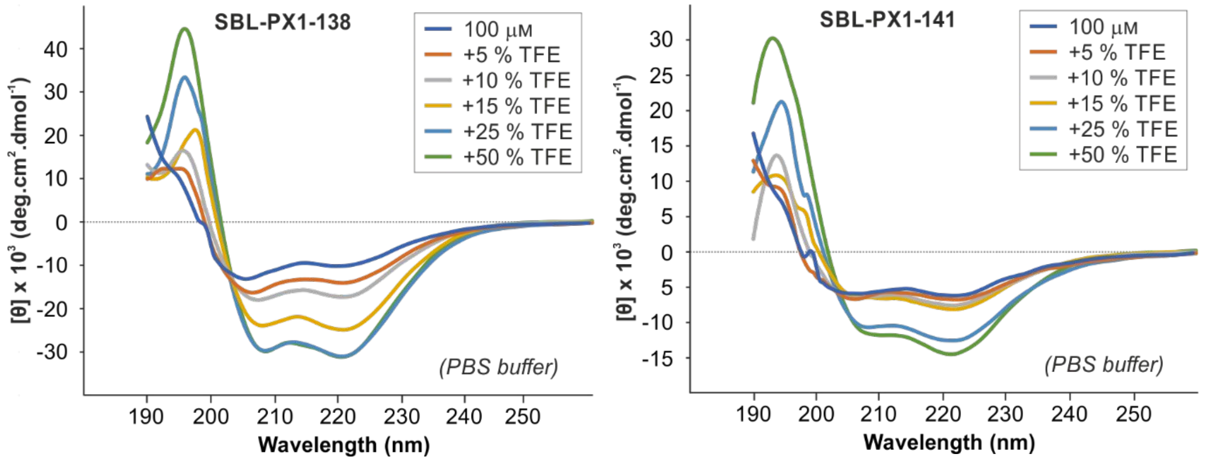

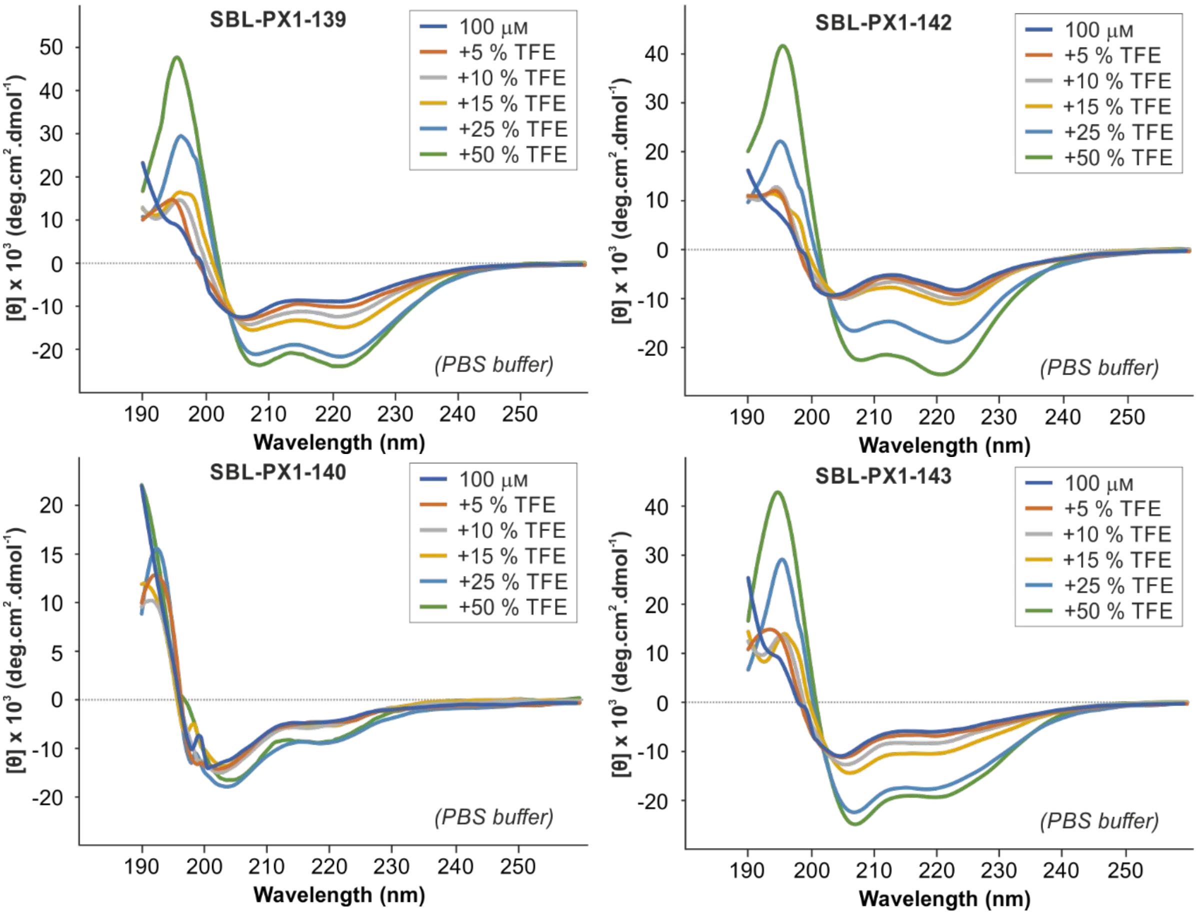

Figure S20. CD spectra of **SBL-PX1-42**-based (*left*) and **SBL-PX1-44** (*right*)-based compounds where the orientation, the length and the stereochemistry (1st, 2nd and 3rd row, respectively) of the linker have been further modulated. Peptidomimetic sequences can be found in **Table S2** and **Figure S19**. All spectra were measured in PBS (pH 7.4) at room temperature at 100 μM with an increased concentration of TFE.

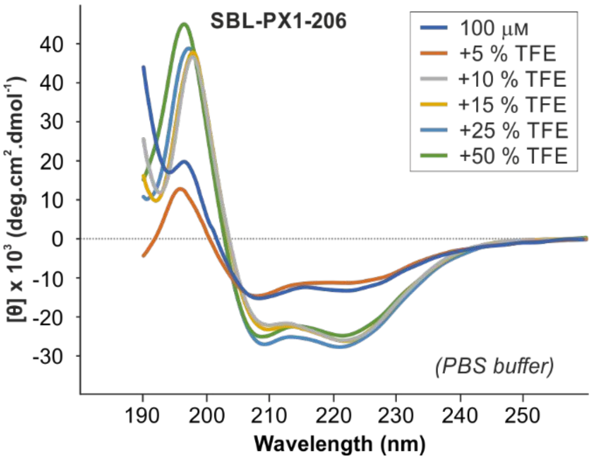

Figure S21. CD spectra of double stapled compound **SBL-PX1-206**. Spectra was measured in PBS (pH 7.4) at 100 μM concentration with an increased concentration of TFE. Peptidomimetic sequence can be found in **Table S2**.

IV. References

[i] Ruan, Z.; Orozco, I. J.; Du, J.; Lü, W. Structures of human pannexin 1 reveal ion pathways and mechanism of gating. *Nature* **2020,** *584,* 646–651.

[ii] Kawamoto, S. A.; Coleska, A.; Ran, X.; Yi, H.; Yang, C.-Y.; Wang, S. Design of triazole-stapled BCL9 α-helical peptides to target the β-catenin/B-cell CLL/lymphoma 9 (BCL9) protein–protein interaction. *J. Med. Chem.* **2012,** *55,* 1137−1146.
